# Supplementary material for: DNA damage induces long range changes to duplex structure—a non-protein start to damage detection?
Source: Nucleic Acids Res. 2026 Sep 25;54(18):gkag920. doi: 10.1093/nar/gkag920 (PMC13613070; doi:10.1093/nar/gkag920)
Supplement: Supplementary file 1 [file gkag920_supplemental_file.pdf]

DNA damage induces long range changes to duplex structure - a non-protein start to damage detection?

AUTHORS

Sophie E. Fountain1, Mahmoud A. S. Abdelhamid1,2, David M. Williams1 and Timothy D. Craggs1,3*

[1] School of Mathematical and Physical Sciences, Faculty of Science, The University of Sheffield, Dainton Building, Brook Hill, S3 7HF, UK

[2] Present Address: Exciting Instruments, Block 5, Level 9 Pennine Five Campus, 18 Hawley Street, Sheffield, England, S1 4WP

[3] Exciting Instruments, Block 5, Level 9 Pennine Five Campus, 18 Hawley Street, Sheffield, England, S1 4WP

* To whom correspondence should be addressed. Email: t.craggs@sheffield.ac.uk

Supplementary material	Location
Supplementary Table 1: Damage DNA sequences	p2
Supplementary Table 2: NMS DNA sequences	p3
Supplementary Table 3: Annealing Combinations	P4
Supplementary Table 4-6: Quantum Yield	p5-8
Supplementary Table 7: J-integral	P9
Supplementary Table 8-10: Predicted E	p10
Supplementary Table 11: Lifetime	p11
Supplementary Table 12: Anisotropy	p12
Supplementary Table 13: Summary of quantum yield, J-integral and anisotropy	p13
Supplementary Table 14: Distance Changes in AV mean position	p14-15

Supplementary material	Location
Supplementary Figure 1-9: 2D Histograms	p16-33
Supplementary Figure 10-18: QQ plots, Kolmogorov-Smirnov and Kruskal-Wallis	p34-42
Supplementary Figure 19: Dunn Tests	p43
Supplementary Figure 20-28: QQ plots, Kolmogorov-Smirnov and Kruskal-Wallis	p44-52
Supplementary Figure 29-30: Dunn Tests	p53-54
Supplementary Figure 31-32: Box Plots	p55-56
Supplementary Figure 33: Quantum Yield example calculation	p57
Supplementary Figure 34: Lifetimes	p58-60
Supplementary Figure 35-36: Anisotropies	p61-62
Supplementary Figure 37-39: Dye movement figure	p63-65

Supplementary Table 1: "T" strands were labelled with the donor dye ATTO 550 and "B" strands were labelled with the acceptor dye ATTO 647N. The labelling sites of the donor and acceptor are shown in orange and in purple on the sequence respectively. The damage site is labelled in blue.

Name	Sequence
Top -7	5′-CCTCATTCTTCGTCCCATTACCATACATCCCTAGAGAGTAGAGCCTGCTTCGTGG-3′
Top -12	5′-CCTCATTCTTCGTCCCATTACCATACATCCCTAGAGAGTAGAGCCTGCTTCGTGG-3′
Top -18	5′-CCTCATTCTTCGTCCCATTACCATACATCCCTAGAGAGTAGAGCCTGCTTCGTGG-3′
Bottom +6	5′-CCACGAAGCAGGCTCTACTCTCTAGGGATGTATGGTAATGGGACGAAGAATGAGG-3′
Bottom +9	5′-CCACGAAGCAGGCTCTACTCTCTAGGGATGTATGGTAATGGGACGAAGAATGAGG-3′
Bottom +11	5′-CCACGAAGCAGGCTCTACTCTCTAGGGATGTATGGTAATGGGACGAAGAATGAGG-3′
Half Top -7	5′-CCTCATTCTTCGTCCCATTACCATACATCC-3′
Half Top -12	5′-CCTCATTCTTCGTCCCATTACCATACATCC-3′
Half Top -18	5′-CCTCATTCTTCGTCCCATTACCATACATCC-3′
Bottom +6 (Ribo, 8-oxoG, abasic)	5′-CCACGAAGCAGGCTCTACTCTCTAGGGATGTATGGTAATGGGACGAAGAATGAGG-3′
Bottom +9 (Ribo, 8-oxoG, abasic)	5′-CCACGAAGCAGGCTCTACTCTCTAGGGATGTATGGTAATGGGACGAAGAATGAGG-3′
Bottom +11 (Ribo, 8-oxoG, abasic)	5′-CCACGAAGCAGGCTCTACTCTCTAGGGATGTATGGTAATGGGACGAAGAATGAGG-3′
Half Top (Nick)	5′-CTAGAGAGTAGAGCCTGCTTCGTGG-3′
Half Top (Gap)	5′-TAGAGAGTAGAGCCTGCTTCGTGG-3′

Supplementary Table 2: NMS1,2,3 strands were labelled with the donor dye ATTO 550 and NMS4 strands were labelled with the acceptor dye ATTO 647N. The labelling sites of the donor and acceptor are shown in orange and in purple on the sequence respectively. The damage site is labelled in blue.

Name	Sequence
NMS1	5′-GAGCTGAAAGTGTCGAGTTTGTTTGAGTGTTTGTCTGG-3′
NMS2	5′-GAGCTGAAAGTGTCGAGTTTGTTTGAGTGTTTGTCTGG-3′
NMS3	5′-GAGCTGAAAGTGTCGAGTTTGTTTGAGTGTTTGTCTGG-3′
NMS4	5′-CCAGACAAACACTCAAACAAACTCGACACTTTCAGCTC-3′
NMS4 (Ribo, 8-oxoG, abasic)	5′- CCAGACAAACACTCAAACAAACTCGACACTTTCAGCTC -3′

Supplementary Table 3: Duplex constructs from combinations of constituent oligonucleotides where (X) indicates the respective labelling position from Table S1 and 2 above.

Construct	Donor Dye	Acceptor Dye
Full length duplex (Control)	Top(X)	Bottom(X)
Swapping of deoxyribose to ribose sugar (Ribo)		Bottom(X) (Ribo)
Addition of oxygen on guanine base (8-oxoG)		Bottom(X) (8-oxoG)
Removal of base (Abasic)		Bottom(X) (Abasic)
Break in the backbone (Nick)	Half Top(X) + Half Top (Nick)	Bottom(X)
Removal of a nucleotide (Gap)	Half Top(X) + Half Top (Gap)	Bottom(X)

Construct	Donor Dye	Acceptor Dye
Full length duplex (Control)	NMS1,2,3 (X)	NMS4(X)
Swapping of deoxyribose to ribose sugar (Ribo)		NMS4 (X) (Ribo)
Addition of oxygen on guanine base (8-oxoG)		NMS4 (X) (8-oxoG)
Removal of base (Abasic)		NMS4 (X) (Abasic)

Supplementary Table 4: Quantum Yield measurements showing the excitation wavelength (Ex), reference sample (Ref.), quantum yield of reference sample (Qr) and refractive index of reference sample (nr) and measured samples' quantum yield (Qs) and refractive index (ns) with confidence values (R^2).

Ex	Ref.	Qr	nr	R^2(Ref.)	Unknown	Qs	ns	R^2 (Unk.)
**550**	RDB - T1	0.7	1.33	0.999907305	ATTO 550 - T1	0.804132455	1.33	0.999764886
**550**	ATTO 550 - T3	0.8	1.33	0.999732951	ATTO 550 - T2	0.795370245	1.33	0.999556273
**550**	NMS1 - T2	0.76	1.4	0.995850854	ATTO 550 - T2	0.794693752	1.33	0.999556273
**550**	NMS2 - T2	0.77	1.4	0.998037073	ATTO 550 - T2	0.783686177	1.33	0.999556273
**550**	NMS3 - T2	0.76	1.4	0.998092052	ATTO 550 - T2	0.803539565	1.33	0.999556273
**550**	RDB - T2	0.7	1.33	0.991981701	ATTO 550 - T2	0.837038937	1.33	0.999556273
**550**	RDB - T3	0.7	1.33	0.999678604	ATTO 550 - T2	0.902796585	1.33	0.999556273
**550**	ATTO 550 - T2	0.8	1.33	0.999556273	ATTO 550 - T3	0.804656705	1.33	0.999732951
**550**	NMS1 - T2	0.76	1.4	0.995850854	ATTO 550 - T3	0.79931957	1.33	0.999732951
**550**	NMS2 - T2	0.77	1.4	0.998037073	ATTO 550 - T3	0.788247921	1.33	0.999732951
**550**	NMS3 - T2	0.76	1.4	0.998092052	ATTO 550 - T3	0.808216873	1.33	0.999732951
**550**	RDB - T2	0.7	1.33	0.991981701	ATTO 550 - T3	0.841911241	1.33	0.999732951
**550**	RDB - T3	0.7	1.33	0.999678604	ATTO 550 - T3	0.908051656	1.33	0.999732951
**550**	NMS2 - T1	0.77	1.4	0.999351269	NMS1 - T1	0.811843308	1.4	0.998396555
**550**	NMS3 - T1	0.76	1.4	0.999473103	NMS1 - T1	0.8197872	1.4	0.998396555
**550**	ATTO 550 - T2	0.8	1.33	0.999556273	NMS1 - T2	0.765074595	1.4	0.995850854
**550**	ATTO 550 - T3	0.8	1.33	0.999732951	NMS1 - T2	0.760646959	1.4	0.995850854
**550**	NMS2 - T2	0.77	1.4	0.998037073	NMS1 - T2	0.74947298	1.4	0.995850854
**550**	NMS3 - T2	0.76	1.4	0.998092052	NMS1 - T2	0.768459634	1.4	0.995850854
**550**	RDB - T2	0.7	1.33	0.991981701	NMS1 - T2	0.800496532	1.4	0.995850854
**550**	RDB - T3	0.7	1.33	0.999678604	NMS1 - T2	0.863383414	1.4	0.995850854
**550**	NMS2 - T3	0.77	1.4	0.998459128	NMS1 - T3	0.738493975	1.4	0.996338118
**550**	NMS3 - T3	0.76	1.4	0.999717452	NMS1 - T3	0.767643962	1.4	0.996338118
**550**	NMS1 - T1	0.76	1.4	0.998396555	NMS2 - T1	0.720828754	1.4	0.999351269
**550**	NMS3 - T1	0.76	1.4	0.999473103	NMS2 - T1	0.777534455	1.4	0.999351269
**550**	ATTO 550 - T2	0.8	1.33	0.999556273	NMS2 - T2	0.786028921	1.4	0.998037073
**550**	ATTO 550 - T3	0.8	1.33	0.999732951	NMS2 - T2	0.781480019	1.4	0.998037073
**550**	NMS1 - T2	0.76	1.4	0.995850854	NMS2 - T2	0.78081534	1.4	0.998037073
**550**	NMS3 - T2	0.76	1.4	0.998092052	NMS2 - T2	0.789506671	1.4	0.998037073
**550**	RDB - T2	0.7	1.33	0.991981701	NMS2 - T2	0.822421016	1.4	0.998037073
**550**	RDB - T3	0.7	1.33	0.999678604	NMS2 - T2	0.887030282	1.4	0.998037073
**550**	NMS1 - T3	0.76	1.4	0.996338118	NMS2 - T3	0.792423527	1.4	0.998459128
**550**	NMS3 - T3	0.76	1.4	0.999717452	NMS2 - T3	0.8003936	1.4	0.998459128
**550**	NMS1 - T1	0.76	1.4	0.998396555	NMS3 - T1	0.704573089	1.4	0.999473103
**550**	NMS2 - T1	0.77	1.4	0.999351269	NMS3 - T1	0.752635457	1.4	0.999473103
**550**	ATTO 550 - T2	0.8	1.33	0.999556273	NMS3 - T2	0.756652225	1.4	0.998092052
**550**	ATTO 550 - T3	0.8	1.33	0.999732951	NMS3 - T2	0.752273332	1.4	0.998092052
**550**	NMS1 - T2	0.76	1.4	0.995850854	NMS3 - T2	0.751633495	1.4	0.998092052
**550**	NMS2 - T2	0.77	1.4	0.998037073	NMS3 - T2	0.741222362	1.4	0.998092052
**550**	RDB - T2	0.7	1.33	0.991981701	NMS3 - T2	0.791684218	1.4	0.998092052
**550**	RDB - T3	0.7	1.33	0.999678604	NMS3 - T2	0.853878806	1.4	0.998092052
**550**	NMS1 - T3	0.76	1.4	0.996338118	NMS3 - T3	0.752432154	1.4	0.999717452
**550**	NMS2 - T3	0.77	1.4	0.998459128	NMS3 - T3	0.731140279	1.4	0.999717452
**550**	NMS1 - T1	0.76	1.4	0.998396555	Top -12 - T1	0.687232066	1.4	0.997708725
**550**	NMS2 - T1	0.77	1.4	0.999351269	Top -12 - T1	0.734111518	1.4	0.997708725
**550**	NMS3 - T1	0.76	1.4	0.999473103	Top -12 - T1	0.741294804	1.4	0.997708725
**550**	ATTO 550 - T2	0.8	1.33	0.999556273	Top -12 - T2	0.744499768	1.4	0.996475725
**550**	ATTO 550 - T3	0.8	1.33	0.999732951	Top -12 - T2	0.740191203	1.4	0.996475725
**550**	NMS1 - T2	0.76	1.4	0.995850854	Top -12 - T2	0.739561642	1.4	0.996475725
**550**	NMS2 - T2	0.77	1.4	0.998037073	Top -12 - T2	0.729317721	1.4	0.996475725
**550**	NMS3 - T2	0.76	1.4	0.998092052	Top -12 - T2	0.747793775	1.4	0.996475725
**550**	RDB - T2	0.7	1.33	0.991981701	Top -12 - T2	0.778969118	1.4	0.996475725
**550**	RDB - T3	0.7	1.33	0.999678604	Top -12 - T2	0.84016481	1.4	0.996475725
**550**	NMS1 - T3	0.76	1.4	0.996338118	Top -12 - T3	0.74359809	1.4	0.99386293

**550**	NMS2 - T3	0.77	1.4	0.998459128	Top -12 - T3	0.722556196	1.4	0.99386293
**550**	NMS3 - T3	0.76	1.4	0.999717452	Top -12 - T3	0.751077084	1.4	0.99386293
**550**	NMS1 - T1	0.76	1.4	0.998396555	Top -18 - T1	0.707095204	1.4	0.999083843
**550**	NMS2 - T1	0.77	1.4	0.999351269	Top -18 - T1	0.755329618	1.4	0.999083843
**550**	NMS3 - T1	0.76	1.4	0.999473103	Top -18 - T1	0.762720523	1.4	0.999083843
**550**	ATTO 550 - T2	0.8	1.33	0.999556273	Top -18 - T2	0.728682996	1.4	0.999414902
**550**	ATTO 550 - T3	0.8	1.33	0.999732951	Top -18 - T2	0.724465966	1.4	0.999414902
**550**	NMS1 - T2	0.76	1.4	0.995850854	Top -18 - T2	0.72384978	1.4	0.999414902
**550**	NMS2 - T2	0.77	1.4	0.998037073	Top -18 - T2	0.713823489	1.4	0.999414902
**550**	NMS3 - T2	0.76	1.4	0.998092052	Top -18 - T2	0.731907022	1.4	0.999414902
**550**	RDB - T2	0.7	1.33	0.991981701	Top -18 - T2	0.762420051	1.4	0.999414902
**550**	RDB - T3	0.7	1.33	0.999678604	Top -18 - T2	0.82231565	1.4	0.999414902
**550**	NMS1 - T3	0.76	1.4	0.996338118	Top -18 - T3	0.730820344	1.4	0.998974109
**550**	NMS2 - T3	0.77	1.4	0.998459128	Top -18 - T3	0.710140027	1.4	0.998974109
**550**	NMS3 - T3	0.76	1.4	0.999717452	Top -18 - T3	0.738170821	1.4	0.998974109
**550**	NMS1 - T1	0.76	1.4	0.998396555	Top -7 - T1	0.691377728	1.4	0.999062676
**550**	NMS2 - T1	0.77	1.4	0.999351269	Top -7 - T1	0.738539977	1.4	0.999062676
**550**	NMS3 - T1	0.76	1.4	0.999473103	Top -7 - T1	0.745766595	1.4	0.999062676
**550**	ATTO 550 - T2	0.8	1.33	0.999556273	Top -7 - T2	0.723404389	1.4	0.998304516
**550**	ATTO 550 - T3	0.8	1.33	0.999732951	Top -7 - T2	0.719217907	1.4	0.998304516
**550**	NMS1 - T2	0.76	1.4	0.995850854	Top -7 - T2	0.718606185	1.4	0.998304516
**550**	NMS2 - T2	0.77	1.4	0.998037073	Top -7 - T2	0.708652525	1.4	0.998304516
**550**	NMS3 - T2	0.76	1.4	0.998092052	Top -7 - T2	0.72660506	1.4	0.998304516
**550**	RDB - T2	0.7	1.33	0.991981701	Top -7 - T2	0.756897051	1.4	0.998304516
**550**	RDB - T3	0.7	1.33	0.999678604	Top -7 - T2	0.816358765	1.4	0.998304516
**550**	NMS1 - T3	0.76	1.4	0.996338118	Top -7 - T3	0.733526535	1.4	0.999315897
**550**	NMS2 - T3	0.77	1.4	0.998459128	Top -7 - T3	0.71276964	1.4	0.999315897
**550**	NMS3 - T3	0.76	1.4	0.999717452	Top -7 - T3	0.740904231	1.4	0.999315897

Supplementary Table 5: Quantum Yield measurements showing the excitation wavelength (Ex), reference sample (Ref.), quantum yield of reference sample (Qr) and refractive index of reference sample (nr) and measured samples' quantum yield (Qs) and refractive index (ns) with confidence values (R^2).

Ex	Ref.	Qr	Nr	R^2 (Ref.)	Unk.	Qs	Ns	R^2 (Unk.)
**646**	ATTO 647N - T1	0.65	1.33	0.999999666	ATTO 647N - T2	0.637195275	1.33	0.99884106
**646**	ATTO 647N - T1	0.65	1.33	0.999999666	ATTO 647N - T3	0.658075988	1.33	0.99959802
**646**	ATTO 647N - T1	0.65	1.33	0.999999666	Bottom +11 - T1	0.539973545	1.4	0.999931515
**646**	ATTO 647N - T1	0.65	1.33	0.996215287	Bottom +11 - T3	0.50486727	1.4	0.999231646
**646**	ATTO 647N - T1	0.65	1.33	0.999999666	Bottom +6 - T1	0.552265222	1.4	0.999339418
**646**	ATTO 647N - T1	0.65	1.33	0.996215287	Bottom +6 - T3	0.492425515	1.4	0.997963665
**646**	ATTO 647N - T1	0.65	1.33	0.999999666	Bottom +9 - T1	0.528427847	1.4	0.998569767
**646**	ATTO 647N - T1	0.65	1.33	0.996215287	Bottom +9 - T3	0.513297417	1.4	0.999291052
**646**	ATTO 647N - T1	0.65	1.33	0.999999666	NMS4 - T1	0.57295812	1.4	0.999913668
**646**	ATTO 647N - T1	0.65	1.33	0.996215287	NMS4 - T3	0.538354994	1.4	0.99878214
**646**	ATTO 647N - T2	0.65	1.33	0.99884106	ATTO 647N - T1	0.663062042	1.33	0.999999666
**646**	ATTO 647N - T2	0.65	1.33	0.99884106	ATTO 647N - T3	0.67130032	1.33	0.99959802
**646**	ATTO 647N - T2	0.65	1.33	0.999421881	Bottom +11 - T2	0.532648855	1.4	0.999886686
**646**	ATTO 647N - T2	0.65	1.33	0.99884106	Bottom +11 - T1	0.550824556	1.4	0.999931515
**646**	ATTO 647N - T2	0.65	1.33	0.99884106	Bottom +6 - T1	0.56336324	1.4	0.999339418
**646**	ATTO 647N - T2	0.65	1.33	0.999421881	Bottom +6 - T2	0.541131398	1.4	0.999553447
**646**	ATTO 647N - T2	0.65	1.33	0.999421881	Bottom +9 - T2	0.530704257	1.4	0.99903735
**646**	ATTO 647N - T2	0.65	1.33	0.99884106	Bottom +9 - T1	0.539046842	1.4	0.998569767
**646**	ATTO 647N - T2	0.65	1.33	0.999421881	NMS4 - T2	0.556320655	1.4	0.999484075
**646**	ATTO 647N - T2	0.65	1.33	0.99884106	NMS4 - T1	0.58447197	1.4	0.999913668
**646**	ATTO 647N - T3	0.65	1.33	0.99959802	ATTO 647N - T1	0.642023121	1.33	0.999999666
**646**	ATTO 647N - T3	0.65	1.33	0.99959802	ATTO 647N - T2	0.629375538	1.33	0.99884106
**646**	ATTO 647N - T3	0.65	1.33	0.99959802	Bottom +11 - T1	0.533346925	1.4	0.999931515
**646**	ATTO 647N - T3	0.65	1.33	0.99959802	Bottom +6 - T1	0.545487757	1.4	0.999339418
**646**	ATTO 647N - T3	0.65	1.33	0.99959802	Bottom +9 - T1	0.521942917	1.4	0.998569767
**646**	ATTO 647N - T3	0.65	1.33	0.99959802	NMS4 - T1	0.565926708	1.4	0.999913668

Supplementary Table 6: Quantum Yield values compared to literature values.

Construct	Qs	Literature value
**ATTO 550**	0.82±0.04	0.8
**NMS 1**	0.78±0.04	0.76±0.015
**NMS 2**	0.79±0.04	0.77±0.015
**NMS 3**	0.76±0.04	0.76±0.015
**Top -7**	0.73±0.03	-
**Top -12**	0.75±0.03	-
**Top -18**	0.74±0.03	-
**ATTO 647N**	0.65±0.02	0.65
**NMS 4**	0.57±0.02	-
**Bottom +6**	0.54±0.03	-
**Bottom +9**	0.53±0.01	-
**Bottom +11**	0.53±0.02	-

Supplementary Table 7: Table showing Epsilon, quantum yield of donor (Qd), refractive index (n), Kappa squared (K^2), J Integral (J) and Forster radius (R_0).

**Donor**	**Acceptor**	**Epsilon**	**Qd**	**n**	**K^2**	**J**	**R_0**
ATTO 550	ATTO 647N	150000	0.82	1.33	0.66666667	3.70E+15	62.0
NMS1	NMS4	150000	0.78	1.4	0.66666667	4.11E+15	60.4
NMS2	NMS4	150000	0.79	1.4	0.66666667	4.09E+15	60.5
NMS3	NMS4	150000	0.76	1.4	0.66666667	4.11E+15	60.2
Top -7	Bottom +6	150000	0.73	1.4	0.66666667	4.15E+15	59.9
Top -7	Bottom +9	150000	0.73	1.4	0.66666667	3.95E+15	59.4
Top -7	Bottom +11	150000	0.73	1.4	0.66666667	4.45E+15	60.6
Top -12	Bottom +6	150000	0.75	1.4	0.66666667	4.14E+15	60.1
Top -12	Bottom +9	150000	0.75	1.4	0.66666667	3.94E+15	59.6
Top -12	Bottom +11	150000	0.75	1.4	0.66666667	4.44E+15	60.8
Top -18	Bottom +6	150000	0.74	1.4	0.66666667	4.09E+15	59.9
Top -18	Bottom +9	150000	0.74	1.4	0.66666667	3.89E+15	59.4
Top -18	Bottom +11	150000	0.74	1.4	0.66666667	4.39E+15	60.6

Supplementary Table 8: Dimensions of the linkers with different dyes.

	Linker Length [Å]	Linker Width [Å]	R_1 [Å]	R_2 [Å]	R_3 [Å]
dT-C6-ATTO 550	16.43	5.9	8.6	5.37	4.62
dT-C6-ATTO 647N	15.02	4.49	8.62	5.45	5.02

Supplementary Table 9: Predicted FRET efficiencies and distances for control duplex with different labelling positions.

	Top -7 Bottom +6	Top -7 Bottom +9	Top -7 Bottom +11	Top -12 Bottom +6	Top -12 Bottom +9	Top -12 Bottom +11	Top -18 Bottom +6	Top -18 Bottom +9	Top -18 Bottom +11
Predicted FRET efficiencies	0.96	0.81	0.65	0.65	0.52	0.45	0.39	0.19	0.12
Predicted Mean Distance [Å]	35.13	47.45	56.12	56.12	56.12	64.84	68.20	80.60	87.36

Supplementary Table 10: Differences in predicted and measured mean FRET efficiencies.

	Top -7 Bottom +6	Top -7 Bottom +9	Top -7 Bottom +11	Top -12 Bottom +6	Top -12 Bottom +9	Top -12 Bottom +11	Top -18 Bottom +6	Top -18 Bottom +9	Top -18 Bottom +11
Förster radius	59.9	59.4	60.6	60.1	59.6	60.8	59.9	59.4	60.6
Predicted Mean FRET efficiencies	0.95	0.77	0.61	0.60	0.45	0.41	0.33	0.15	0.11
Measured Mean FRET efficiencies	0.60	0.49	0.39	0.38	0.27	0.25	0.22	0.16	0.14
% Difference	45.2	44.4	44.0	44.9	50.0	48.5	40	6.5	24

Supplementary Table 11: Lifetime values with standard deviation.

	**Lifetime (ns)**	**Sd (ns)**
ATTO 550	3.60	0.0033
NMS 1	3.73	0.0088
NMS 2	3.82	0
NMS 3	3.73	0.0033
T-7	3.90	0
T-12	3.81	0.0058
T-18	3.59	0.0058
NMS 1+4	3.15	0.01
NMS 2+4	2.59	0.0333
NMS 3+4	2.63	0.04
T-7 B+6	2.08	0.023
T-7 B+9	2.45	0.012
T-7 B+11	2.59	0.012
T-12 B+6	2.40	0.017
T-12 B+9	2.88	0.006
T-12 B+11	2.90	0.012
T-18 B+6	2.86	0
T-18 B+9	3.18	0
T-18 B+11	3.25	0.006
ATTO 647N	3.58	0.0033
NMS 4	4.08	0.0033
B+6	4.06	0.006
B+9	4.24	0.0033
B+11	4.08	0.006

Supplementary Table 12: Residual anisotropy results.

	Residual			Residual
ATTO 550 T1	0.007		ATTO 647N T1	0.019
ATTO 550 T2	0.002		ATTO 647N T2	0.006
ATTO 550 T3	0.017		ATTO 647N T3	0.007
Average	0.009		Average	0.011
NMS 1 T1	0.085		NMS 4 T1	0.018
NMS 1 T2	0.052		NMS 4 T2	0.035
NMS 1 T3	0.081		NMS 4 T3	0.027
Average	0.073		Average	0.027
NMS 2 T1	0.091		Bottom +6 T1	0.023
NMS 2 T2	0.111		Bottom +6 T2	0.043
NMS 2 T3	0.094		Bottom +6 T3	0.033
Average	0.099		Average	0.033
NMS 3 T1	0.074		Bottom +9 T1	0.027
NMS 3 T2	0.091		Bottom +9 T2	0.028
NMS 3 T3	0.087		Bottom +9 T3	0.027
Average	0.084		Average	0.027
T-7 T1	0.144			
T-7 T2	0.132		Bottom +11 T1	0.021
T-7 T3	0.138		Bottom +11 T2	0.016
Average	0.138		Bottom +11 T3	0.026
			Average	0.021
T-12 T1	0.101			
T-12 T2	0.100			
T-12 T3	0.105			
Average	0.102			
T-18 T1	0.064			
T-18 T2	0.042			
T-18 T3	0.054			
Average	0.053			

**Construct**	**Residual Anisotropy**
ATTO 550	0.009 ± 0.0076
NMS 1	0.073 ± 0.018
NMS 2	0.099 ± 0.0108
NMS 3	0.084 ± 0.0089
T-7	0.138 ± 0.006
T-12	0.102 ± 0.0026
T-18	0.053 ± 0.011
ATTO 647N	0.011 ± 0.007
NMS 4	0.027 ± 0.009
Bottom +6	0.033 ± 0.010
Bottom +9	0.027 ± 0.001
Bottom +11	0.021 ± 0.005

Supplementary Table 13: Summary results.

	Top -7	Bottom +6	Top -7	Bottom +9	Top -7	Bottom +11	Top -12	Bottom +6
Dye Position	T24 (C6), T Strand	T19 (C6), B Strand	T24 (C6), T Strand	T16 (C6), B Strand	T24 (C6), T Strand	T14 (C6), B Strand	T19 (C6), T Strand	T19 (C6), B Strand
Residual anisotropy	0.138 ± 0.006	0.033 ± 0.010	0.138 ± 0.006	0.027 ± 0.001	0.138 ± 0.006	0.021 ± 0.005	0.102 ± 0.0026	0.033 ± 0.010
Combined anisotropy	0.068±0.015		0.061±0.006		0.054±0.015		0.058±0.011	
Lifetime	3.9±0	4.06±0.006	3.9±0	4.24±0.0033	3.9±0	4.08±0.006	3.81±0.0058	4.06±0.006
Fluorescence quantum yield	0.73±0.03	0.54±0.03	0.73±0.03	0.53±0.01	0.73±0.03	0.53±0.02	0.75±0.03	0.54±0.03

Top -12	Bottom +9	Top -12	Bottom +11	Top -18	Bottom +6	Top -18	Bottom +9	Top -18	Bottom +11
T19 (C6), T Strand	T16 (C6), B Strand	T19 (C6), T Strand	T14 (C6), B Strand	T13 (C6), T Strand	T19 (C6), B Strand	T13 (C6), T Strand	T16 (C6), B Strand	T13 (C6), T Strand	T14 (C6), B Strand
0.102 ± 0.0026	0.027 ± 0.001	0.102 ± 0.0026	0.021 ± 0.005	0.053 ± 0.011	0.033 ± 0.010	0.053 ± 0.011	0.027 ± 0.001	0.053 ± 0.011	0.021 ± 0.005
0.053±0.009		0.046±0.014		0.039±0.008		0.027±0.005		0.026±0.008	
3.81±0.0058	4.24±0.0033	3.81±0.0058	4.08±0.006	3.59±0.0058	4.06±0.006	3.59±0.0058	4.24±0.0033	3.59±0.0058	4.08±0.006
0.75±0.03	0.53±0.01	0.75±0.03	0.53±0.02	0.74±0.03	0.54±0.03	0.74±0.03	0.53±0.01	0.74±0.03	0.53±0.02

	NMS 1	NMS 4	NMS 2	NMS 4	NMS 3	NMS 4
Dye Position	T31 (C6), D-strand	T31 (C6), A-strand	T23 (C6), D-strand	T31 (C6), A-strand	T19 (C6), D-strand	T31 (C6), A-strand
Residual anisotropy	0.073 ± 0.018	0.027 ± 0.009	0.099 ± 0.0108	0.027 ± 0.009	0.084 ± 0.0089	0.027 ± 0.009
Combined anisotropy	0.044±0.013		0.052±0.011		0.048±0.010	
Lifetime	3.73±0.0088	4.08±0.0033	3.82±0	4.08±0.0033	3.73±0.0033	4.08±0.0033
Fluorescence quantum yield	0.78±0.04	0.57±0.02	0.79±0.04	0.57±0.02	0.76±0.04	0.57±0.02

Supplementary Table 14: Estimated distances for each labelling position and damage type and distance changes in AV mean position.

TX	BX	Construct	R (Å)
T7	B6	Control	57.21
T7	B6	O6-Me	54.84
T7	B6	Ribo	55.66
T7	B6	8-oxoG	56.62
T7	B6	abasic	54.00
T7	B6	Nick	50.68
T7	B6	Gap	48.94
T7	B9	Control	60.80
T7	B9	O6-Me	60.00
T7	B9	Ribo	60.96
T7	B9	8-oxoG	61.83
T7	B9	abasic	60.12
T7	B9	Nick	56.92
T7	B9	Gap	54.45
T7	B11	Control	64.39
T7	B11	O6-Me	65.29
T7	B11	Ribo	65.07
T7	B11	8-oxoG	64.68
T7	B11	abasic	62.58
T7	B11	Nick	60.62
T7	B11	Gap	55.31
T12	B6	Control	64.84
T12	B6	O6-Me	65.09
T12	B6	Ribo	64.72
T12	B6	8-oxoG	65.62
T12	B6	abasic	62.88
T12	B6	Nick	59.46
T12	B6	Gap	58.36
T12	B9	Control	69.97
T12	B9	O6-Me	70.68
T12	B9	Ribo	70.41
T12	B9	8-oxoG	70.93
T12	B9	abasic	68.64
T12	B9	Nick	64.60
T12	B9	Gap	62.62
T12	B11	Control	70.87
T12	B11	O6-Me	73.42
T12	B11	Ribo	70.25
T12	B11	8-oxoG	70.72
T12	B11	abasic	69.72
T12	B11	Nick	64.67
T12	B11	Gap	63.15
T18	B6	Control	73.48
T18	B6	O6-Me	72.60
T18	B6	Ribo	73.59
T18	B6	8-oxoG	73.20
T18	B6	abasic	69.08
T18	B6	Nick	69.04
T18	B6	Gap	68.33
T18	B9	Control	77.55

T18	B9	O6-Me	76.19
T18	B9	Ribo	76.22
T18	B9	8-oxoG	77.54
T18	B9	abasic	75.95
T18	B9	Nick	71.15
T18	B9	Gap	71.00
T18	B11	Control	79.47
T18	B11	O6-Me	81.58
T18	B11	Ribo	79.10
T18	B11	8-oxoG	80.33
T18	B11	abasic	78.12
T18	B11	Nick	73.11
T18	B11	Gap	71.47

**Construct**	**T7 (Å)**	**B6 (Å)**
Ribo	4.264	1.517
8-oxoG	3.350	0.666
Abasic	2.707	2.477
Nick	5.208	2.611
Gap	3.598	2.306

**Construct**	**T12 (Å)**	**B9 (Å)**
Ribo	2.044	1.814
8-oxoG	2.346	1.238
Abasic	1.284	0.682
Nick	2.965	2.902
Gap	3.483	3.397

**Construct**	**T18 (Å)**	**B11 (Å)**
Ribo	3.566	1.233
8-oxoG	3.401	1.031
Abasic	2.958	1.073
Nick	5.587	3.356
Gap	4.305	5.124

**Construct**	**Mean Difference:**	**Mean Relative Difference:**	**Standard Deviation of Differences:**
	**(Å)**		
**Ribo**	1.44E-05	2.52E-09	5.46E-06
**8-oxoG**	2.44E-06	4.66E-08	1.52E-06
**Abasic**	1.56E-05	2.66E-07	9.40E-06
**Nick**	6.84E-06	1.08E-07	5.53E-06
**Gap**	5.67E-06	1.21E-07	3.61E-06

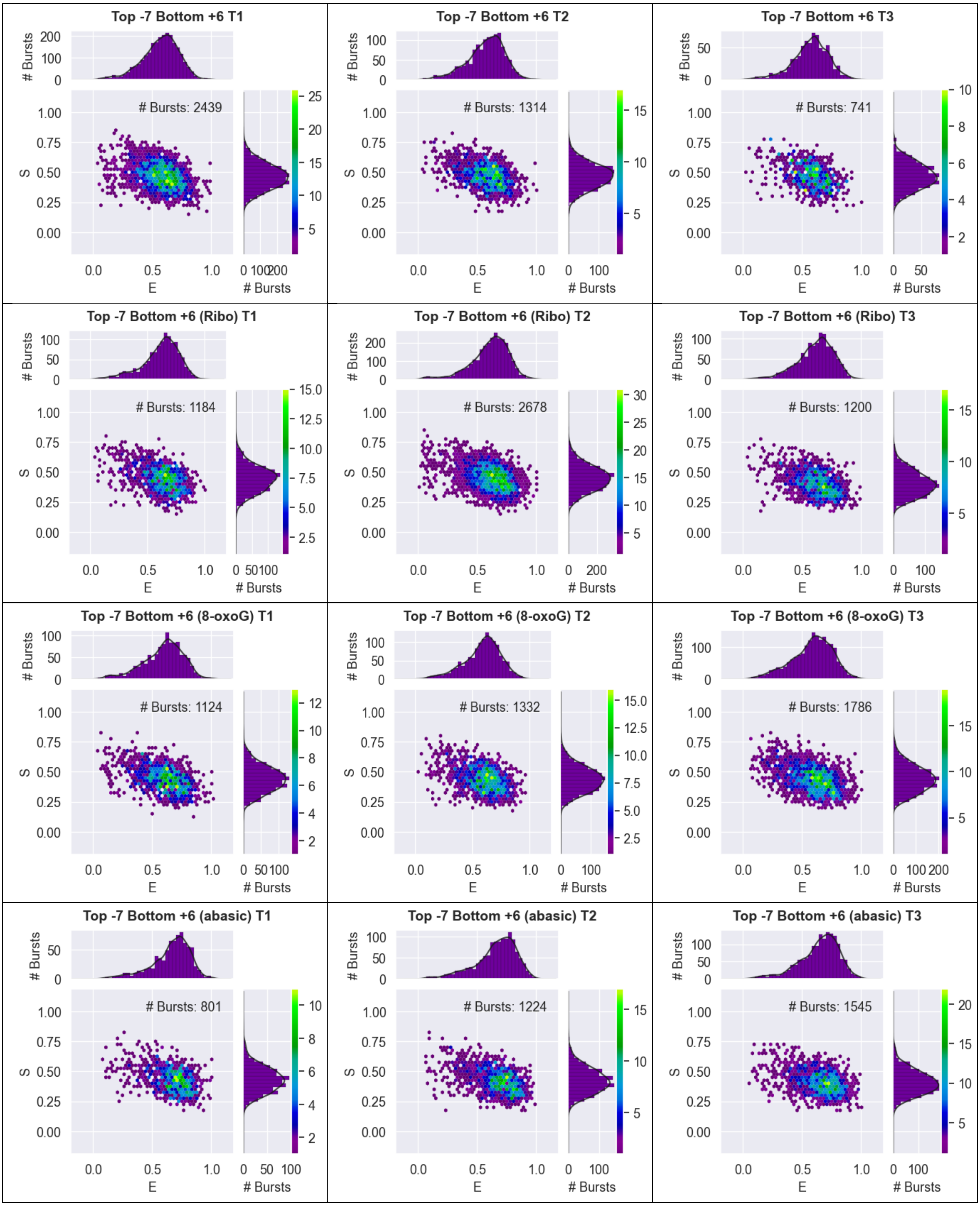
Top -7 Bottom +6 T1
Bursts: 2439
Top -7 Bottom +6 T2
Bursts: 1314
Top -7 Bottom +6 T3
Bursts: 741
Top -7 Bottom +6 (Ribo) T1
Bursts: 1184
Top -7 Bottom +6 (Ribo) T2
Bursts: 2678
Top -7 Bottom +6 (Ribo) T3
Bursts: 1200
Top -7 Bottom +6 (8-oxoG) T1
Bursts: 1124
Top -7 Bottom +6 (8-oxoG) T2
Bursts: 1332
Top -7 Bottom +6 (8-oxoG) T3
Bursts: 1786
Top -7 Bottom +6 (abasic) T1
Bursts: 801
Top -7 Bottom +6 (abasic) T2
Bursts: 1224
Top -7 Bottom +6 (abasic) T3
Bursts: 1545
E
S
Bursts

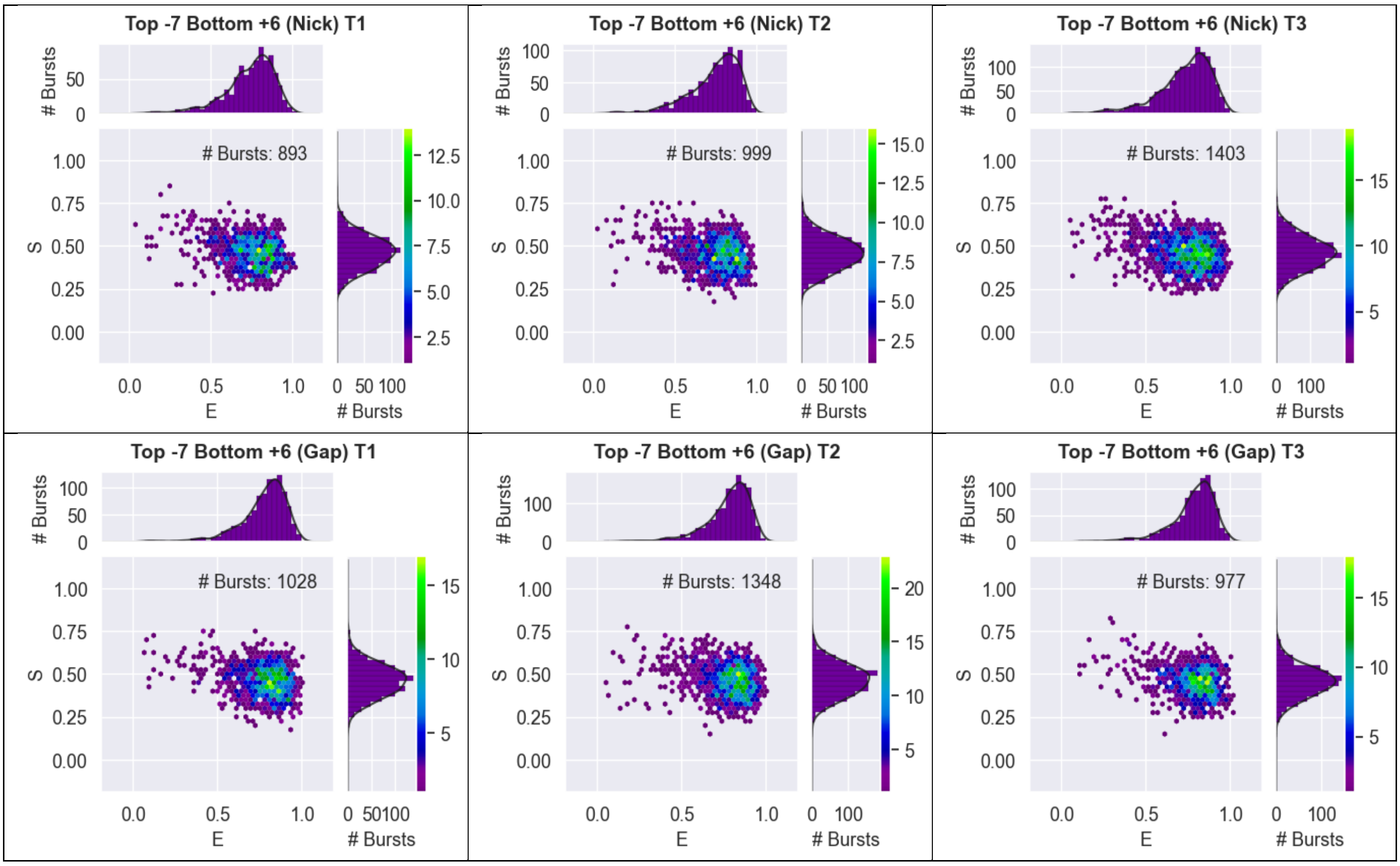

Supplementary Figure 1: 2D ES histograms from DCBS analysis showing FRET efficiency (E) and stoichiometry (S) for each independent repeat of the seven duplex constructs for the dye-pair Top -7 Bottom +6.

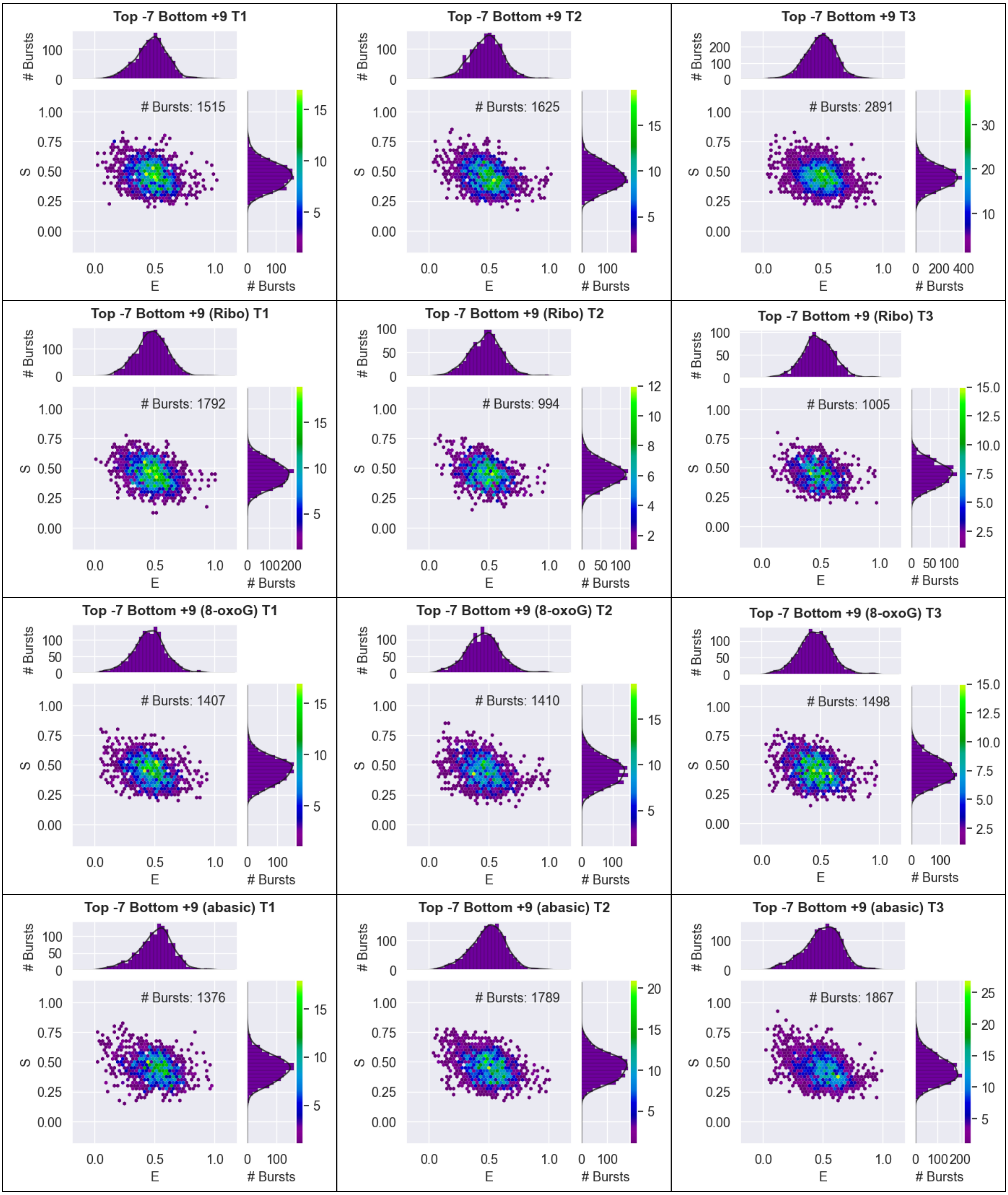
Top -7 Bottom +9 T1
Bursts: 1515
Top -7 Bottom +9 T2
Bursts: 1625
Top -7 Bottom +9 T3
Bursts: 2891
Top -7 Bottom +9 (Ribo) T1
Bursts: 1792
Top -7 Bottom +9 (Ribo) T2
Bursts: 994
Top -7 Bottom +9 (Ribo) T3
Bursts: 1005
Top -7 Bottom +9 (8-oxoG) T1
Bursts: 1407
Top -7 Bottom +9 (8-oxoG) T2
Bursts: 1410
Top -7 Bottom +9 (8-oxoG) T3
Bursts: 1498
Top -7 Bottom +9 (abasic) T1
Bursts: 1376
Top -7 Bottom +9 (abasic) T2
Bursts: 1789
Top -7 Bottom +9 (abasic) T3
Bursts: 1867
E
S
Bursts

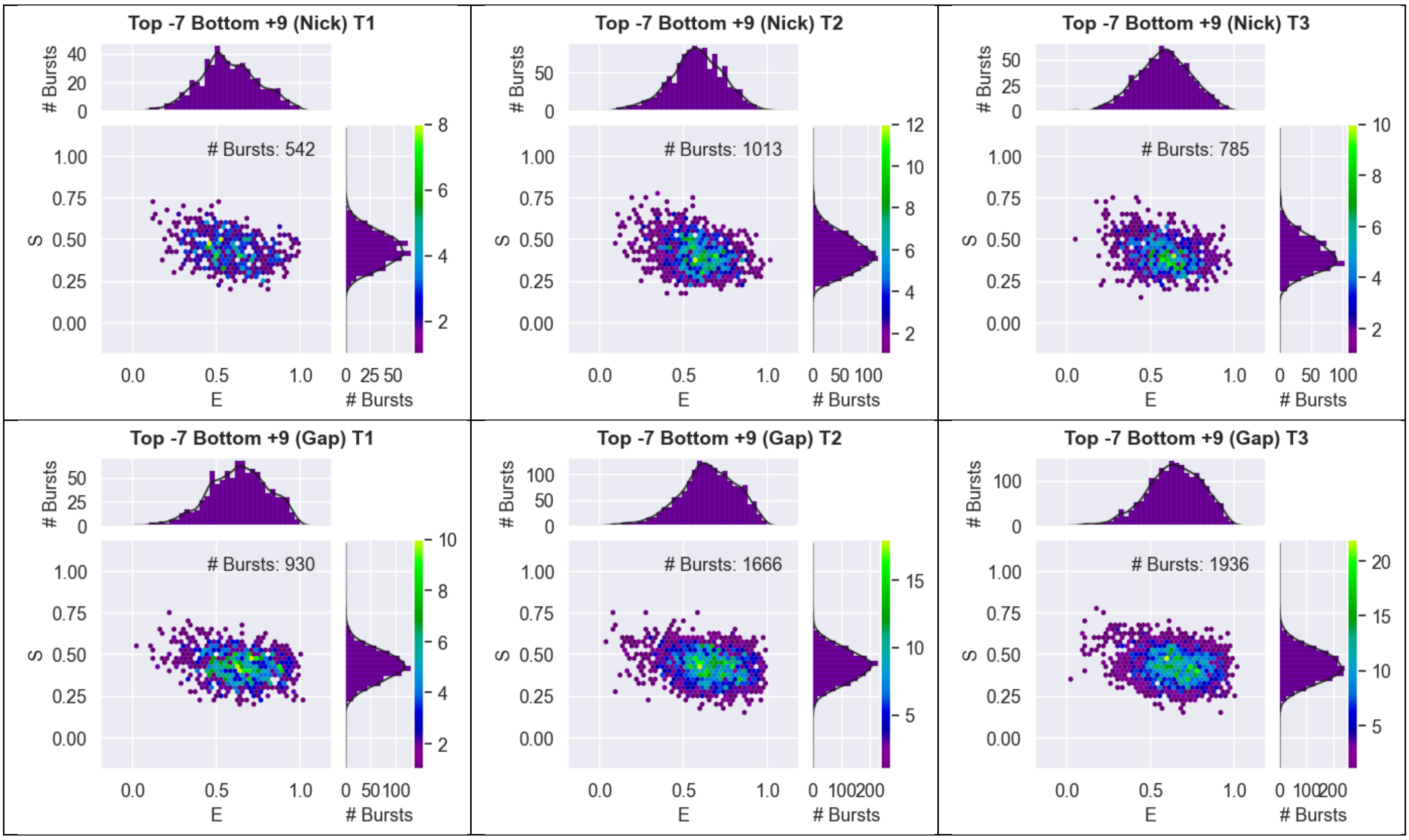

Supplementary Figure 2: 2D ES histograms from DCBS analysis showing FRET efficiency (E) and stoichiometry (S) for each independent repeat of the seven duplex constructs for the dye-pair Top -7 Bottom +9.

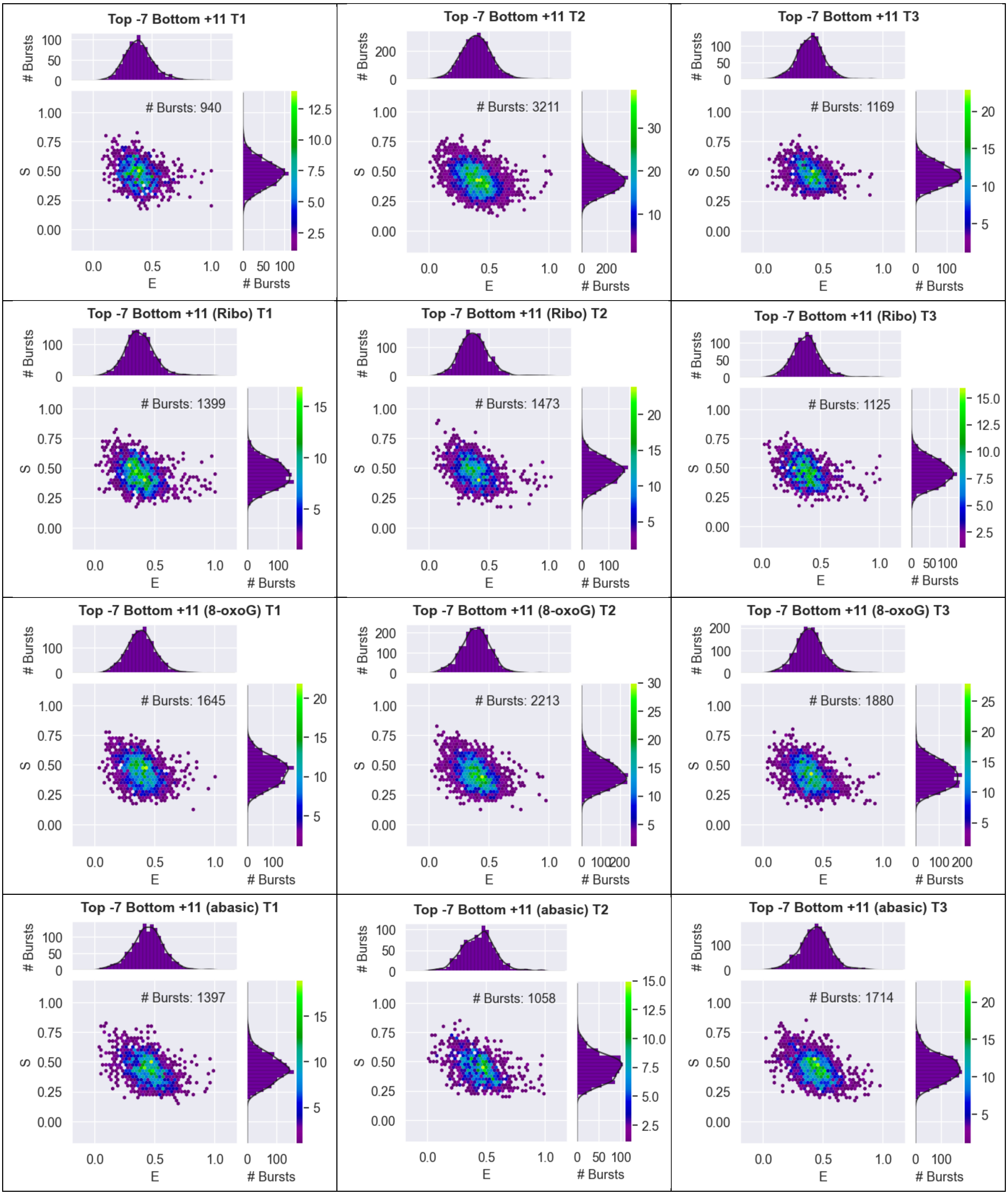

Top -7 Bottom +11 T1
Bursts: 940
Top -7 Bottom +11 T2
Bursts: 3211
Top -7 Bottom +11 T3
Bursts: 1169
Top -7 Bottom +11 (Ribo) T1
Bursts: 1399
Top -7 Bottom +11 (Ribo) T2
Bursts: 1473
Top -7 Bottom +11 (Ribo) T3
Bursts: 1125
Top -7 Bottom +11 (8-oxoG) T1
Bursts: 1645
Top -7 Bottom +11 (8-oxoG) T2
Bursts: 2213
Top -7 Bottom +11 (8-oxoG) T3
Bursts: 1880
Top -7 Bottom +11 (abasic) T1
Bursts: 1397
Top -7 Bottom +11 (abasic) T2
Bursts: 1058
Top -7 Bottom +11 (abasic) T3
Bursts: 1714
S
E
Bursts

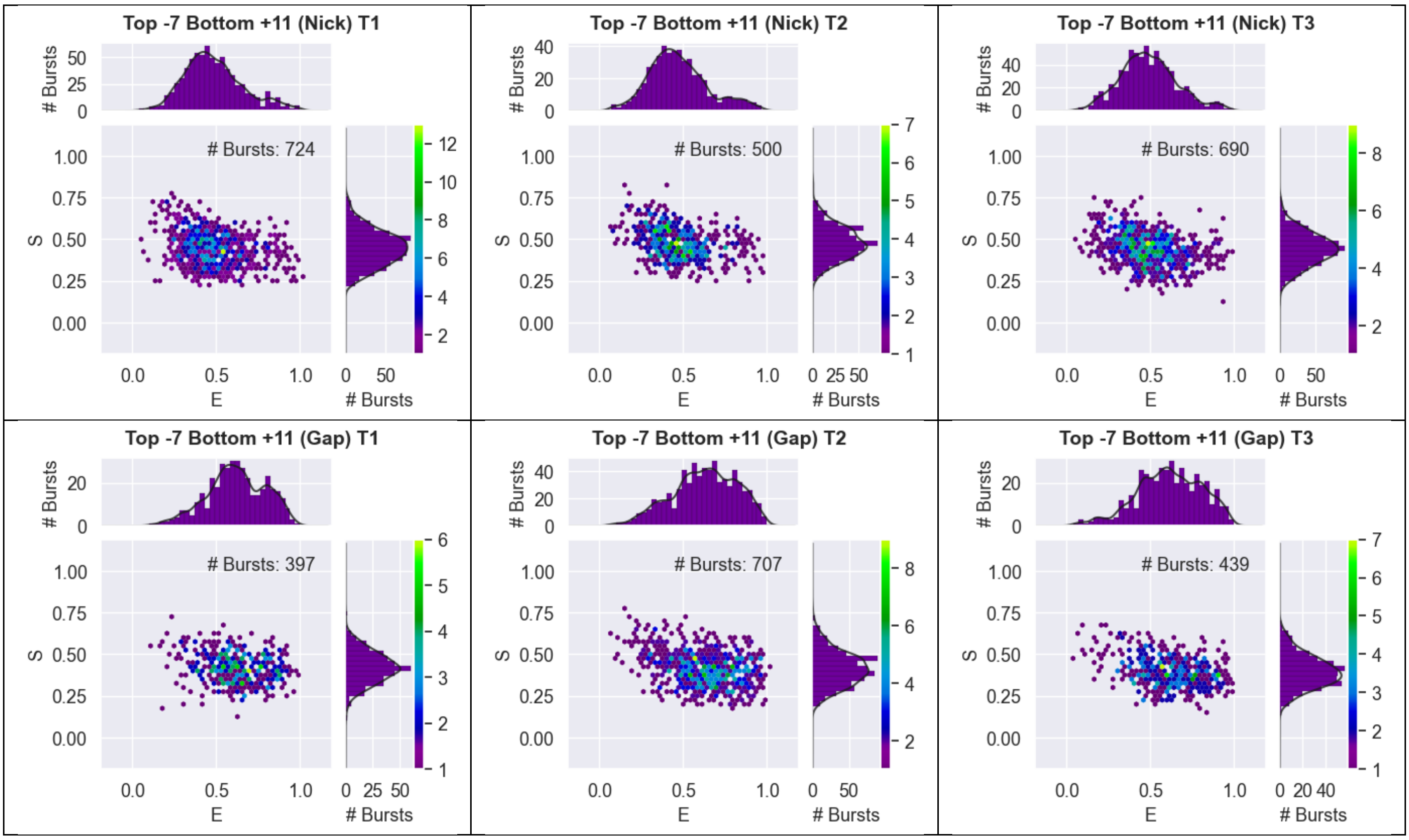

Supplementary Figure 3: 2D ES histograms from DCBS analysis showing FRET efficiency (E) and stoichiometry (S) for each independent repeat of the seven duplex constructs for the dye-pair Top -7 Bottom +11.

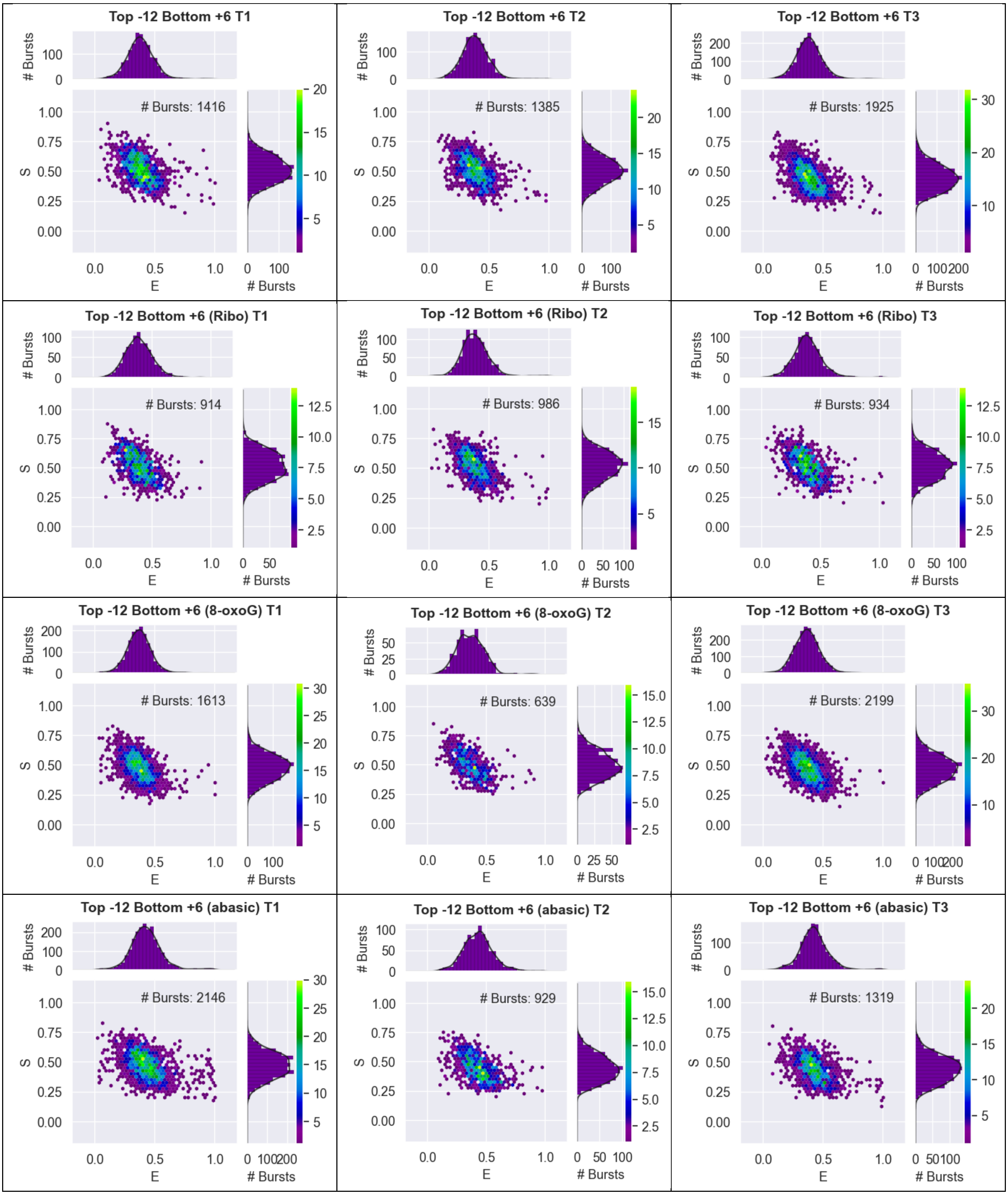
Top -12 Bottom +6 T1
Bursts: 1416
Top -12 Bottom +6 T2
Bursts: 1385
Top -12 Bottom +6 T3
Bursts: 1925
Top -12 Bottom +6 (Ribo) T1
Bursts: 914
Top -12 Bottom +6 (Ribo) T2
Bursts: 986
Top -12 Bottom +6 (Ribo) T3
Bursts: 934
Top -12 Bottom +6 (8-oxoG) T1
Bursts: 1613
Top -12 Bottom +6 (8-oxoG) T2
Bursts: 639
Top -12 Bottom +6 (8-oxoG) T3
Bursts: 2199
Top -12 Bottom +6 (abasic) T1
Bursts: 2146
Top -12 Bottom +6 (abasic) T2
Bursts: 929
Top -12 Bottom +6 (abasic) T3
Bursts: 1319
S
E
Bursts

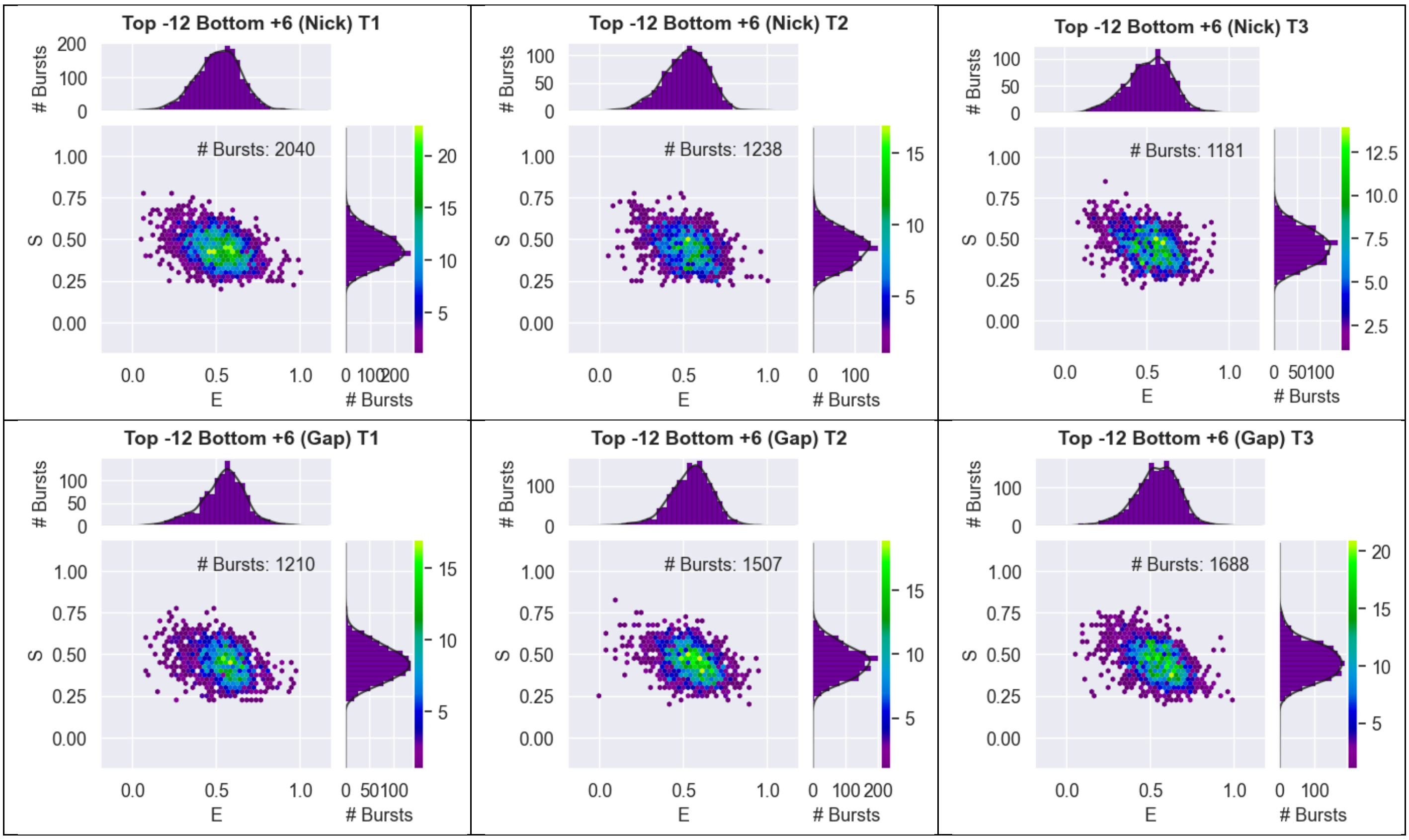

Supplementary Figure 4: 2D ES histograms from DCBS analysis showing FRET efficiency (E) and stoichiometry (S) for each independent repeat of the seven duplex constructs for the dye-pair Top -12 Bottom +6.

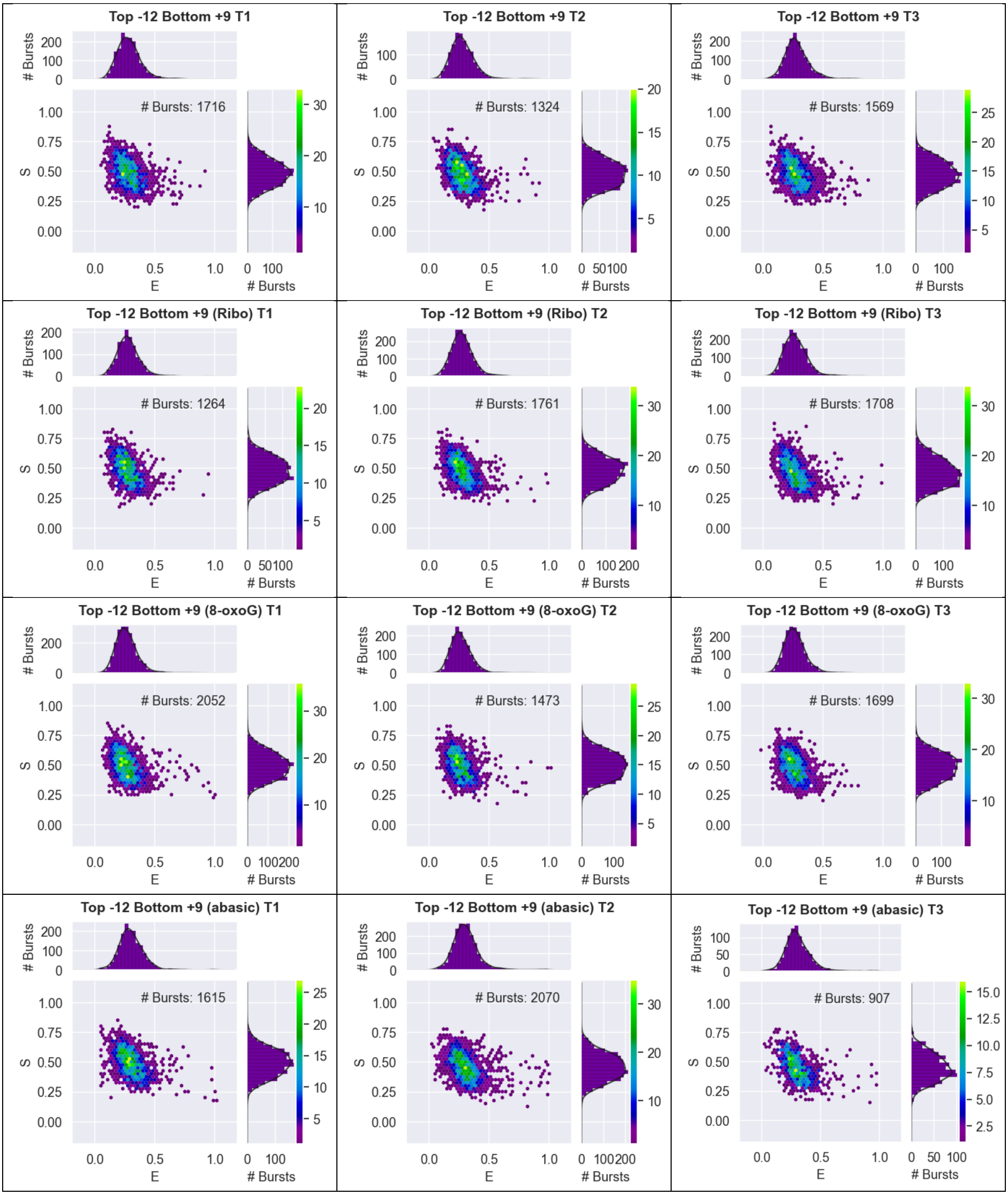
Top -12 Bottom +9 T1
Bursts: 1716
Top -12 Bottom +9 T2
Bursts: 1324
Top -12 Bottom +9 T3
Bursts: 1569
Top -12 Bottom +9 (Ribo) T1
Bursts: 1264
Top -12 Bottom +9 (Ribo) T2
Bursts: 1761
Top -12 Bottom +9 (Ribo) T3
Bursts: 1708
Top -12 Bottom +9 (8-oxoG) T1
Bursts: 2052
Top -12 Bottom +9 (8-oxoG) T2
Bursts: 1473
Top -12 Bottom +9 (8-oxoG) T3
Bursts: 1699
Top -12 Bottom +9 (abasic) T1
Bursts: 1615
Top -12 Bottom +9 (abasic) T2
Bursts: 2070
Top -12 Bottom +9 (abasic) T3
Bursts: 907
S
E
Bursts

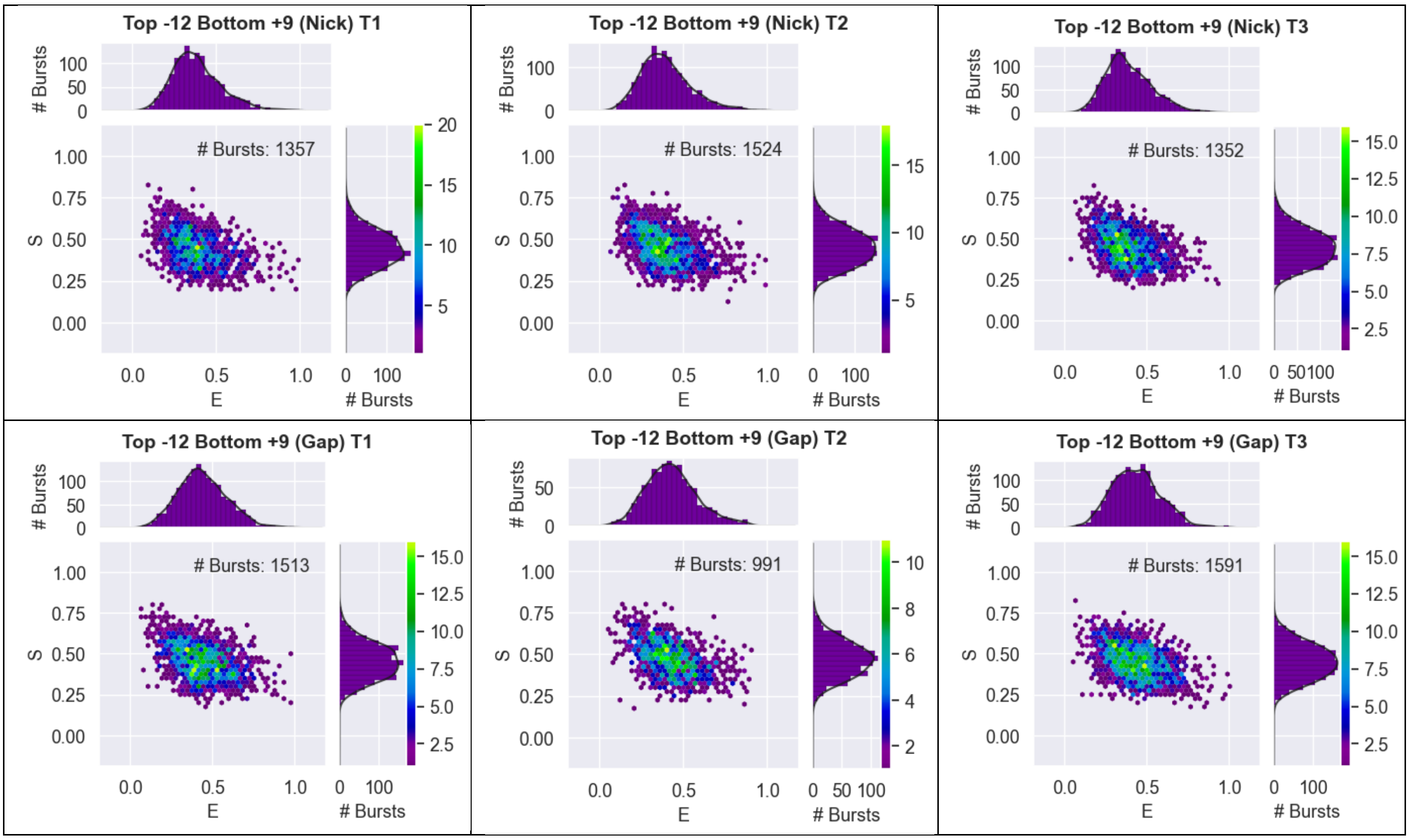

Supplementary Figure 5: 2D ES histograms from DCBS analysis showing FRET efficiency (E) and stoichiometry (S) for each independent repeat of the seven duplex constructs for the dye-pair Top -12 Bottom +9.

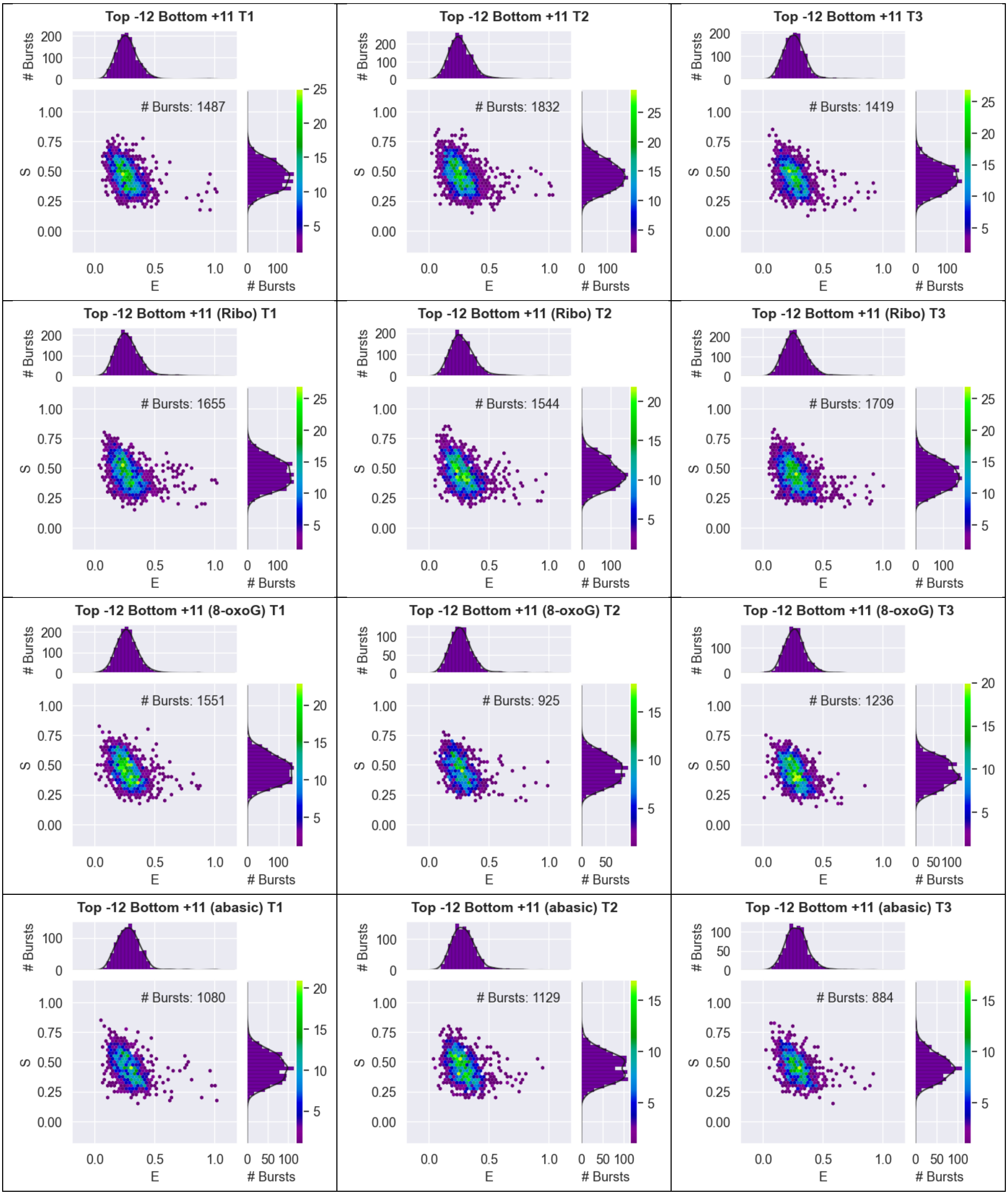
Top -12 Bottom +11 T1
Bursts: 1487
Top -12 Bottom +11 T2
Bursts: 1832
Top -12 Bottom +11 T3
Bursts: 1419
Top -12 Bottom +11 (Ribo) T1
Bursts: 1655
Top -12 Bottom +11 (Ribo) T2
Bursts: 1544
Top -12 Bottom +11 (Ribo) T3
Bursts: 1709
Top -12 Bottom +11 (8-oxoG) T1
Bursts: 1551
Top -12 Bottom +11 (8-oxoG) T2
Bursts: 925
Top -12 Bottom +11 (8-oxoG) T3
Bursts: 1236
Top -12 Bottom +11 (abasic) T1
Bursts: 1080
Top -12 Bottom +11 (abasic) T2
Bursts: 1129
Top -12 Bottom +11 (abasic) T3
Bursts: 884
E
S
Bursts

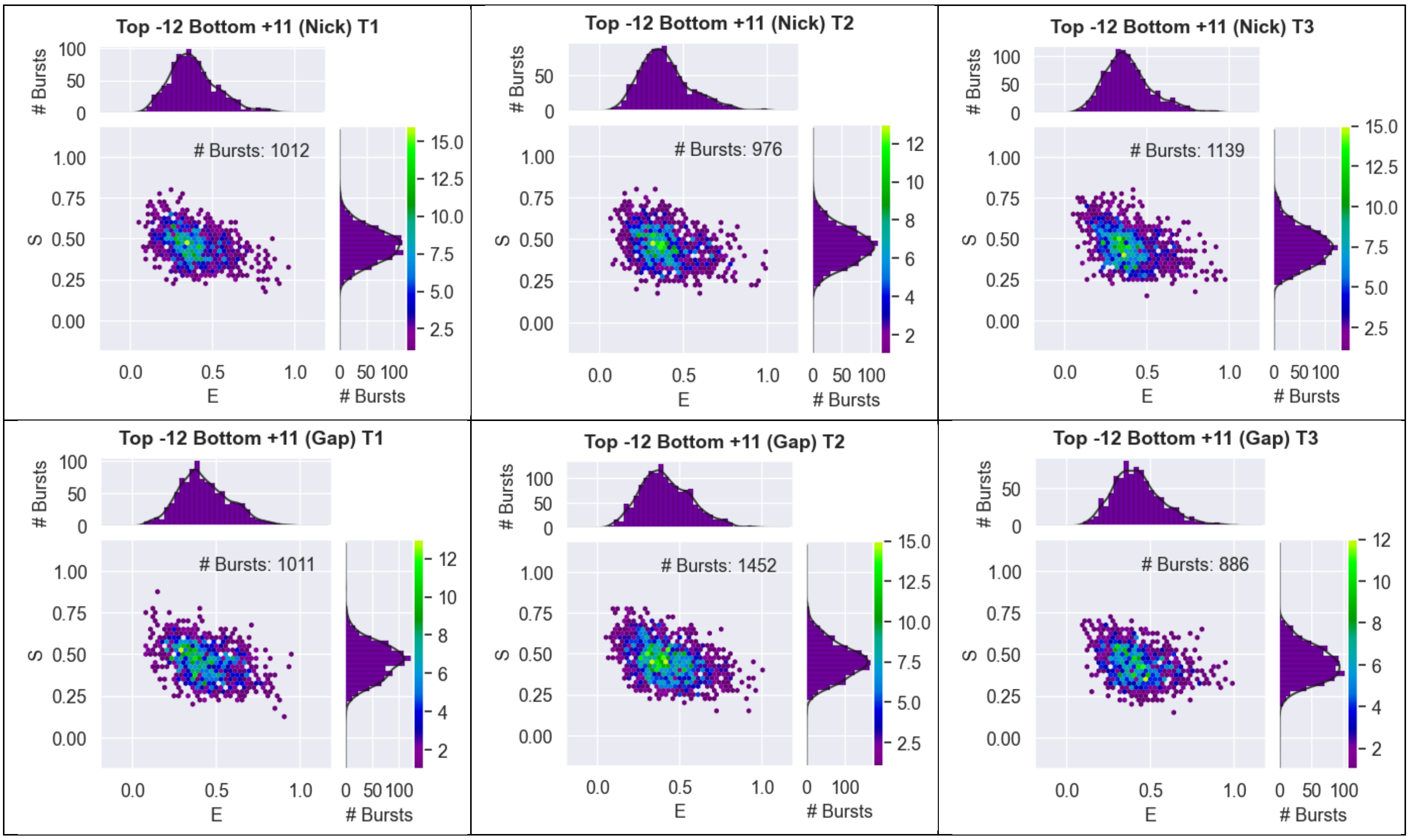

Supplementary Figure 6: 2D ES histograms from DCBS analysis showing FRET efficiency (E) and stoichiometry (S) for each independent repeat of the seven duplex constructs for the dye-pair Top -12 Bottom +11.

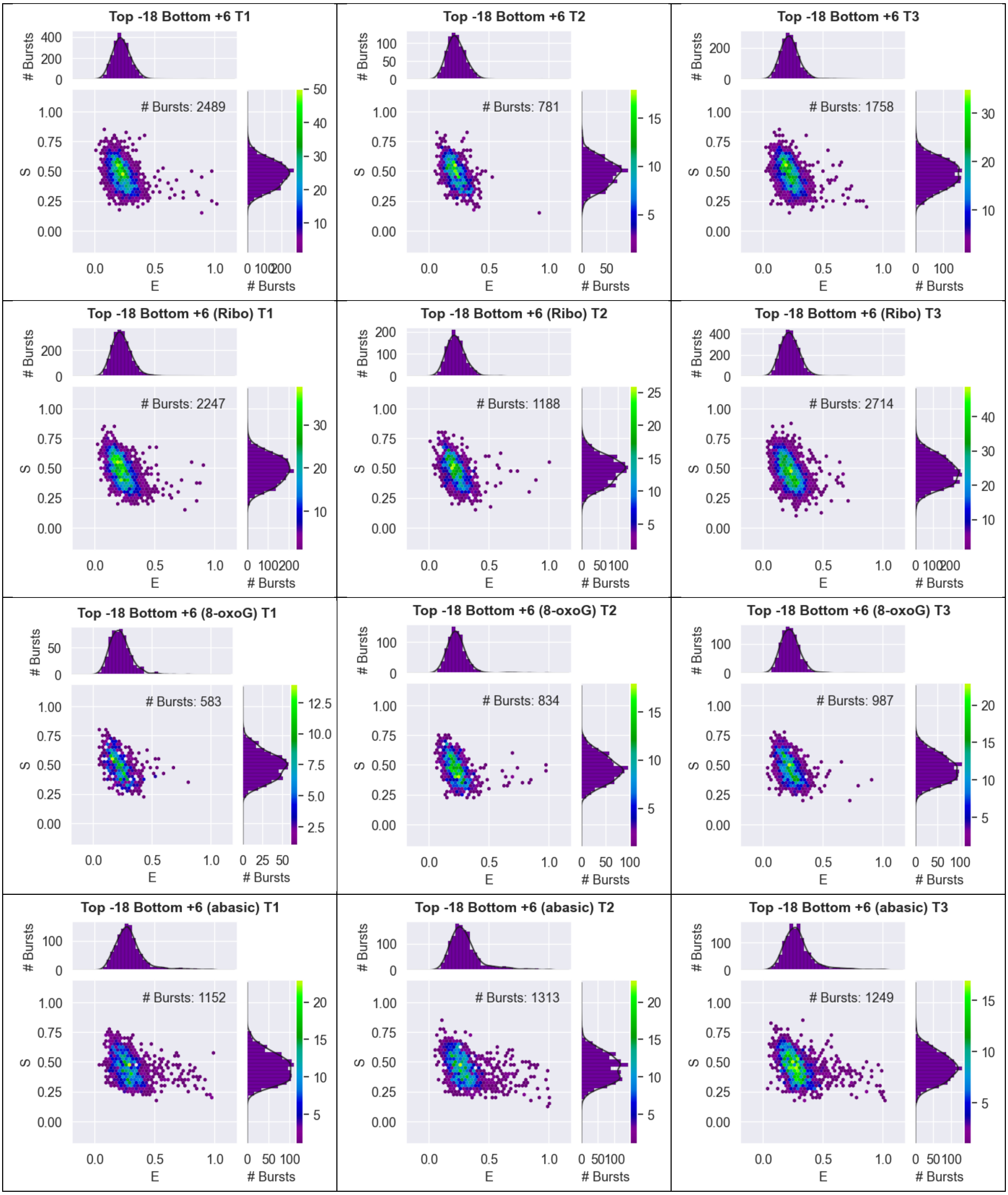
Top -18 Bottom +6 T1
Bursts: 2489
Top -18 Bottom +6 T2
Bursts: 781
Top -18 Bottom +6 T3
Bursts: 1758
Top -18 Bottom +6 (Ribo) T1
Bursts: 2247
Top -18 Bottom +6 (Ribo) T2
Bursts: 1188
Top -18 Bottom +6 (Ribo) T3
Bursts: 2714
Top -18 Bottom +6 (8-oxoG) T1
Bursts: 583
Top -18 Bottom +6 (8-oxoG) T2
Bursts: 834
Top -18 Bottom +6 (8-oxoG) T3
Bursts: 987
Top -18 Bottom +6 (abasic) T1
Bursts: 1152
Top -18 Bottom +6 (abasic) T2
Bursts: 1313
Top -18 Bottom +6 (abasic) T3
Bursts: 1249
S
E
Bursts

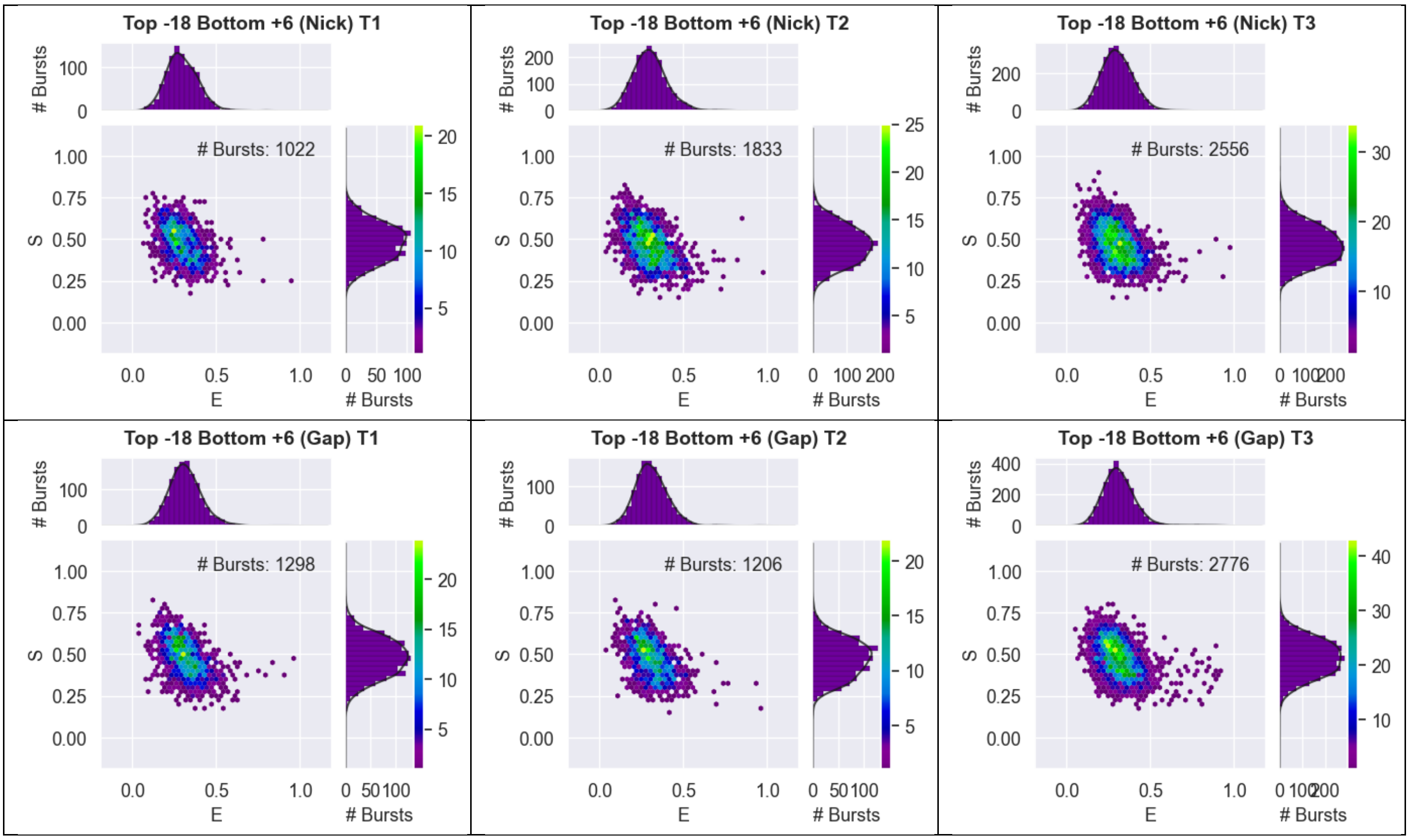

Supplementary Figure 7: 2D ES histograms from DCBS analysis showing FRET efficiency (E) and stoichiometry (S) for each independent repeat of the seven duplex constructs for the dye-pair Top -18 Bottom +6.

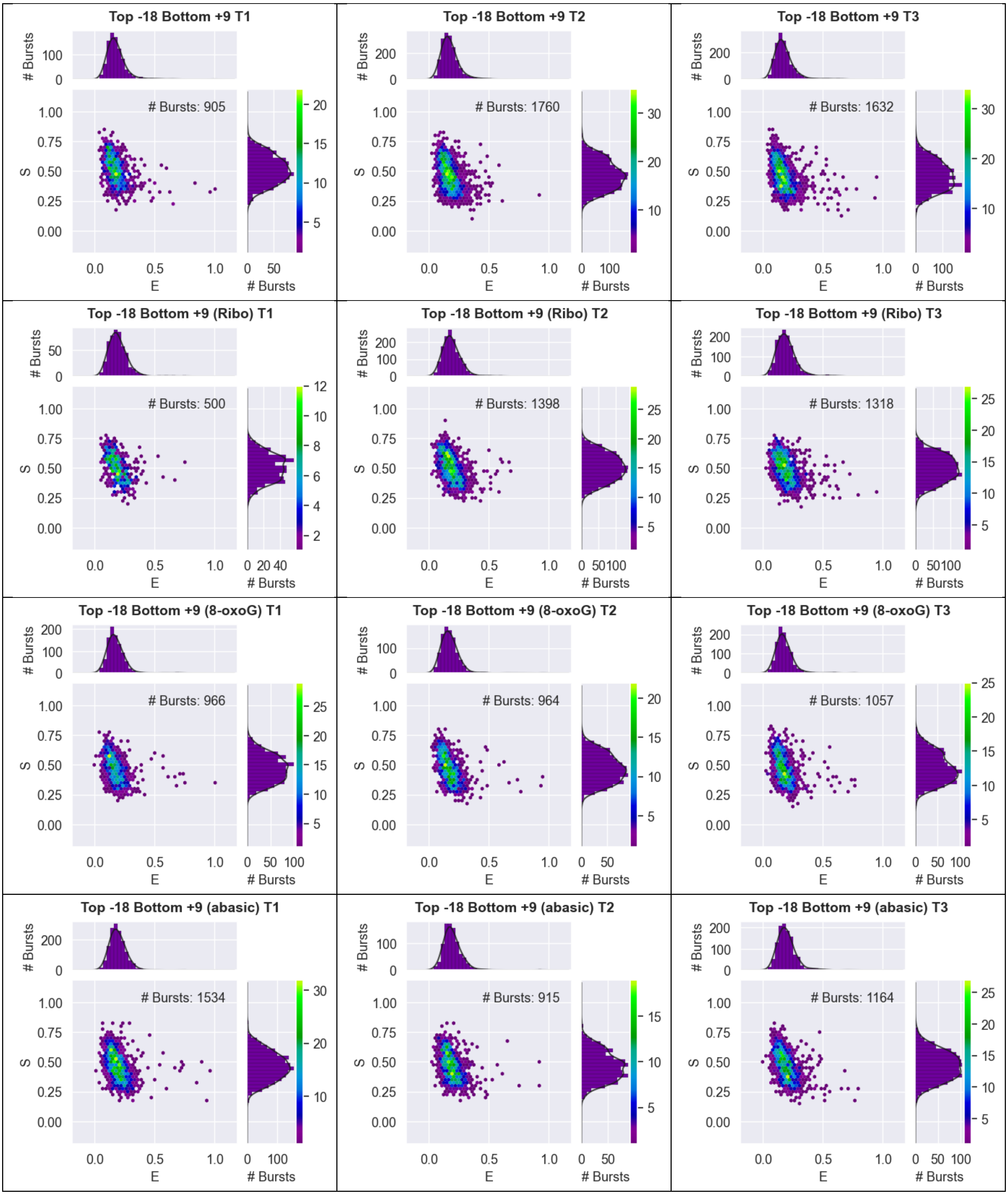
Top -18 Bottom +9 T1
Bursts: 905
Top -18 Bottom +9 T2
Bursts: 1760
Top -18 Bottom +9 T3
Bursts: 1632
Top -18 Bottom +9 (Ribo) T1
Bursts: 500
Top -18 Bottom +9 (Ribo) T2
Bursts: 1398
Top -18 Bottom +9 (Ribo) T3
Bursts: 1318
Top -18 Bottom +9 (8-oxoG) T1
Bursts: 966
Top -18 Bottom +9 (8-oxoG) T2
Bursts: 964
Top -18 Bottom +9 (8-oxoG) T3
Bursts: 1057
Top -18 Bottom +9 (abasic) T1
Bursts: 1534
Top -18 Bottom +9 (abasic) T2
Bursts: 915
Top -18 Bottom +9 (abasic) T3
Bursts: 1164

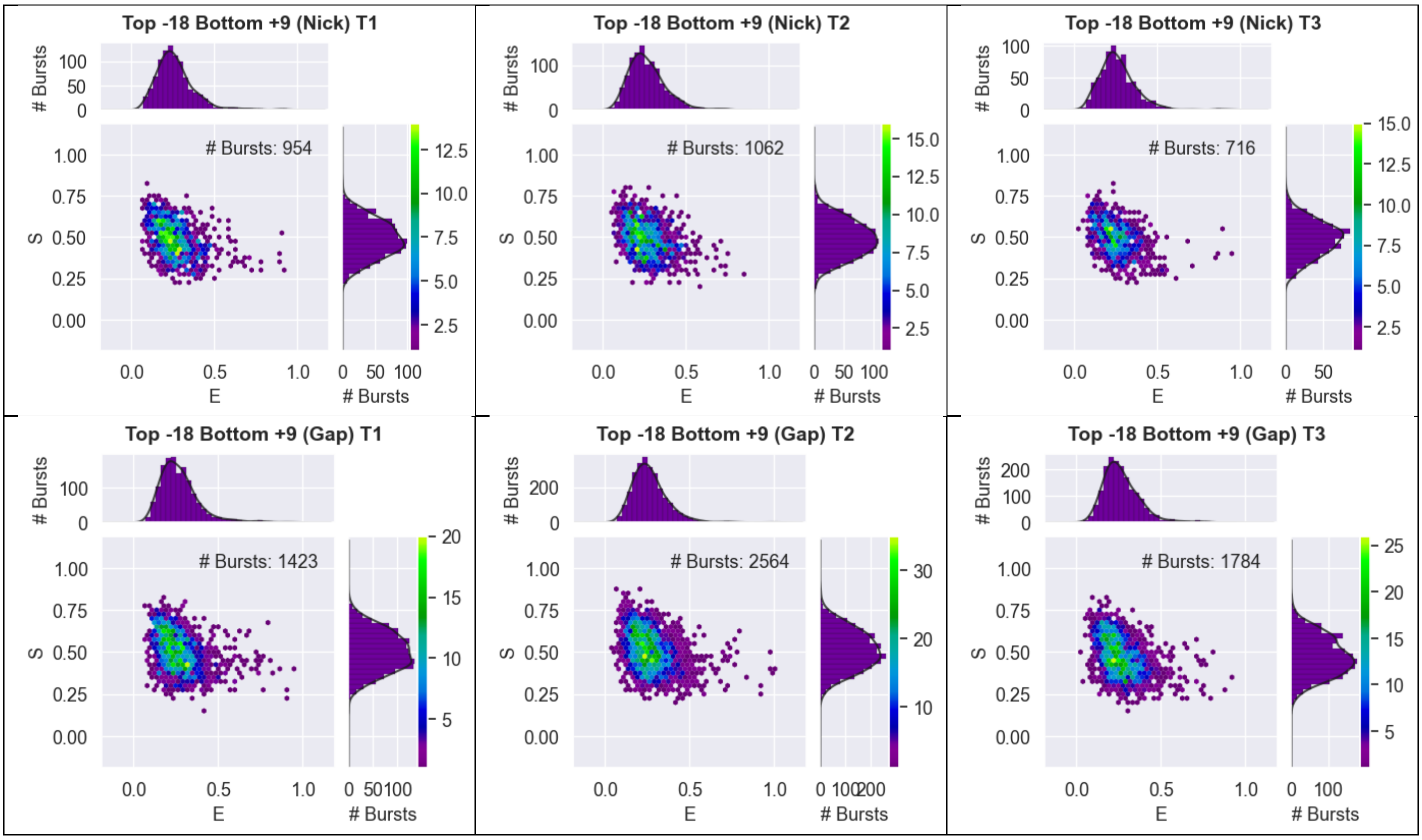

Supplementary Figure 8: 2D ES histograms from DCBS analysis showing FRET efficiency (E) and stoichiometry (S) for each independent repeat of the seven duplex constructs for the dye-pair Top -18 Bottom +9.

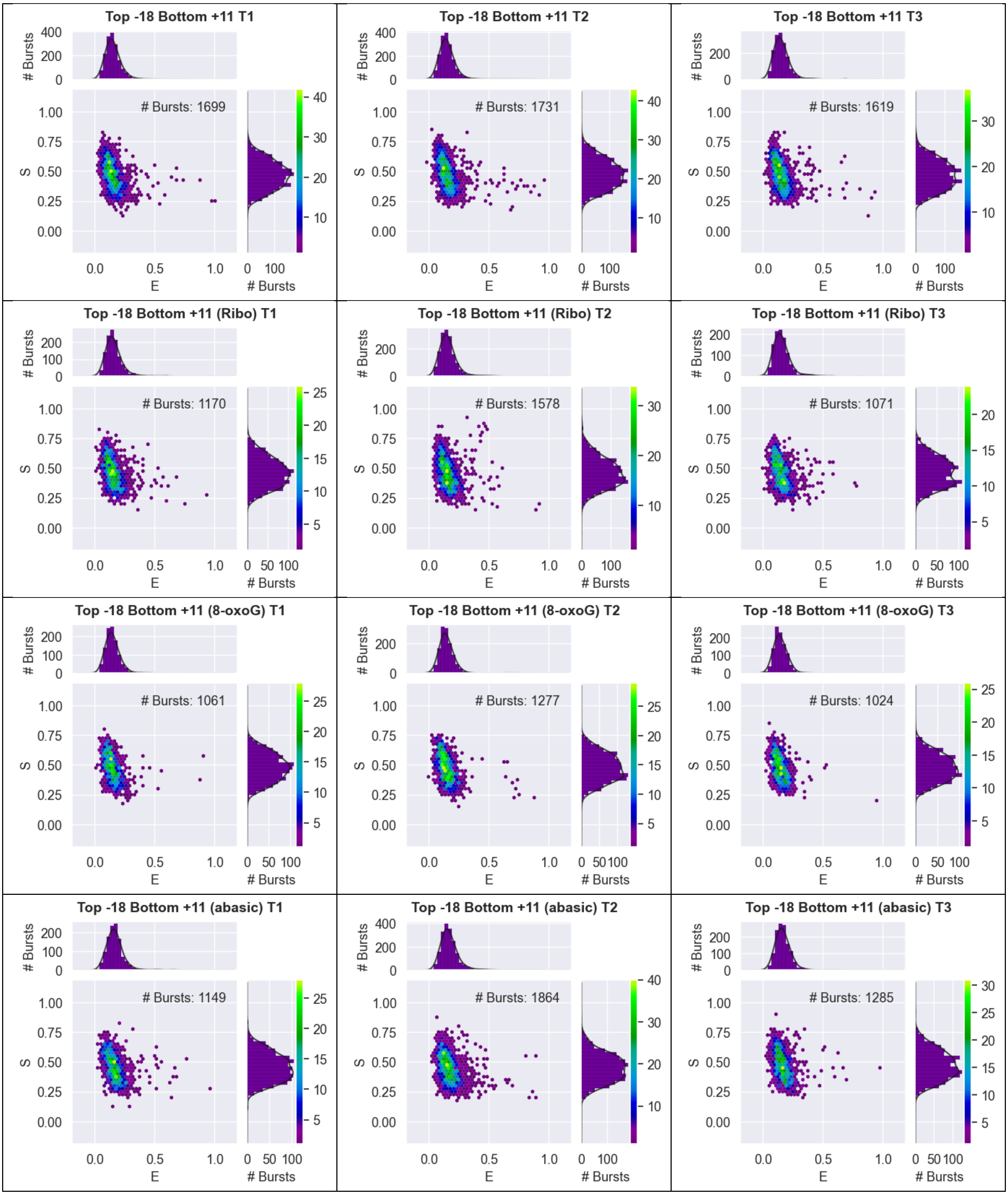
Top -18 Bottom +11 T1
Bursts: 1699
Top -18 Bottom +11 T2
Bursts: 1731
Top -18 Bottom +11 T3
Bursts: 1619
Top -18 Bottom +11 (Ribo) T1
Bursts: 1170
Top -18 Bottom +11 (Ribo) T2
Bursts: 1578
Top -18 Bottom +11 (Ribo) T3
Bursts: 1071
Top -18 Bottom +11 (8-oxoG) T1
Bursts: 1061
Top -18 Bottom +11 (8-oxoG) T2
Bursts: 1277
Top -18 Bottom +11 (8-oxoG) T3
Bursts: 1024
Top -18 Bottom +11 (abasic) T1
Bursts: 1149
Top -18 Bottom +11 (abasic) T2
Bursts: 1864
Top -18 Bottom +11 (abasic) T3
Bursts: 1285
E
S
Bursts

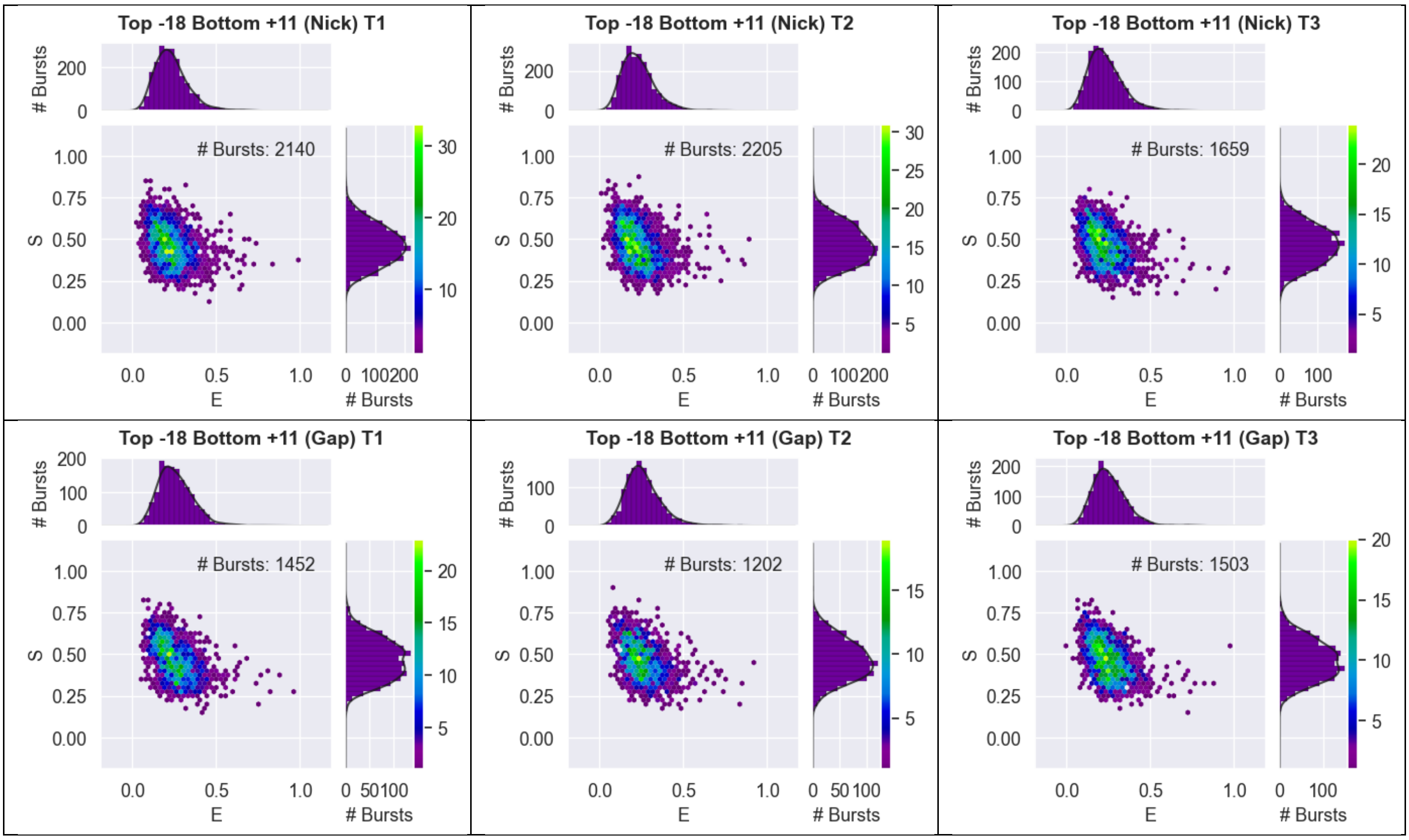

Supplementary Figure 9: 2D ES histograms from DCBS analysis showing FRET efficiency (E) and stoichiometry (S) for each independent repeat of the seven duplex constructs for the dye-pair Top -18 Bottom +11.

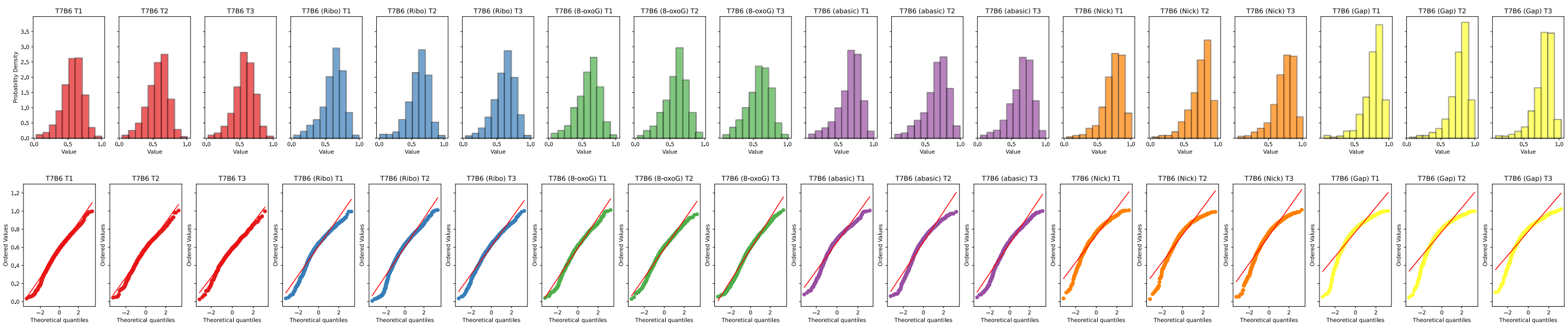

Construct	Kolmogorov–Smirnov test statistic	p-value*
T7B6 T1	0.5678	0
T7B6 T2	0.5681	0
T7B6 T3	0.565	8.634e-224
T7B6 (Ribo) T1	0.5684	0
T7B6 (Ribo) T2	0.5765	0
T7B6 (Ribo) T3	0.5808	0
T7B6 (8-oxoG) T1	0.5585	0
T7B6 (8-oxoG) T2	0.5635	0
T7B6 (8-oxoG) T3	0.5559	0
T7B6 (abasic) T1	0.5785	9.861e-255
T7B6 (abasic) T2	0.5757	0
T7B6 (abasic) T3	0.5818	0
T7B6 (Nick) T1	0.6177	0
T7B6 (Nick) T2	0.6207	0
T7B6 (Nick) T3	0.612	0
T7B6 (Gap) T1	0.6442	0
T7B6 (Gap) T2	0.6417	0
T7B6 (Gap) T3	0.646	0

*p-values given as 0 mean < 10^{-307}

Kruskal-Wallis test statistic: 5056
p-value: 0

Supplementary Figure 10: Quantile-Quantile (QQ) plots, and the Kolmogorov-Smirnov test for each independent repeat of the seven duplex constructs for the dye-pair Top -7 Bottom +6. Kruskal-Wallis test result to confirm that within all the groups there were samples originating from different distributions.

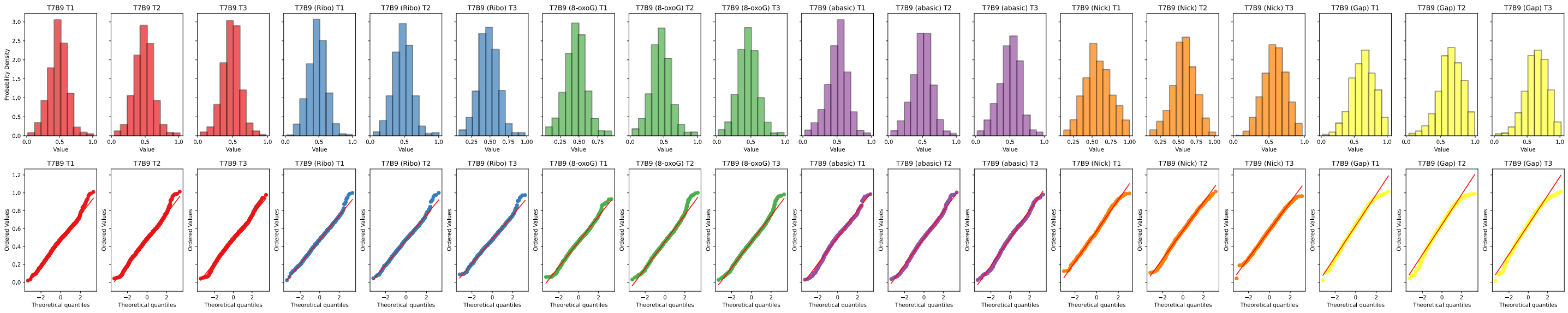

Construct	Kolmogorov–Smirnov test statistic	p-value
T7B9 T1	0.5499	0
T7B9 T2	0.5543	0
T7B9 T3	0.5593	0
T7B9 (Ribo) T1	0.559	0
T7B9 (Ribo) T2	0.5568	1.485e-290
T7B9 (Ribo) T3	0.562	8.072e-300
T7B9 (8-oxoG) T1	0.5403	0
T7B9 (8-oxoG) T2	0.5381	0
T7B9 (8-oxoG) T3	0.5429	0
T7B9 (abasic) T1	0.545	0
T7B9 (abasic) T2	0.5445	0
T7B9 (abasic) T3	0.5391	0
T7B9 (Nick) T1	0.5753	1.679e-170
T7B9 (Nick) T2	0.5719	5.625e-314
T7B9 (Nick) T3	0.583	5.777e-254
T7B9 (Gap) T1	0.5846	1.229e-302
T7B9 (Gap) T2	0.5901	0
T7B9 (Gap) T3	0.5938	0

*p-values given as 0 mean < 10^{-307}

Kruskal-Wallis test statistic: 4014
p-value: 0

Supplementary Figure 11: Quantile-Quantile (QQ) plots, and the Kolmogorov-Smirnov test for each independent repeat of the seven duplex constructs for the dye-pair Top -7 Bottom +9. Kruskal-Wallis test result to confirm that within all the groups there were samples originating from different distributions.

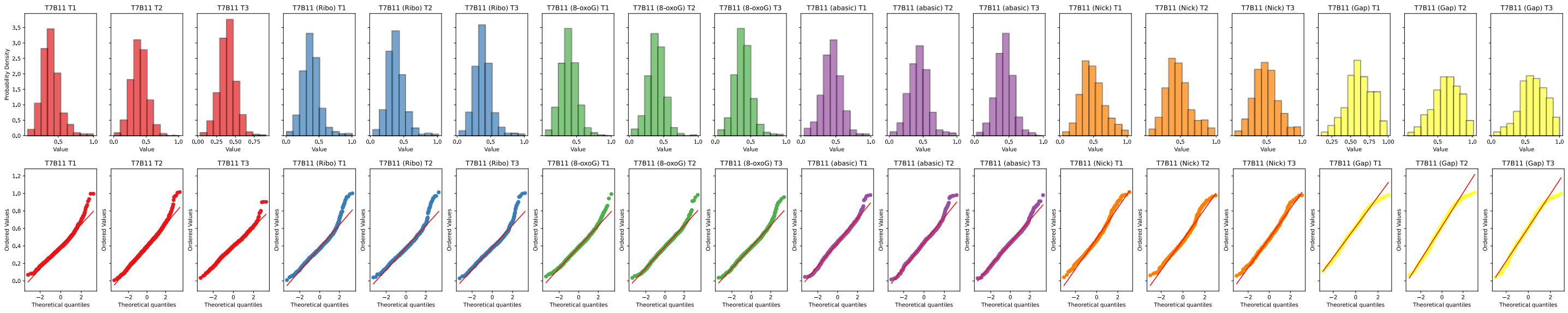

Construct	Kolmogorov–Smirnov test statistic	p-value
T7B11 T1	0.5464	8.838e-264
T7B11 T2	0.5403	0
T7B11 T3	0.5437	0
T7B11 (Ribo) T1	0.5312	0
T7B11 (Ribo) T2	0.5374	0
T7B11 (Ribo) T3	0.5373	2.941e-304
T7B11 (8-oxoG) T1	0.5407	0
T7B11 (8-oxoG) T2	0.533	0
T7B11 (8-oxoG) T3	0.5374	0
T7B11 (abasic) T1	0.5375	0
T7B11 (abasic) T2	0.5432	4.532e-293
T7B11 (abasic) T3	0.5464	0
T7B11 (Nick) T1	0.5549	3.289e-210
T7B11 (Nick) T2	0.5434	6.817e-139
T7B11 (Nick) T3	0.5515	9.672e-198
T7B11 (Gap) T1	0.5816	5.039e-128
T7B11 (Gap) T2	0.5691	5.166e-217
T7B11 (Gap) T3	0.5648	1.119e-132

*p-values given as 0 mean < 10^{-307}

Kruskal-Wallis test statistic: 2730
p-value: 0

Supplementary Figure 12: Quantile-Quantile (QQ) plots, and the Kolmogorov-Smirnov test for each independent repeat of the seven duplex constructs for the dye-pair Top -7 Bottom +11. Kruskal-Wallis test result to confirm that within all the groups there were samples originating from different distributions.

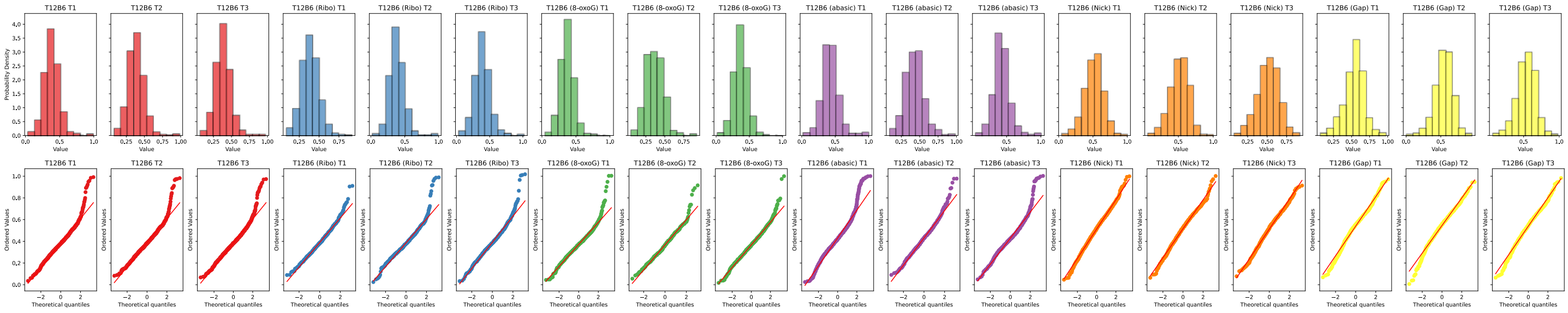

Construct	Kolmogorov–Smirnov test statistic	p-value
T12B6 T1	0.5451	0
T12B6 T2	0.5499	0
T12B6 T3	0.5475	0
T12B6 (Ribo) T1	0.5507	6.9e-261
T12B6 (Ribo) T2	0.5539	4.976e-285
T12B6 (Ribo) T3	0.5422	1.135e-257
T12B6 (8-oxoG) T1	0.5479	0
T12B6 (8-oxoG) T2	0.5419	2.703e-176
T12B6 (8-oxoG) T3	0.5457	0
T12B6 (abasic) T1	0.5524	0
T12B6 (abasic) T2	0.5533	6.657e-268
T12B6 (abasic) T3	0.5534	0
T12B6 (Nick) T1	0.5676	0
T12B6 (Nick) T2	0.5706	0
T12B6 (Nick) T3	0.5638	0
T12B6 (Gap) T1	0.5685	0
T12B6 (Gap) T2	0.5805	0
T12B6 (Gap) T3	0.5754	0

*p-values given as 0 mean < 10^{-307}

Kruskal-Wallis test statistic: 6372
p-value: 0

Supplementary Figure 13: Quantile-Quantile (QQ) plots, and the Kolmogorov-Smirnov test for each independent repeat of the seven duplex constructs for the dye-pair Top -12 Bottom +6. Kruskal-Wallis test result to confirm that within all the groups there were samples originating from different distributions.

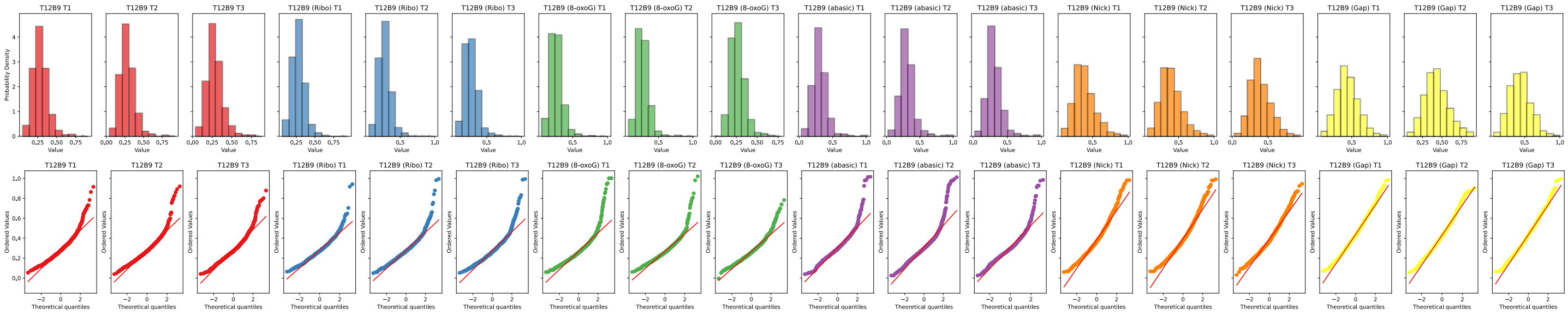

Construct	Kolmogorov–Smirnov test statistic	p-value
T12B9 T1	0.5363	0
T12B9 T2	0.5331	0
T12B9 T3	0.526	0
T12B9 (Ribo) T1	0.5357	0
T12B9 (Ribo) T2	0.5333	0
T12B9 (Ribo) T3	0.5311	0
T12B9 (8-oxoG) T1	0.5332	0
T12B9 (8-oxoG) T2	0.5354	0
T12B9 (8-oxoG) T3	0.5324	0
T12B9 (abasic) T1	0.5294	0
T12B9 (abasic) T2	0.5273	0
T12B9 (abasic) T3	0.532	3.577e-240
T12B9 (Nick) T1	0.5401	0
T12B9 (Nick) T2	0.539	0
T12B9 (Nick) T3	0.5433	0
T12B9 (Gap) T1	0.5485	0
T12B9 (Gap) T2	0.5441	1.771e-275
T12B9 (Gap) T3	0.5484	0

*p-values given as 0 mean < 10^{-307}

Kruskal-Wallis test statistic: 5735
p-value: 0

Supplementary Figure 14: Quantile-Quantile (QQ) plots, and the Kolmogorov-Smirnov test for each independent repeat of the seven duplex constructs for the dye-pair Top -12 Bottom +9. Kruskal-Wallis test result to confirm that within all the groups there were samples originating from different distributions.

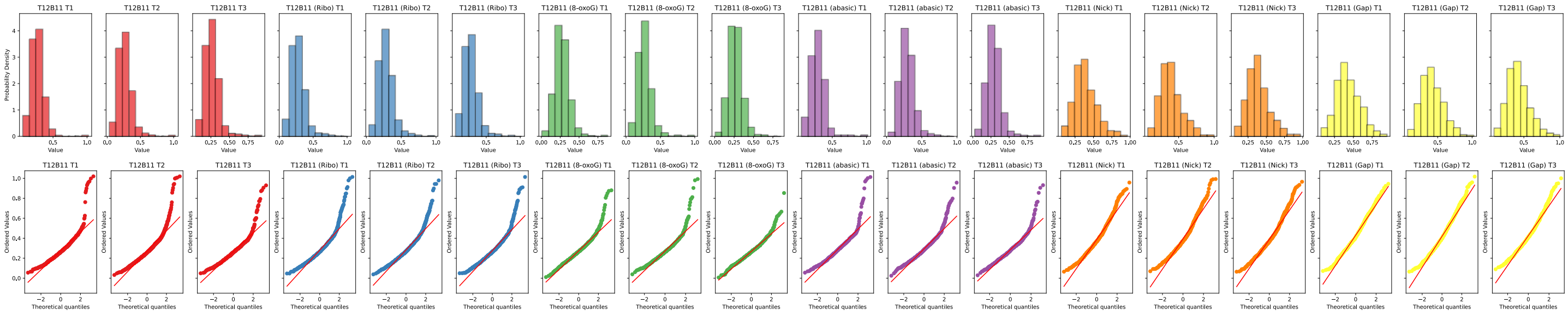

Construct	Kolmogorov–Smirnov test statistic	p-value
T12B11 T1	0.5334	0
T12B11 T2	0.5236	0
T12B11 T3	0.5299	0
T12B11 (Ribo) T1	0.5279	0
T12B11 (Ribo) T2	0.5287	0
T12B11 (Ribo) T3	0.5245	0
T12B11 (8-oxoG) T1	0.5223	0
T12B11 (8-oxoG) T2	0.5305	1.795e-243
T12B11 (8-oxoG) T3	0.5252	4.073e-318
T12B11 (abasic) T1	0.5331	3.819e-287
T12B11 (abasic) T2	0.5348	2.661e-302
T12B11 (abasic) T3	0.5283	1.33e-230
T12B11 (Nick) T1	0.5395	3.559e-276
T12B11 (Nick) T2	0.541	6.226e-268
T12B11 (Nick) T3	0.5377	1.708e-308
T12B11 (Gap) T1	0.538	3.246e-274
T12B11 (Gap) T2	0.5393	0
T12B11 (Gap) T3	0.5495	1.006e-251

*p-values given as 0 mean < 10^{-307}

Kruskal-Wallis test statistic: 4260
p-value: 0

Supplementary Figure 15: Quantile-Quantile (QQ) plots, and the Kolmogorov-Smirnov test for each independent repeat of the seven duplex constructs for the dye-pair Top -12 Bottom +11. Kruskal-Wallis test result to confirm that within all the groups there were samples originating from different distributions.

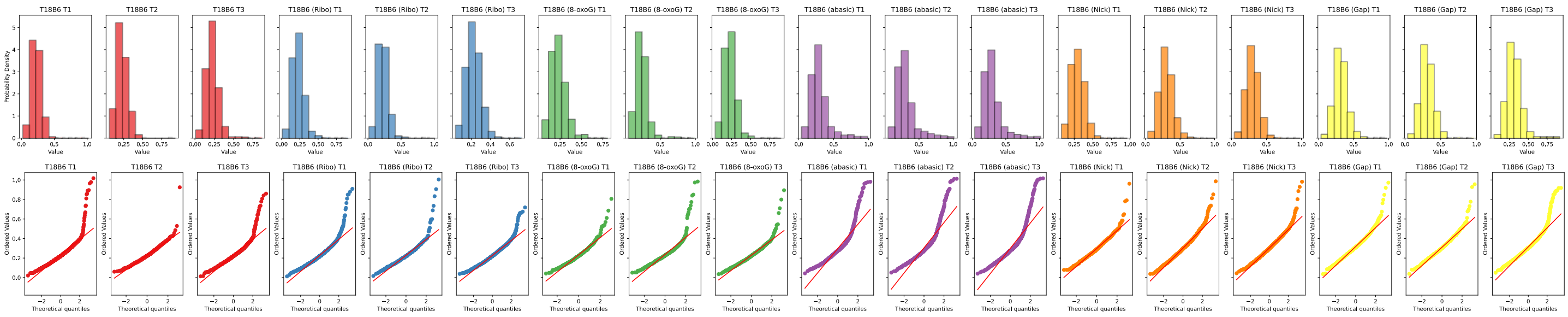

Construct	Kolmogorov–Smirnov test statistic	p-value
T18B6 T1	0.5225	0
T18B6 T2	0.5251	3.681e-201
T18B6 T3	0.5204	0
T18B6 (Ribo) T1	0.5216	0
T18B6 (Ribo) T2	0.5213	6.975e-301
T18B6 (Ribo) T3	0.5215	0
T18B6 (8-oxoG) T1	0.5241	1.219e-149
T18B6 (8-oxoG) T2	0.5243	4.467e-214
T18B6 (8-oxoG) T3	0.5235	2.094e-252
T18B6 (abasic) T1	0.5318	9.961e-305
T18B6 (abasic) T2	0.5333	0
T18B6 (abasic) T3	0.5267	0
T18B6 (Nick) T1	0.5365	1.637e-275
T18B6 (Nick) T2	0.5301	0
T18B6 (Nick) T3	0.5332	0
T18B6 (Gap) T1	0.5384	0
T18B6 (Gap) T2	0.5364	0
T18B6 (Gap) T3	0.5417	0

*p-values given as 0 mean $< 10^{-307}$

Kruskal-Wallis test statistic: 4748
p-value: 0

Supplementary Figure 16: Quantile-Quantile (QQ) plots, and the Kolmogorov-Smirnov test for each independent repeat of the seven duplex constructs for the dye-pair Top -18 Bottom +6. Kruskal-Wallis test result to confirm that within all the groups there were samples originating from different distributions.

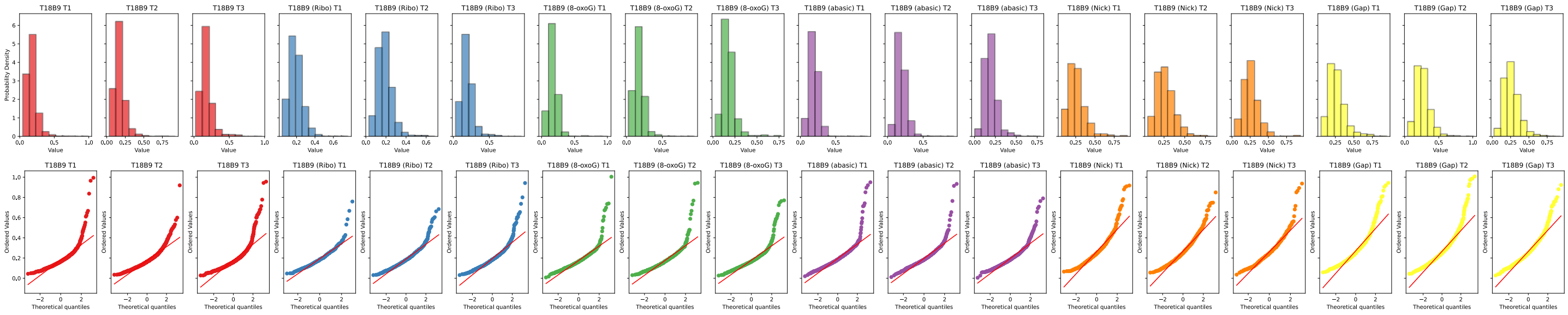

Construct	Kolmogorov–Smirnov test statistic	p-value
T18B9 T1	0.5186	7.738e-227
T18B9 T2	0.5154	0
T18B9 T3	0.5176	0
T18B9 (Ribo) T1	0.5187	1.314e-125
T18B9 (Ribo) T2	0.5151	0
T18B9 (Ribo) T3	0.5154	0
T18B9 (8-oxoG) T1	0.5142	1.191e-237
T18B9 (8-oxoG) T2	0.5135	1.621e-236
T18B9 (8-oxoG) T3	0.5151	5.028e-261
T18B9 (abasic) T1	0.5206	0
T18B9 (abasic) T2	0.515	6.448e-226
T18B9 (abasic) T3	0.5214	5.698e-295
T18B9 (Nick) T1	0.5268	2.65e-247
T18B9 (Nick) T2	0.525	2.943e-273
T18B9 (Nick) T3	0.5248	3.405e-184
T18B9 (Gap) T1	0.5314	0
T18B9 (Gap) T2	0.5272	0
T18B9 (Gap) T3	0.5277	0

*p-values given as 0 mean < 10^{-307}

Kruskal-Wallis test statistic: 4240
p-value: 0

Supplementary Figure 17: Quantile-Quantile (QQ) plots, and the Kolmogorov-Smirnov test for each independent repeat of the seven duplex constructs for the dye-pair Top -18 Bottom +9. Kruskal-Wallis test result to confirm that within all the groups there were samples originating from different distributions.

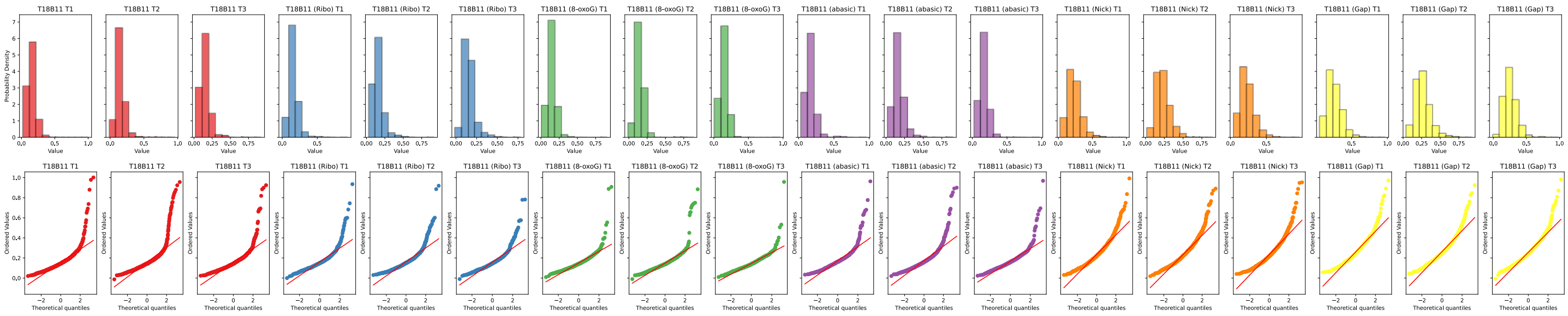

Construct	Kolmogorov–Smirnov test statistic	p-value
T18B11 T1	0.5125	0
T18B11 T2	0.5107	0
T18B11 T3	0.5123	0
T18B11 (Ribo) T1	0.5116	1.182e-284
T18B11 (Ribo) T2	0.5145	0
T18B11 (Ribo) T3	0.5095	2.648e-258
T18B11 (8-oxoG) T1	0.5136	2.506e-260
T18B11 (8-oxoG) T2	0.5104	4.507e-309
T18B11 (8-oxoG) T3	0.5098	2.688e-247
T18B11 (abasic) T1	0.5149	1.74e-283
T18B11 (abasic) T2	0.5152	0
T18B11 (abasic) T3	0.5127	5.311e-314
T18B11 (Nick) T1	0.5206	0
T18B11 (Nick) T2	0.5188	0
T18B11 (Nick) T3	0.5206	0
T18B11 (Gap) T1	0.5241	0
T18B11 (Gap) T2	0.5228	2.205e-306
T18B11 (Gap) T3	0.5236	0

*p-values given as 0 mean < 10^{-307}

Kruskal-Wallis test statistic: 6230
p-value: 0

Supplementary Figure 18: Quantile-Quantile (QQ) plots, and the Kolmogorov-Smirnov test for each independent repeat of the seven duplex constructs for the dye-pair Top -18 Bottom +11. Kruskal-Wallis test result to confirm that within all the groups there were samples originating from different distributions.

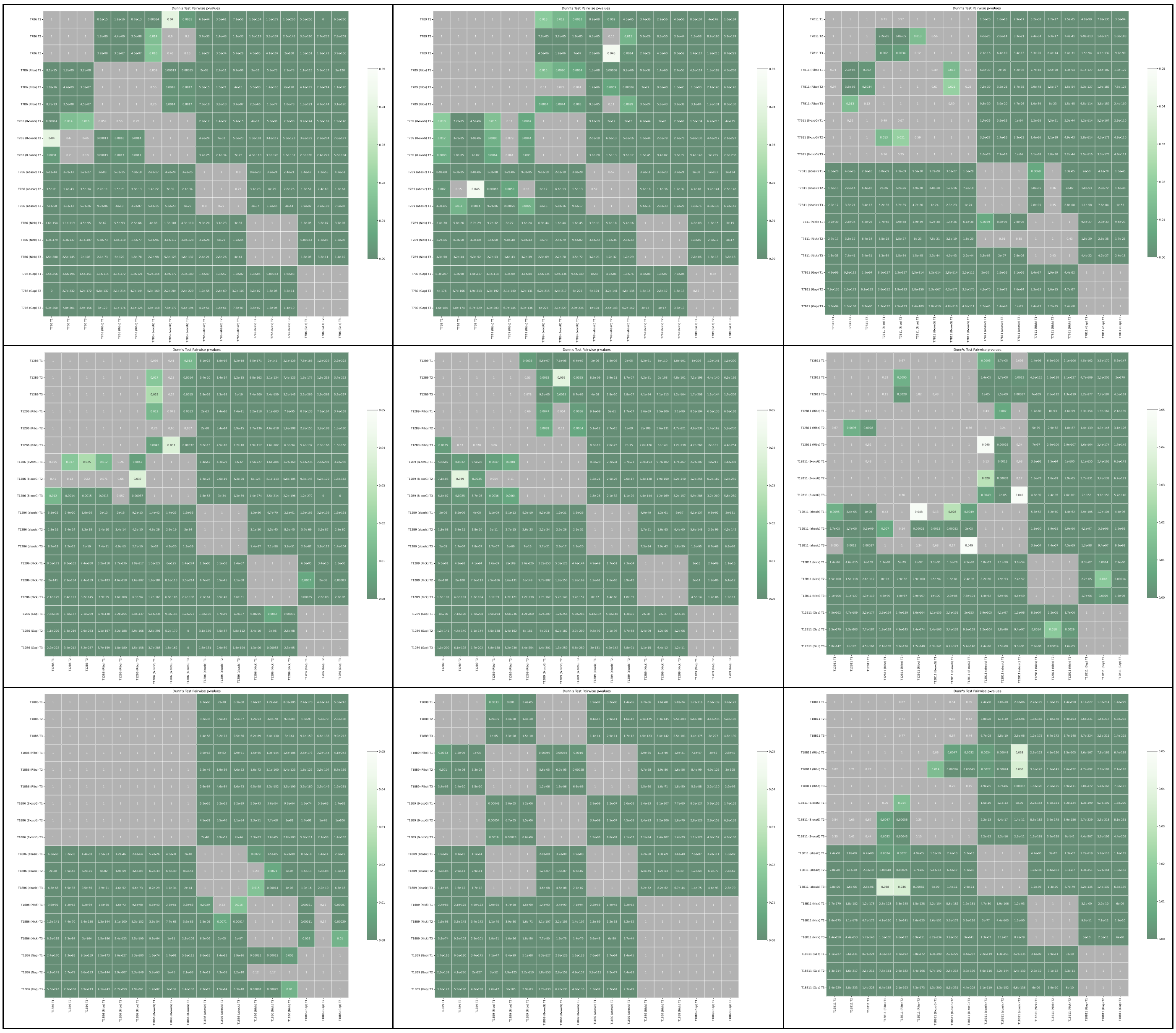

Supplementary Figure 19: Dunn's Tests for each of the repeats for each of the labelling positions for each of the different types of damage.

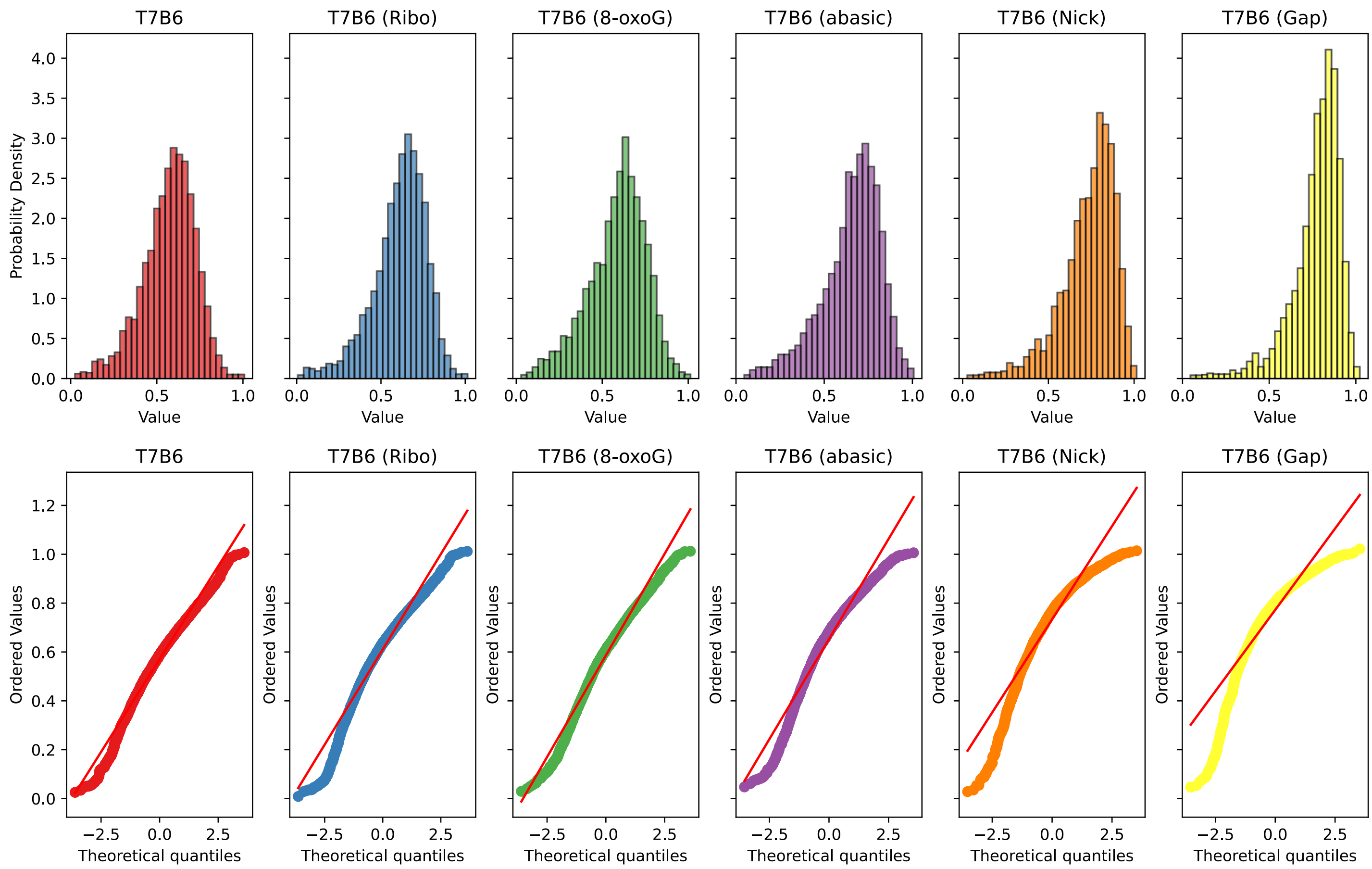

Construct	Kolmogorov–Smirnov test statistic	p-value*
T7B6	0.5663	0
T7B6 (Ribo)	0.5734	0
T7B6 (8-oxoG)	0.5565	0
T7B6 (abasic)	0.5771	0
T7B6 (Nick)	0.6143	0
T7B6 (Gap)	0.6424	0

*p-values given as 0 mean < 10^{-307}

Kruskal-Wallis test statistic: 5041
p-value: 0

Supplementary Figure 20: Quantile-Quantile (QQ) plots, and the Kolmogorov-Smirnov test for each combined repeats of the seven duplex constructs for the dye-pair Top -7 Bottom +6. Kruskal-Wallis test result to confirm that within all the groups there were samples originating from different distributions.

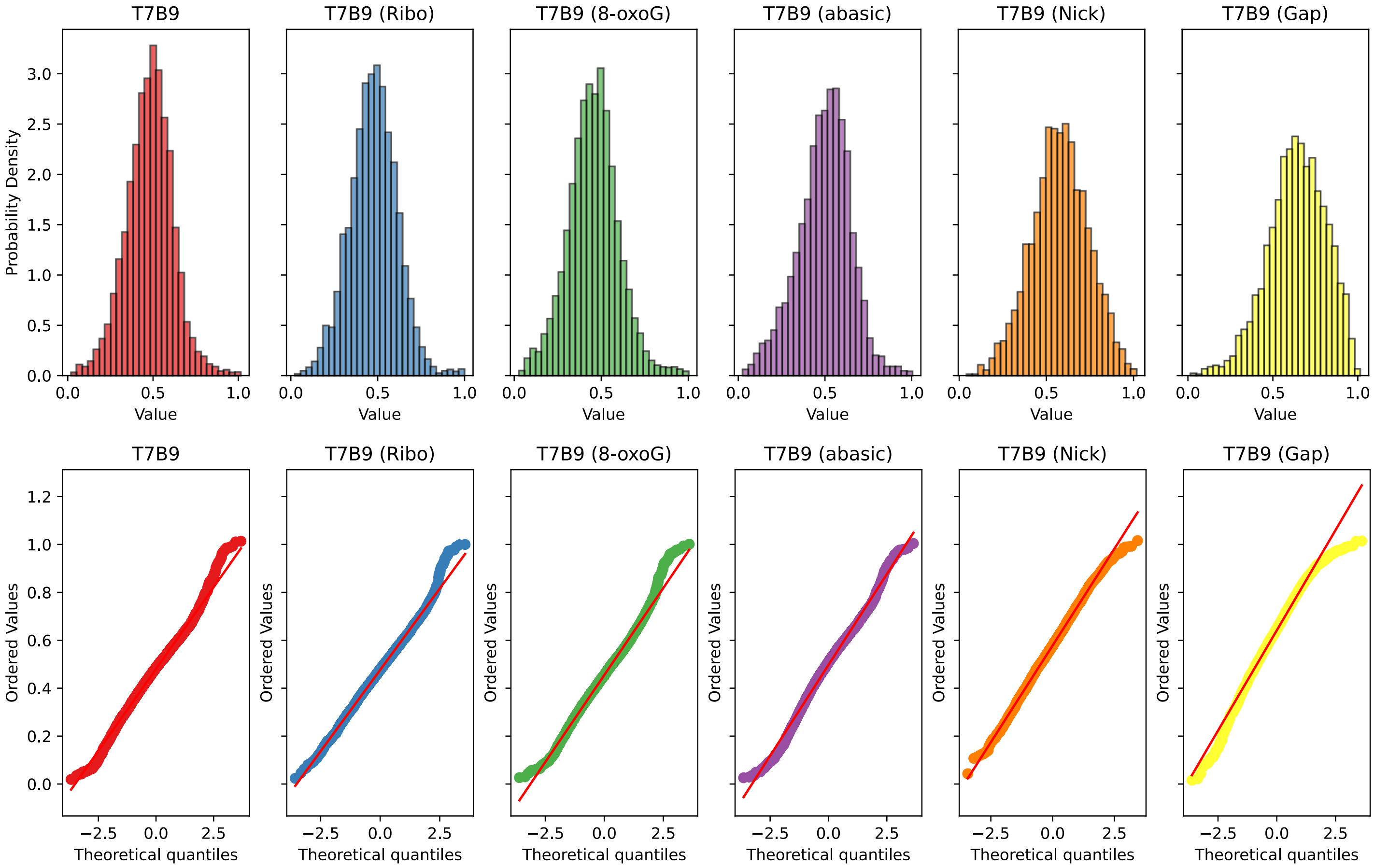

Construct	Kolmogorov–Smirnov test statistic	p-value*
T7B9	0.5551	0
T7B9 (Ribo)	0.5578	0
T7B9 (8-oxoG)	0.5394	0
T7B9 (abasic)	0.5414	0
T7B9 (Nick)	0.5746	0
T7B9 (Gap)	0.5892	0

*p-values given as 0 mean $< 10^{-307}$

Kruskal-Wallis test statistic: 3998
p-value: 0

Supplementary Figure 21: Quantile-Quantile (QQ) plots, and the Kolmogorov-Smirnov test for each combined repeats of the seven duplex constructs for the dye-pair Top -7 Bottom +9. Kruskal-Wallis test result to confirm that within all the groups there were samples originating from different distributions.

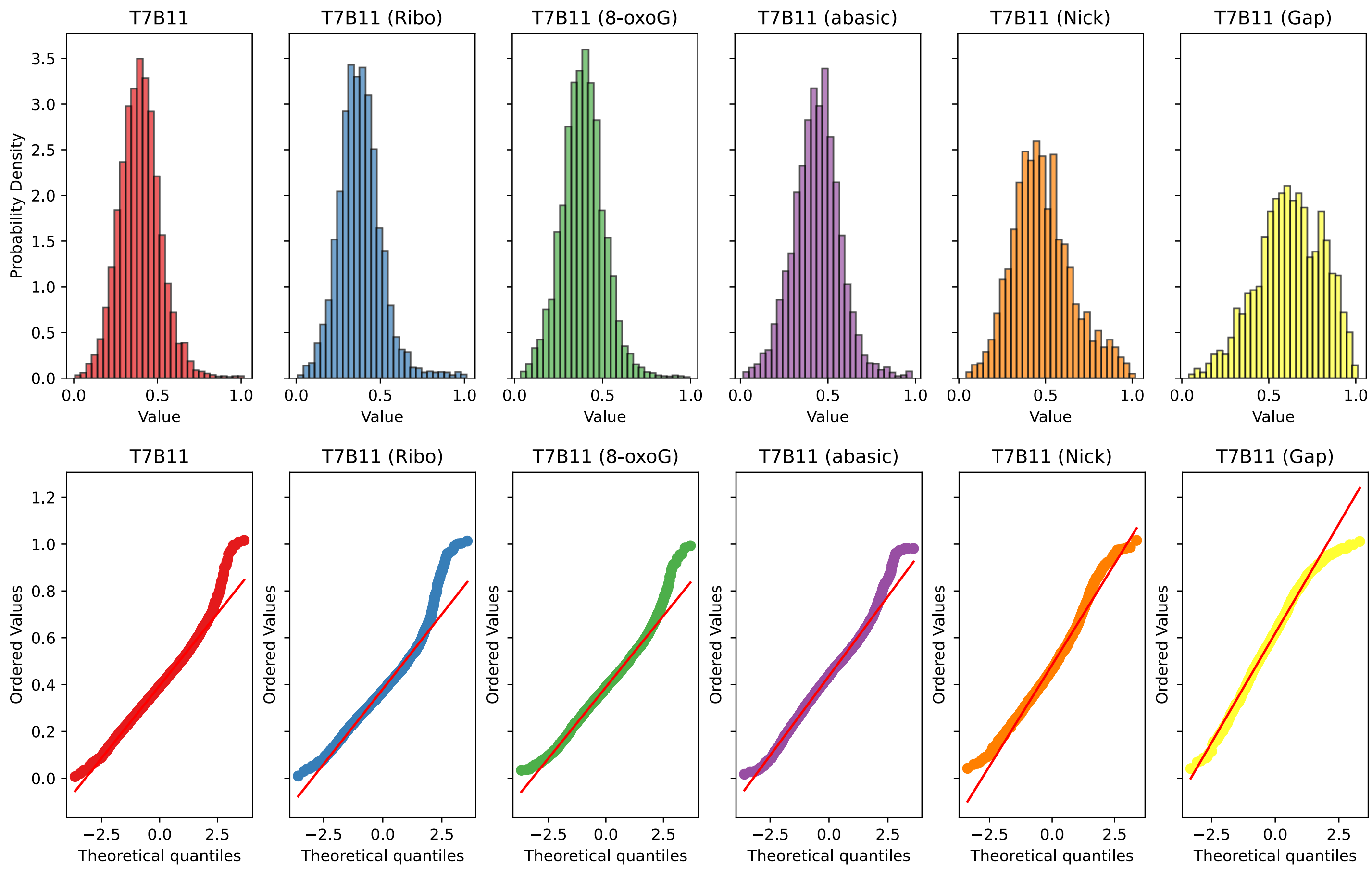

Construct	Kolmogorov–Smirnov test statistic	p-value*
T7B11	0.5408	0
T7B11 (Ribo)	0.5342	0
T7B11 (8-oxoG)	0.5362	0
T7B11 (abasic)	0.5421	0
T7B11 (Nick)	0.5483	0
T7B11 (Gap)	0.5684	0

*p-values given as 0 mean < 10^{-307}

Kruskal-Wallis test statistic: 2714
p-value: 0

Supplementary Figure 22: Quantile-Quantile (QQ) plots, and the Kolmogorov-Smirnov test for each combined repeats of the seven duplex constructs for the dye-pair Top -7 Bottom +11. Kruskal-Wallis test result to confirm that within all the groups there were samples originating from different distributions.

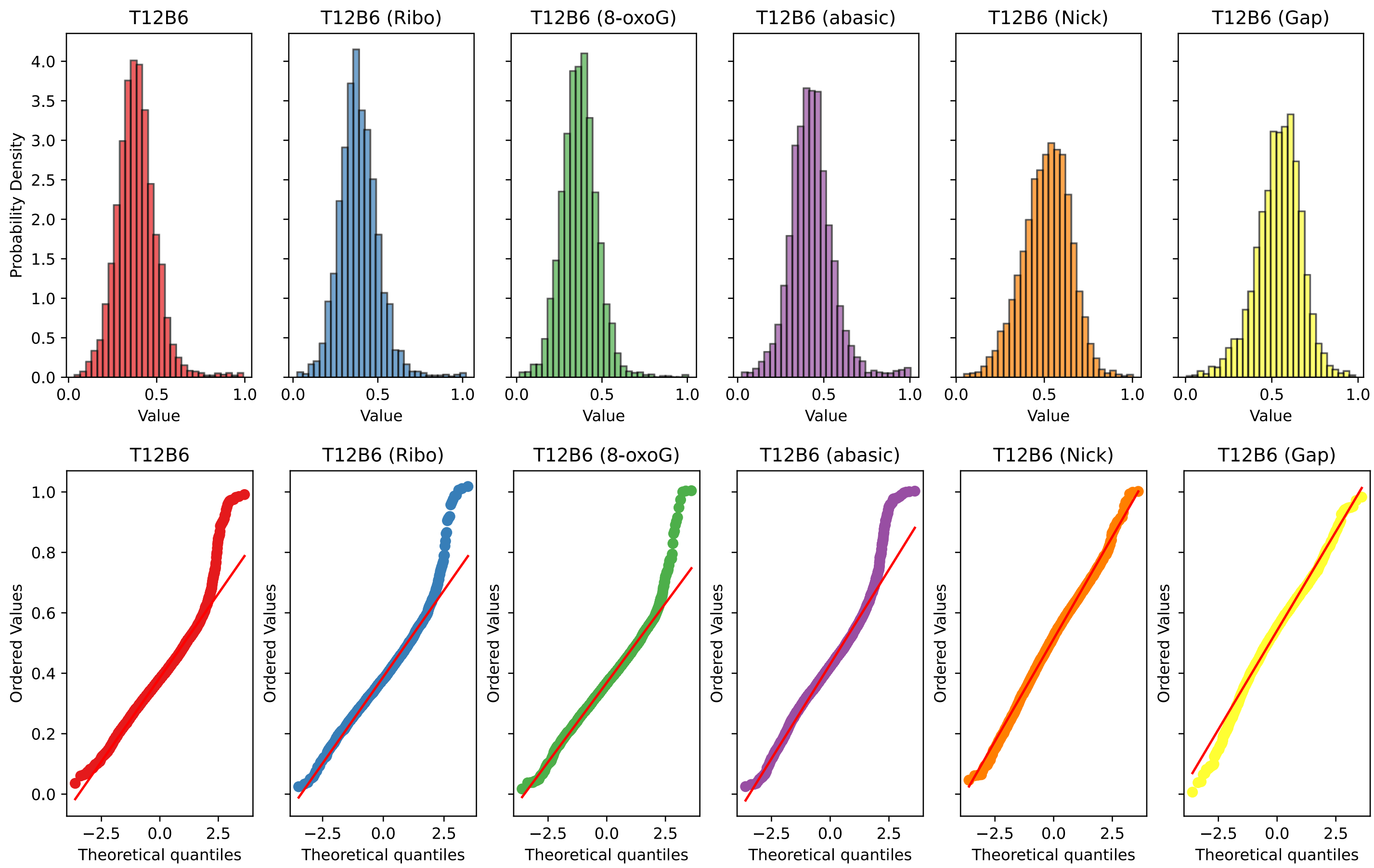

Construct	Kolmogorov–Smirnov test statistic	p-value*
T12B6	0.546	0
T12B6 (Ribo)	0.5473	0
T12B6 (8-oxoG)	0.5452	0
T12B6 (abasic)	0.5514	0
T12B6 (Nick)	0.5664	0
T12B6 (Gap)	0.5741	0

*p-values given as 0 mean < 10^{-307}

Kruskal-Wallis test statistic: 6372
p-value: 0

Supplementary Figure 23: Quantile-Quantile (QQ) plots, and the Kolmogorov-Smirnov test for each combined repeats of the seven duplex constructs for the dye-pair Top -12 Bottom +6. Kruskal-Wallis test result to confirm that within all the groups there were samples originating from different distributions.

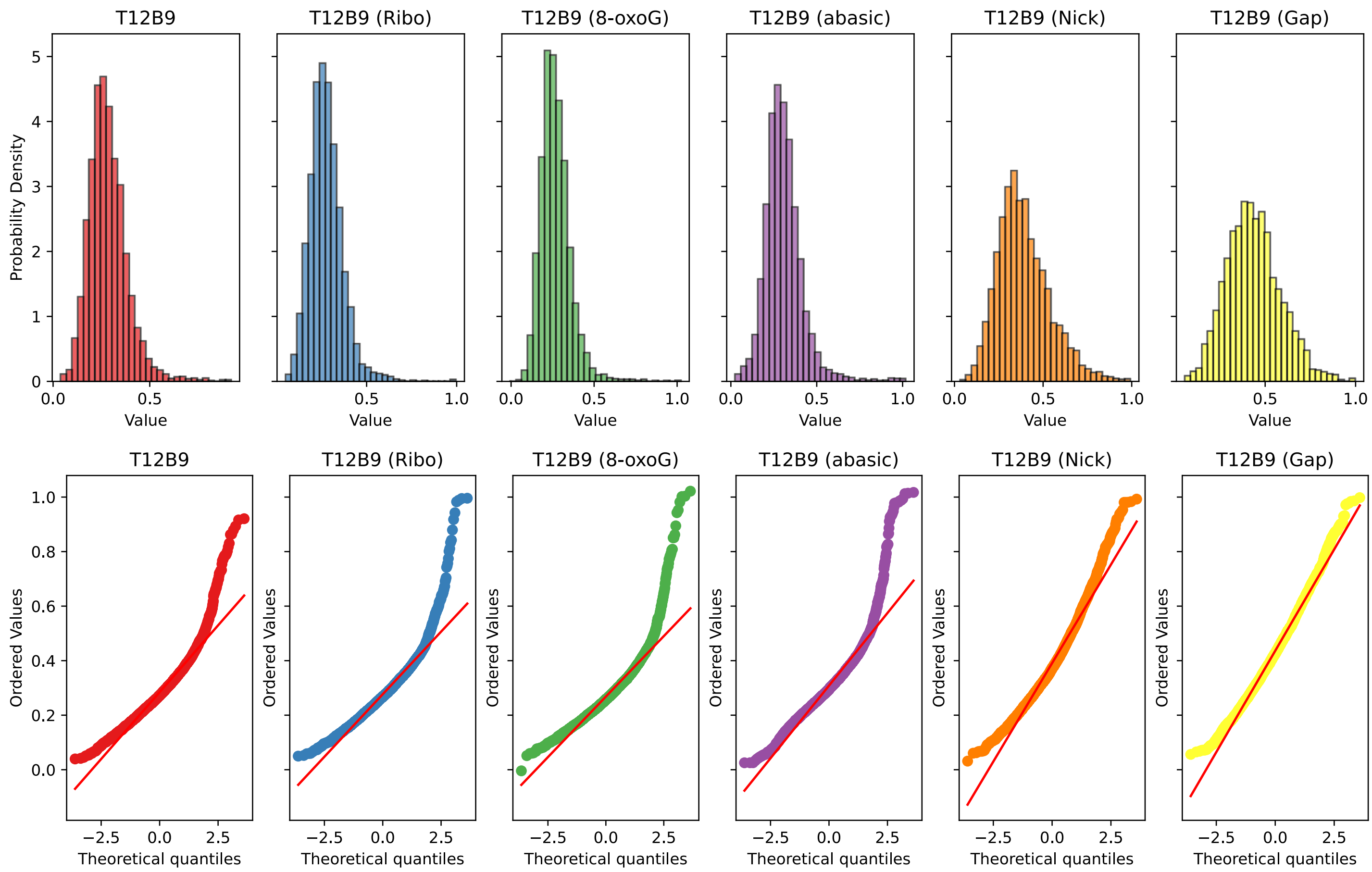

Construct	Kolmogorov–Smirnov test statistic	p-value*
T12B9	0.5308	0
T12B9 (Ribo)	0.5324	0
T12B9 (8-oxoG)	0.5328	0
T12B9 (abasic)	0.5281	0
T12B9 (Nick)	0.5399	0
T12B9 (Gap)	0.5468	0

*p-values given as 0 mean $< 10^{-307}$

Kruskal-Wallis test statistic: 5735
p-value: 0

Supplementary Figure 24: Quantile-Quantile (QQ) plots, and the Kolmogorov-Smirnov test for each combined repeats of the seven duplex constructs for the dye-pair Top -12 Bottom +9. Kruskal-Wallis test result to confirm that within all the groups there were samples originating from different distributions.

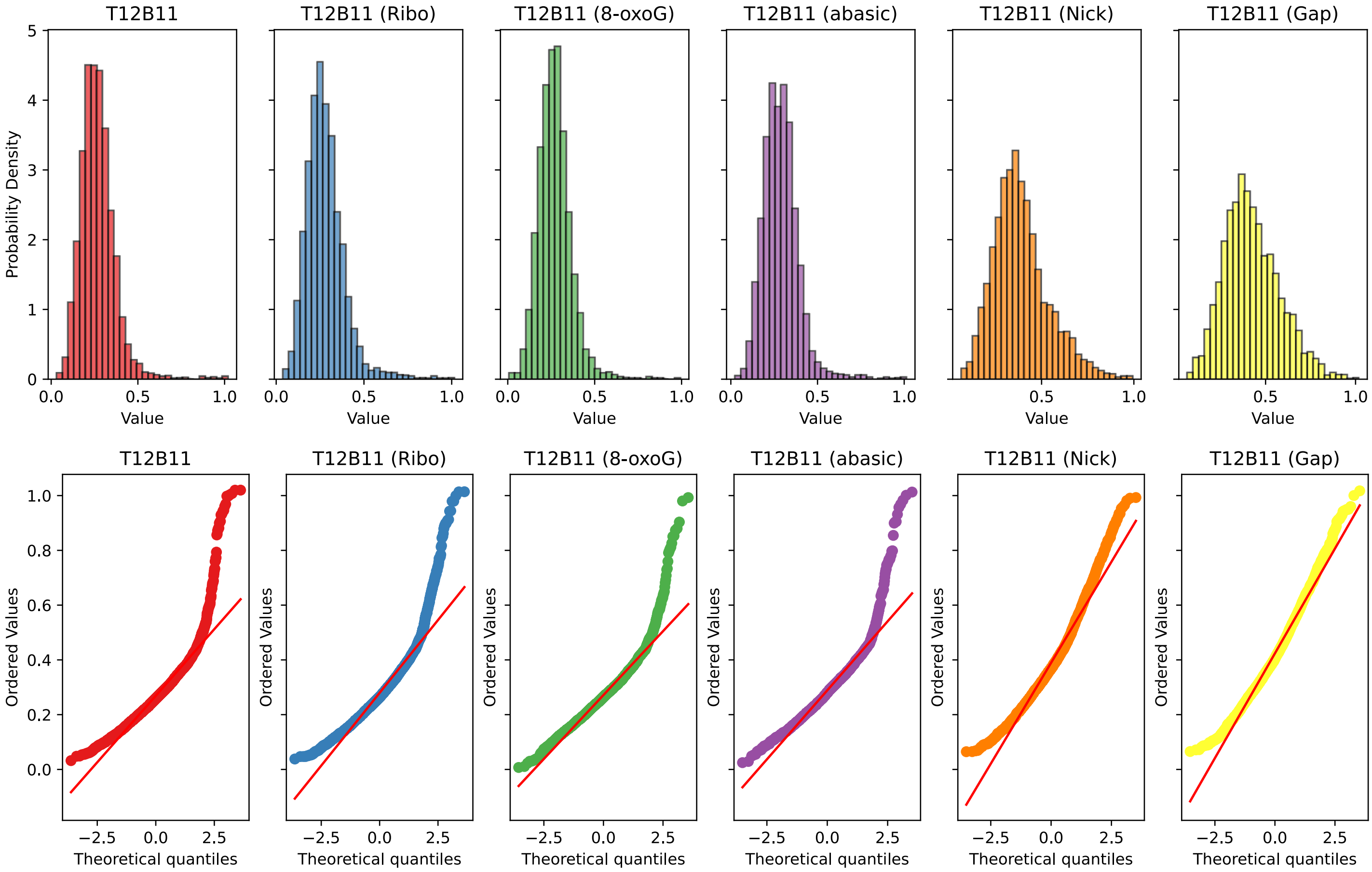

Construct	Kolmogorov–Smirnov test statistic	p-value*
T12B11	0.5277	0
T12B11 (Ribo)	0.5268	0
T12B11 (8-oxoG)	0.5243	0
T12B11 (abasic)	0.5314	0
T12B11 (Nick)	0.5388	0
T12B11 (Gap)	0.5408	0

*p-values given as 0 mean < 10^{-307}

Kruskal-Wallis test statistic: 4260
p-value: 0

Supplementary Figure 25: Quantile-Quantile (QQ) plots, and the Kolmogorov-Smirnov test for each combined repeats of the seven duplex constructs for the dye-pair Top -12 Bottom +11. Kruskal-Wallis test result to confirm that within all the groups there were samples originating from different distributions.

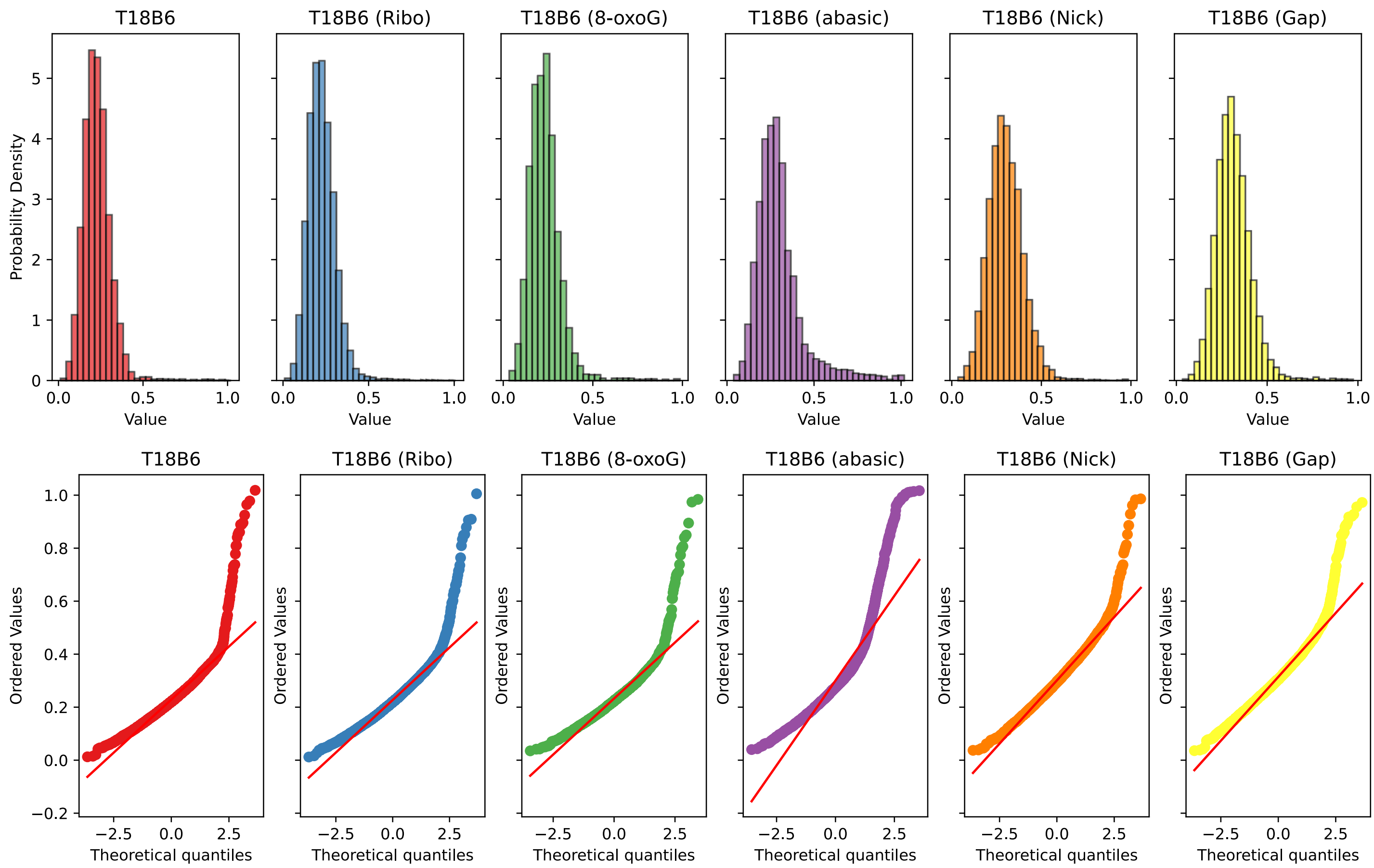

Construct	Kolmogorov–Smirnov test statistic	p-value*
T18B6	0.5216	0
T18B6 (Ribo)	0.5211	0
T18B6 (8-oxoG)	0.5229	0
T18B6 (abasic)	0.5301	0
T18B6 (Nick)	0.532	0
T18B6 (Gap)	0.5391	0

*p-values given as 0 mean $< 10^{-307}$

Kruskal-Wallis test statistic: 4738
p-value: 0

Supplementary Figure 26: Quantile-Quantile (QQ) plots, and the Kolmogorov-Smirnov test for each combined repeats of the seven duplex constructs for the dye-pair Top -18 Bottom +6. Kruskal-Wallis test result to confirm that within all the groups there were samples originating from different distributions.

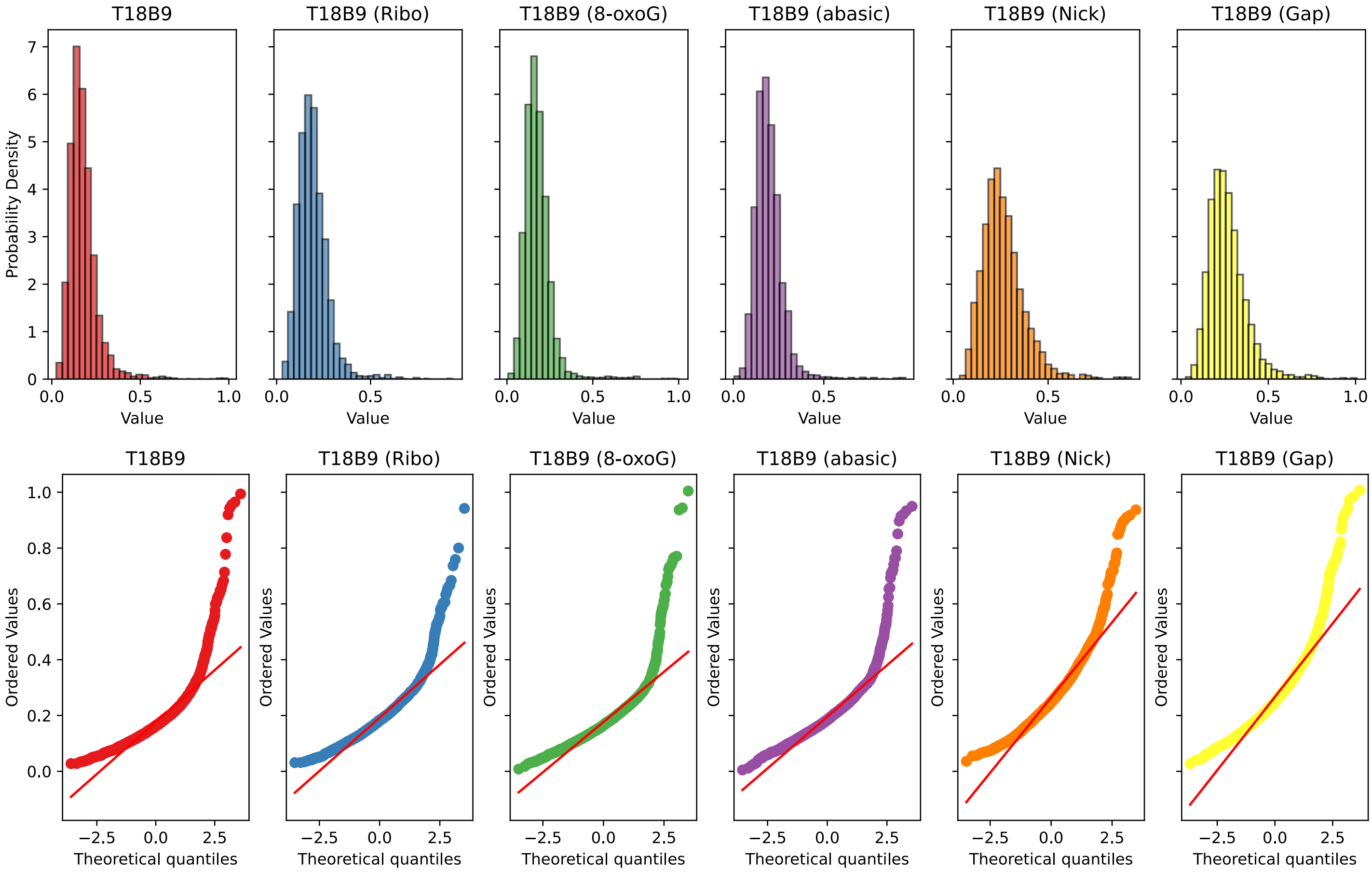

Construct	Kolmogorov–Smirnov test statistic	p-value*
T18B9	0.5165	0
T18B9 (Ribo)	0.5153	0
T18B9 (8-oxoG)	0.5136	0
T18B9 (abasic)	0.5182	0
T18B9 (Nick)	0.5246	0
T18B9 (Gap)	0.5279	0

*p-values given as 0 mean < 10^{-307}

Kruskal-Wallis test statistic: 4236
p-value: 0

Supplementary Figure 27: Quantile-Quantile (QQ) plots, and the Kolmogorov-Smirnov test for each combined repeats of the seven duplex constructs for the dye-pair Top -18 Bottom +9. Kruskal-Wallis test result to confirm that within all the groups there were samples originating from different distributions.

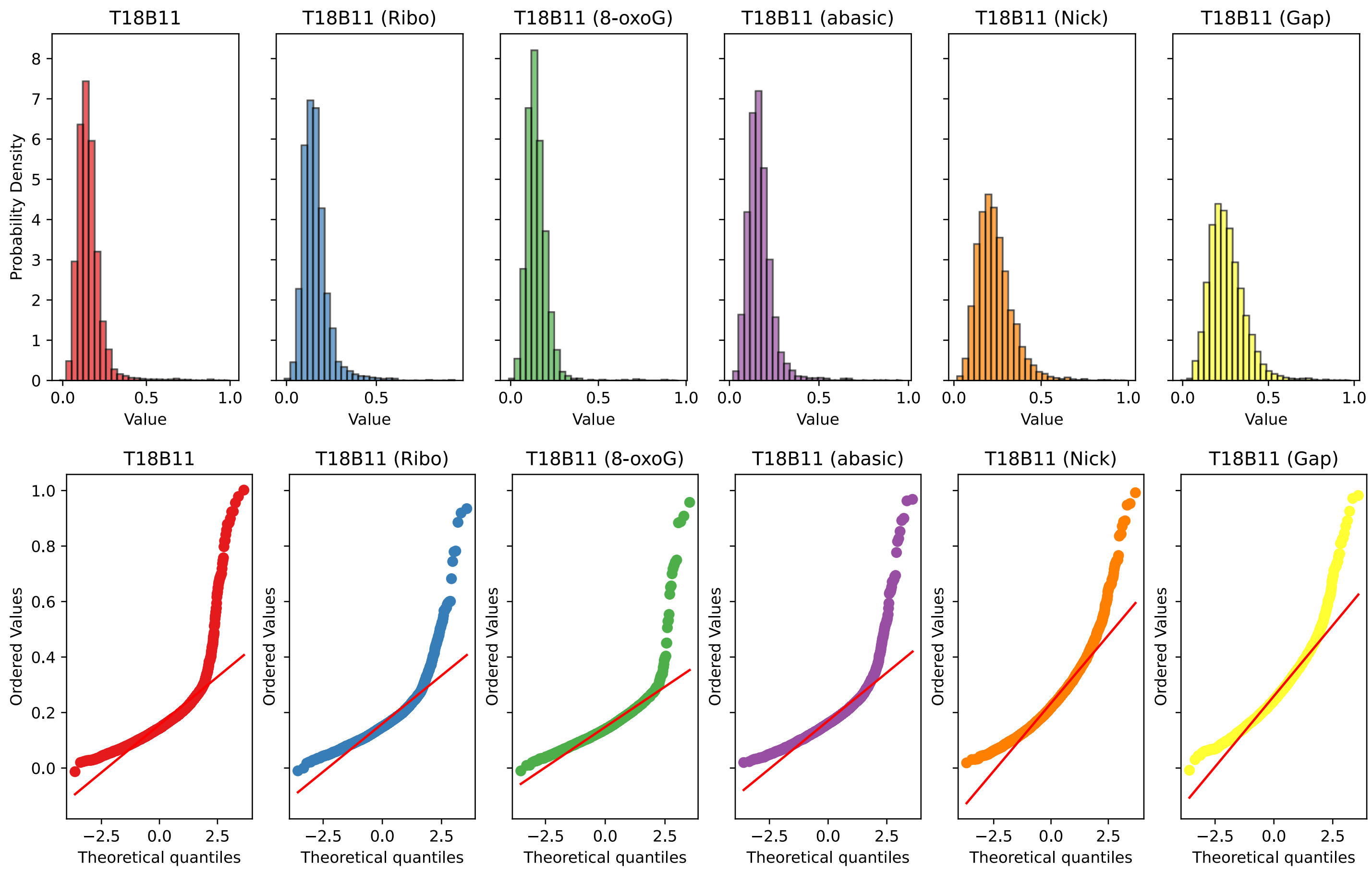

Construct	Kolmogorov–Smirnov test statistic	p-value*
T18B11	0.511	0
T18B11 (Ribo)	0.5113	0
T18B11 (8-oxoG)	0.5104	0
T18B11 (abasic)	0.5142	0
T18B11 (Nick)	0.5188	0
T18B11 (Gap)	0.5231	0

*p-values given as 0 mean < 10^{-307}

Kruskal-Wallis test statistic: 6225
p-value: 0

Supplementary Figure 28: Quantile-Quantile (QQ) plots, and the Kolmogorov-Smirnov test for each combined repeats of the seven duplex constructs for the dye-pair Top -18 Bottom +11. Kruskal-Wallis test result to confirm that within all the groups there were samples originating from different distributions.

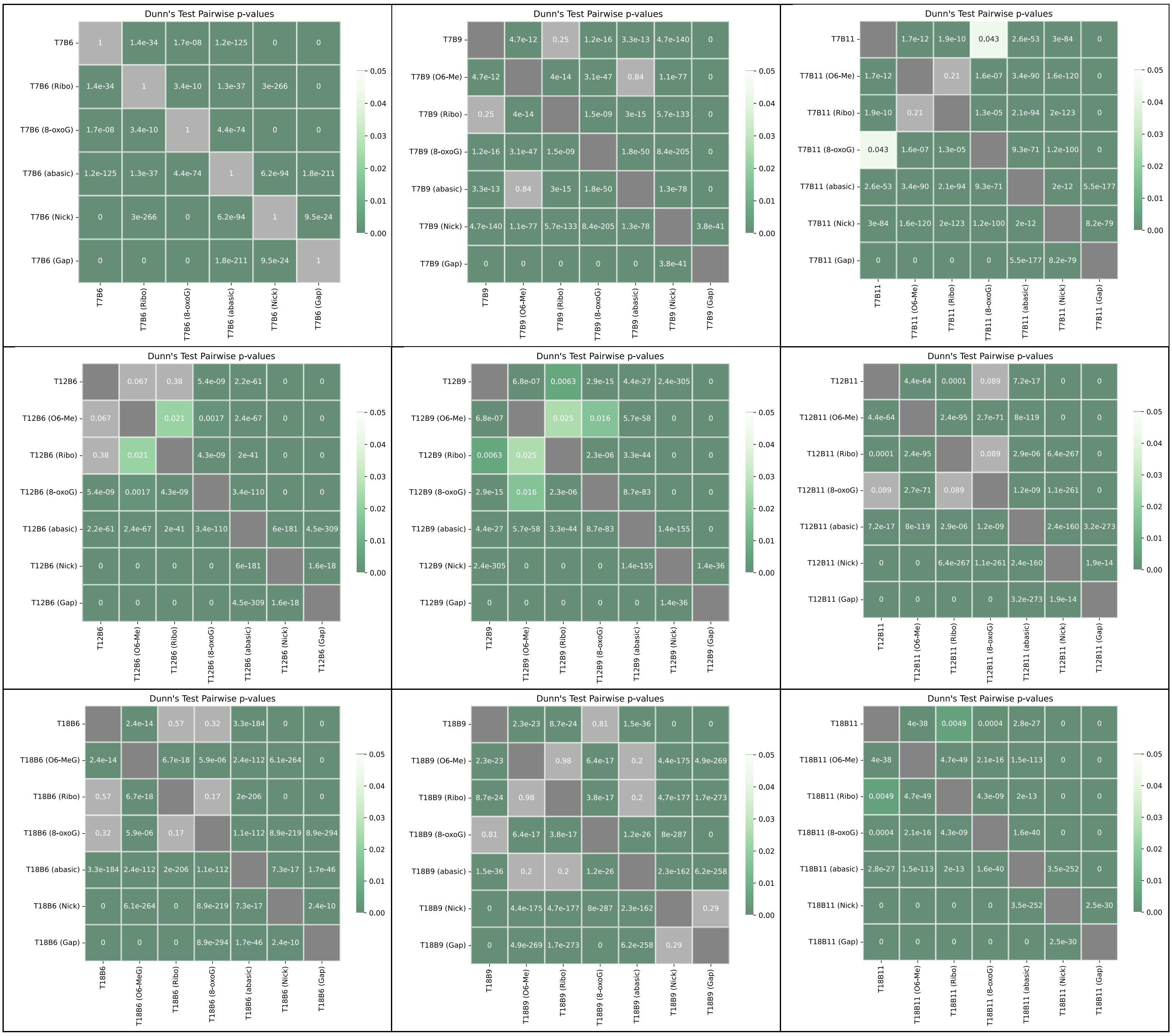

Supplementary Figure 29: Dunn’s Tests results for the combined repeats for each of the labelling positions for each of the different types of damage.

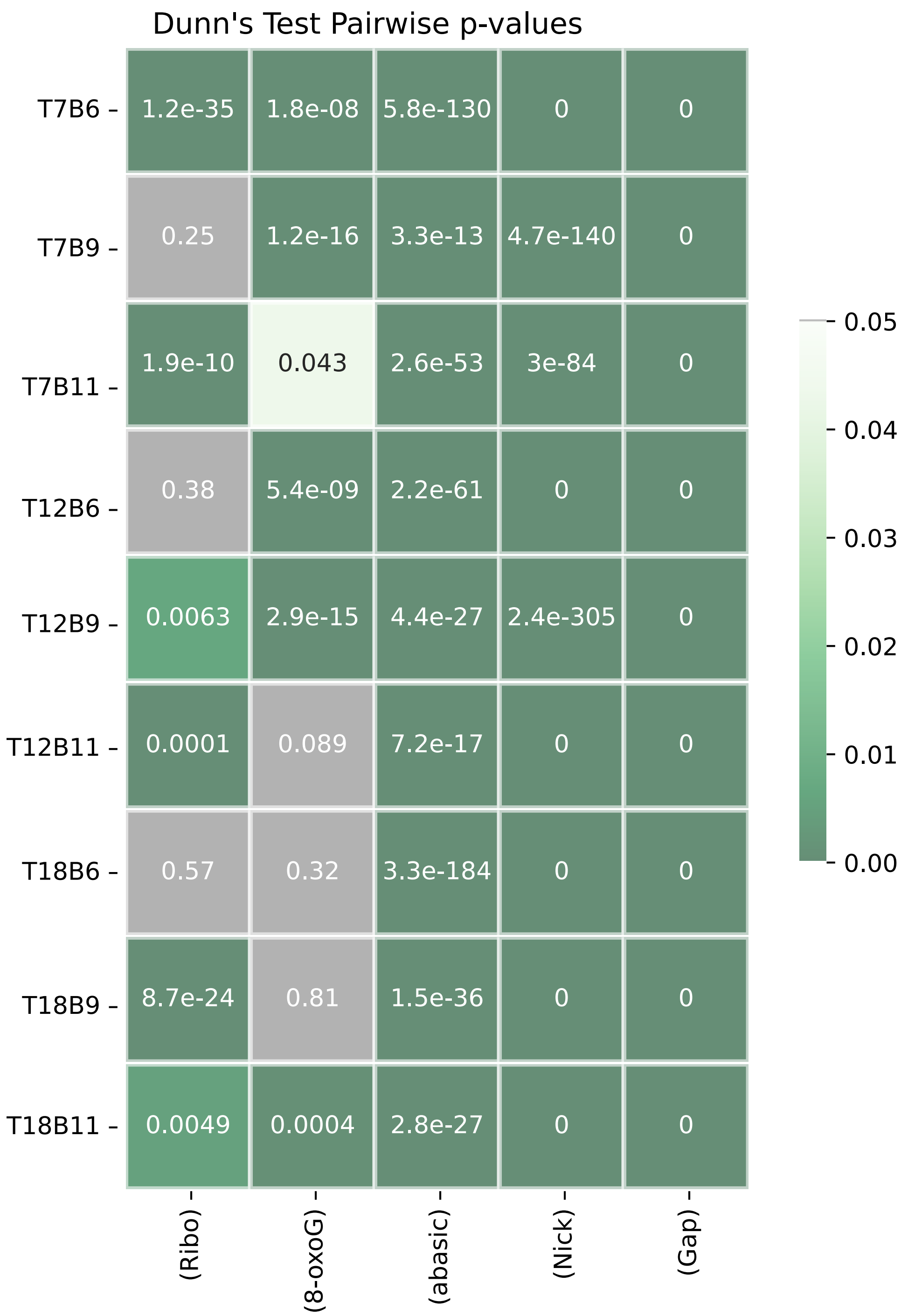

Supplementary Figure 30: All Dunn of each labelling positions with each of the different types of damage.

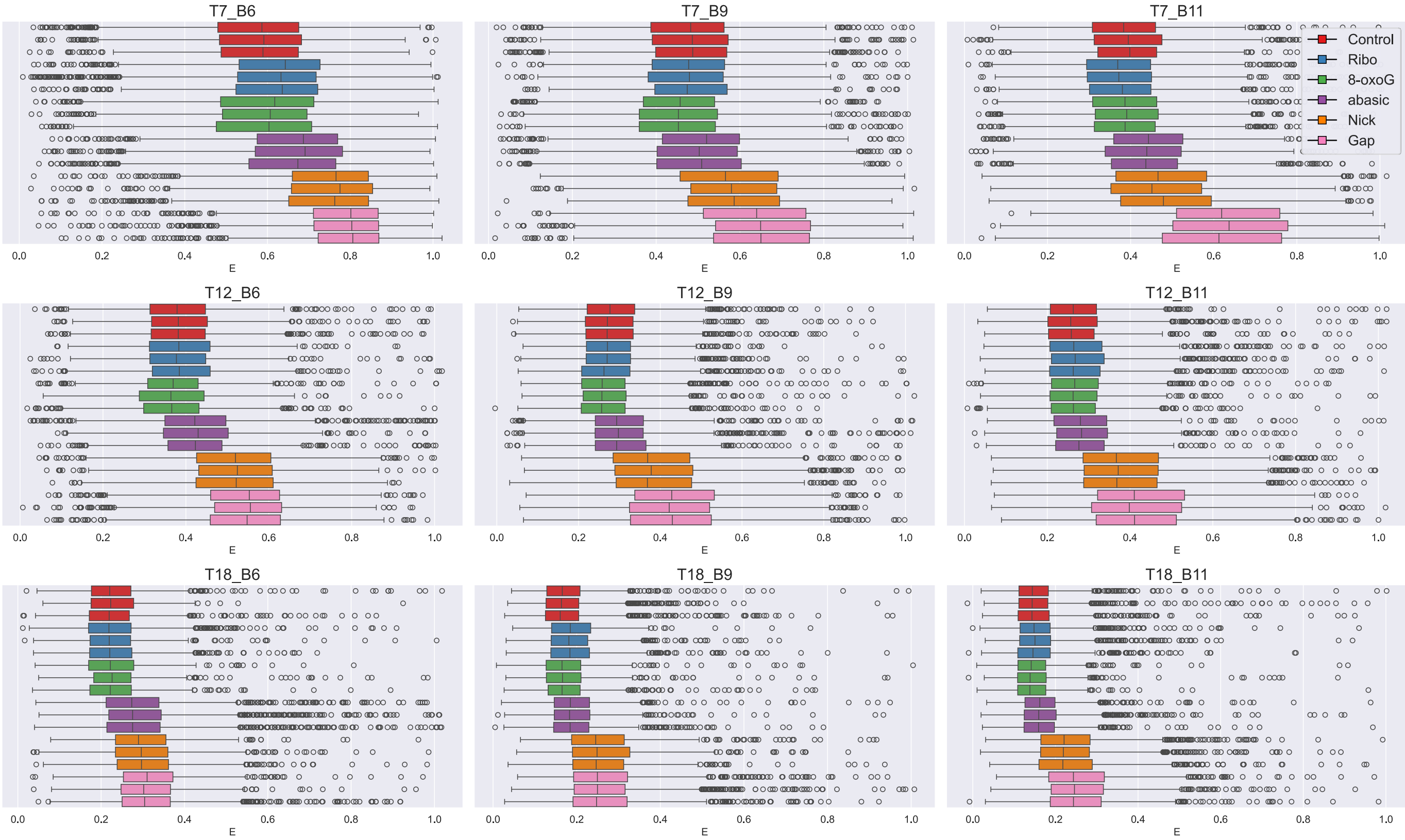

Supplementary Figure 31: Boxplots showing FRET efficiency (E) for each independent repeat of the seven duplex constructs for all dye-pair combinations.

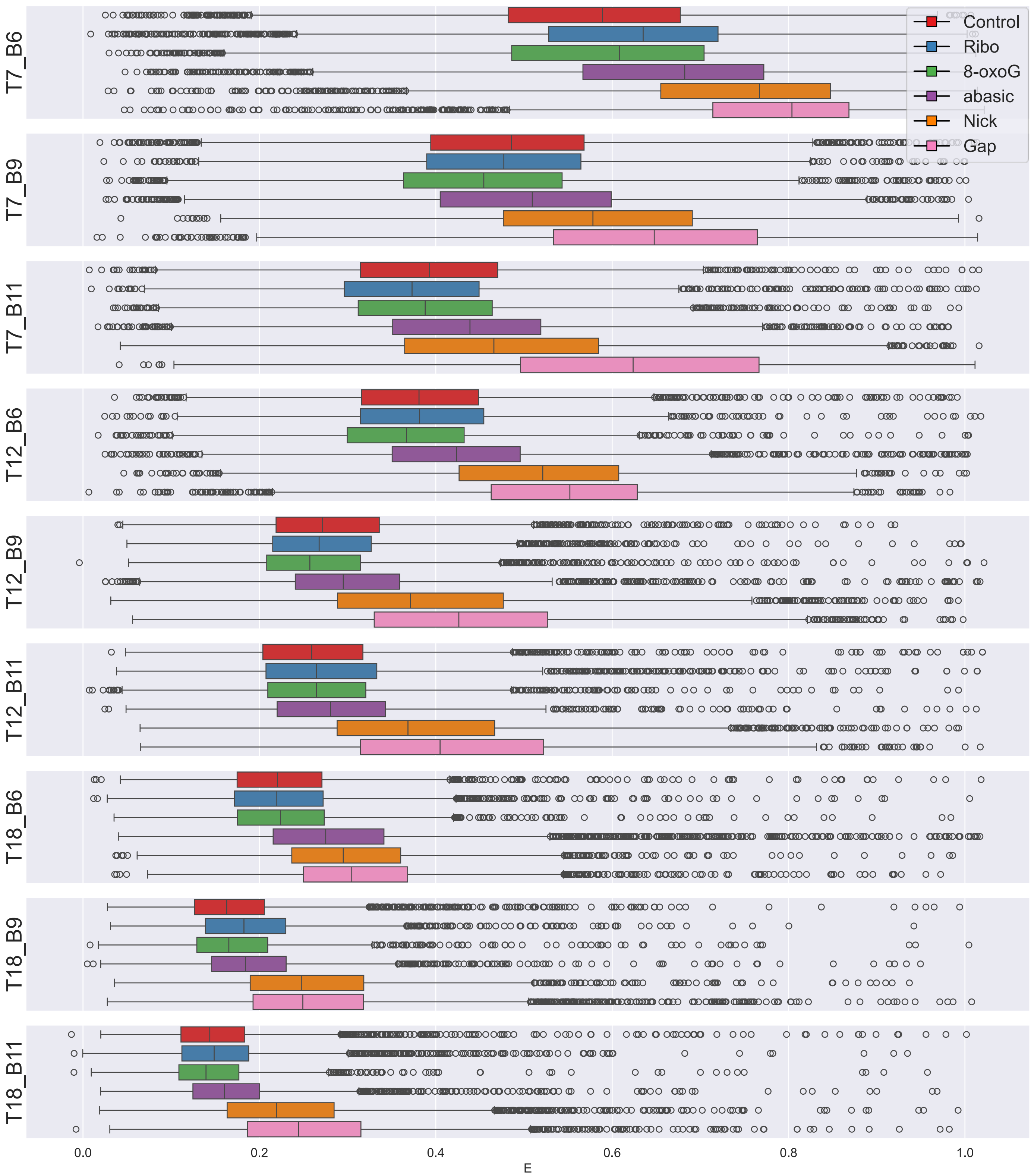

Supplementary Figure 32: Boxplots showing FRET efficiency (E) for the triplicate dataset for the seven duplex constructs for all dye-pair combination.

Example Quantum Yield calculation:

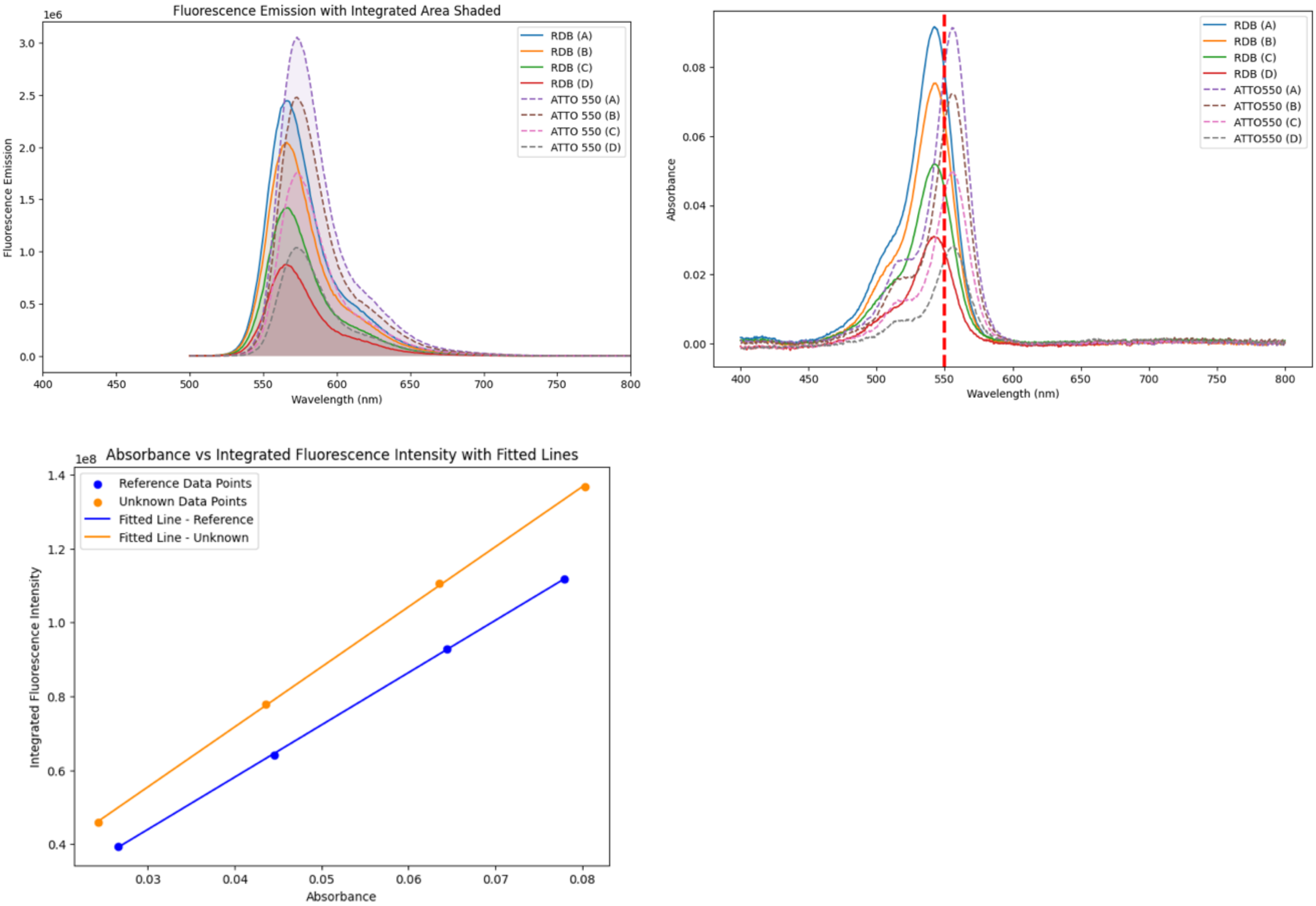

Reference data: y = 1480035464.96x + 6148414.91
R-squared: 0.9963
Unknown data: y = 1428523583.16x + 4156403.06
R-squared: 0.9993

Qr = 0.70
nr = 1.33
ns = 1.33
$Qs = Qr*(ms/mr)*(ns/nr)^2$
Unknown Sample's Quantum Yield: 0.8041

Supplementary Figure 33: Example quantum yield calculation with using RDB as reference dye to work out quantum yield of ATTO 550.

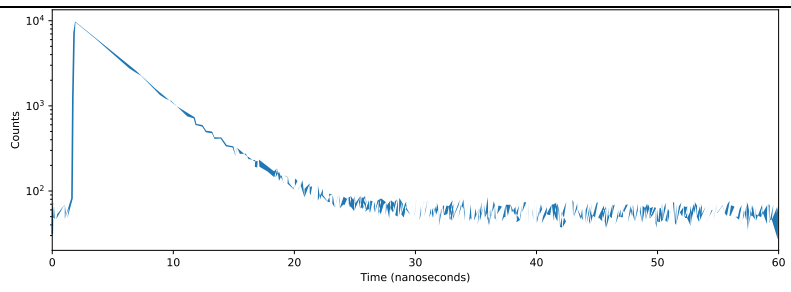
ATTO 550 T1

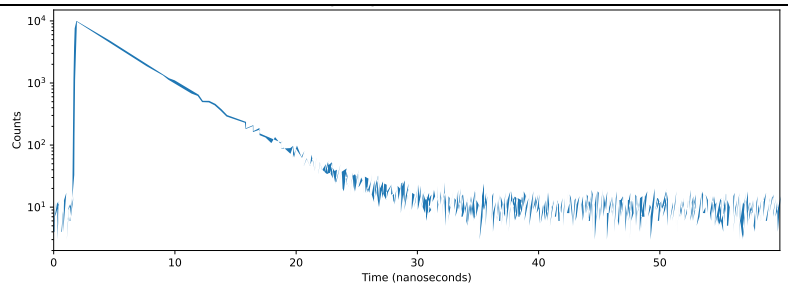
ATTO 550 T2

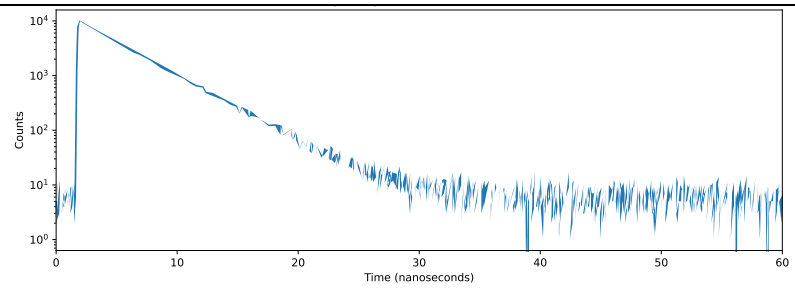
ATTO 550 T3

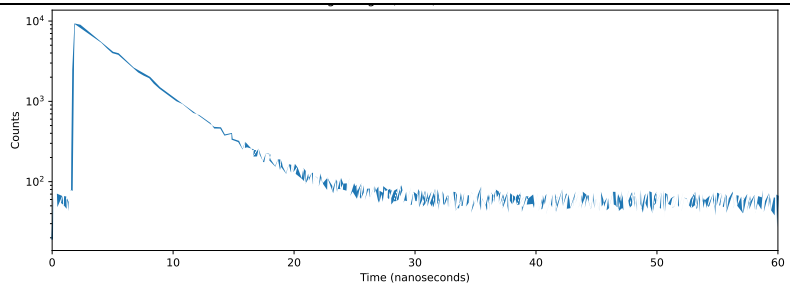
NMS1 T1

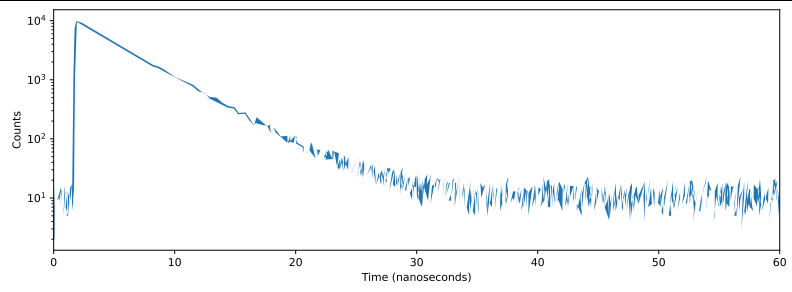
NMS1 T2

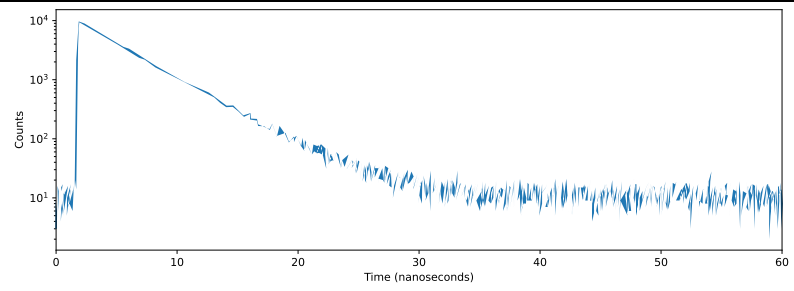
NMS1 T3

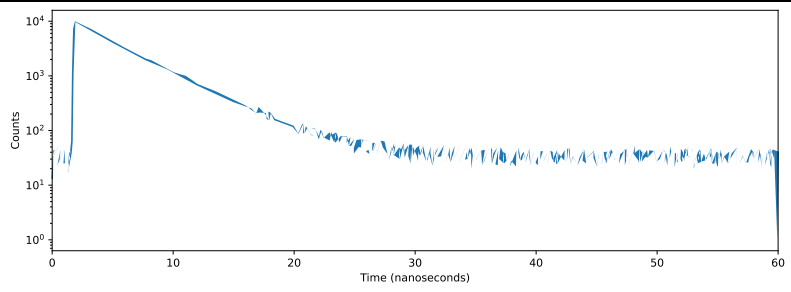
NMS2 T1

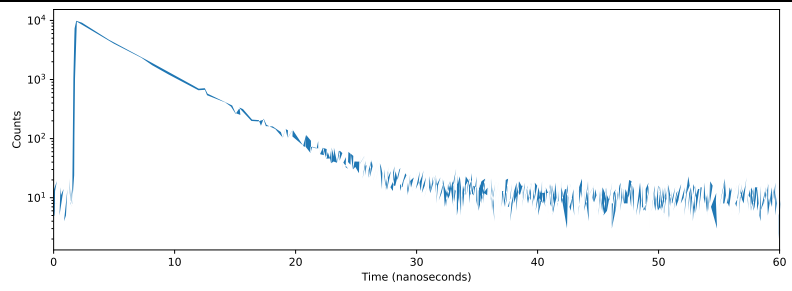
NMS2 T2

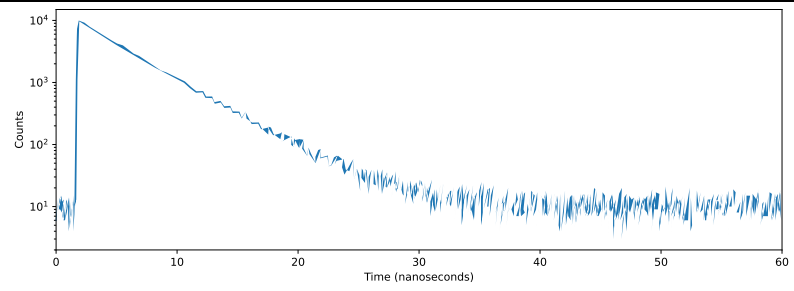
NMS2 T3

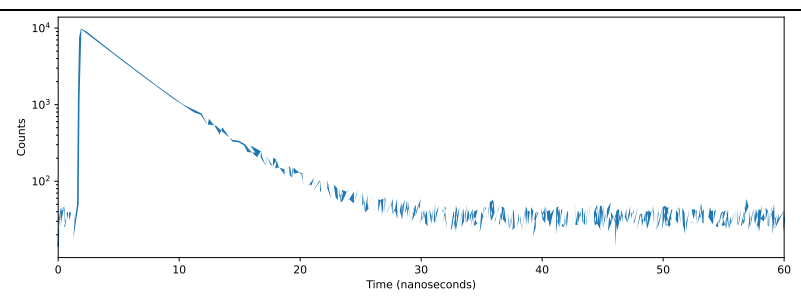
NMS3 T1

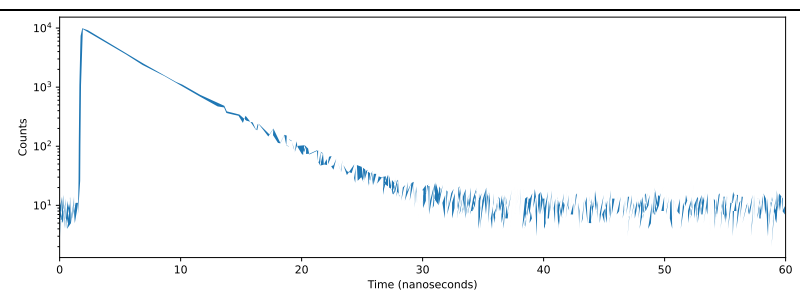
NMS3 T2

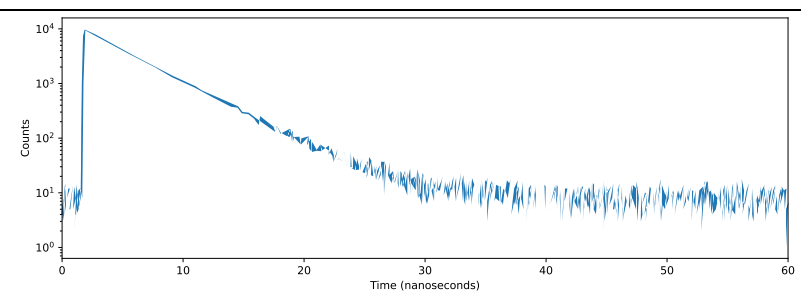
NMS3 T3

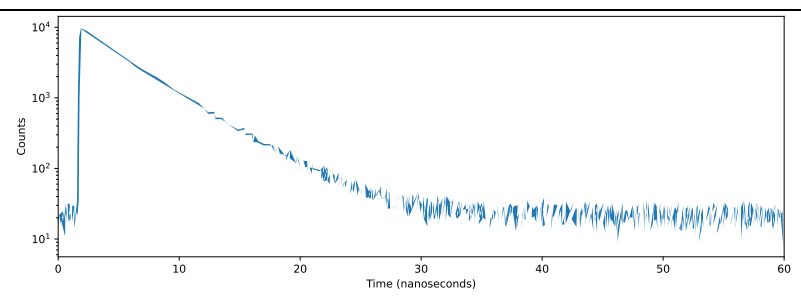
T-7 T1

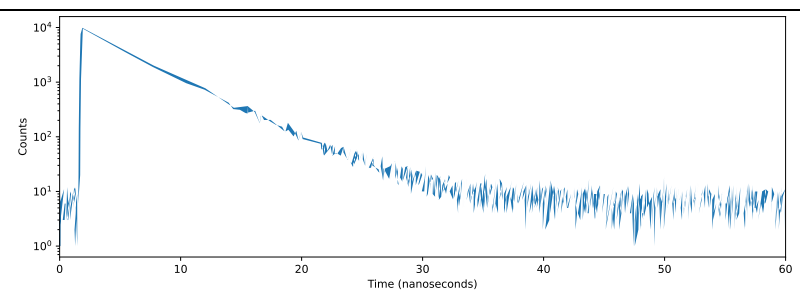
T-7 T2

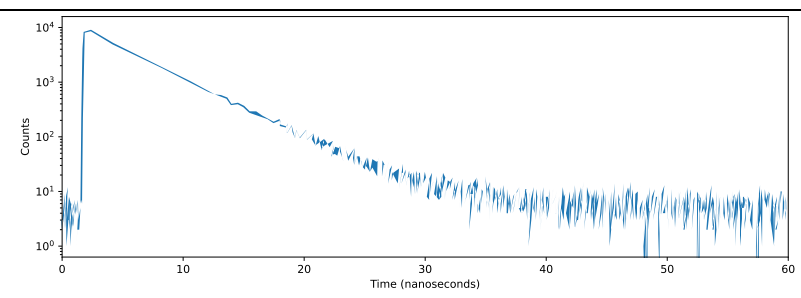
T-7 T3

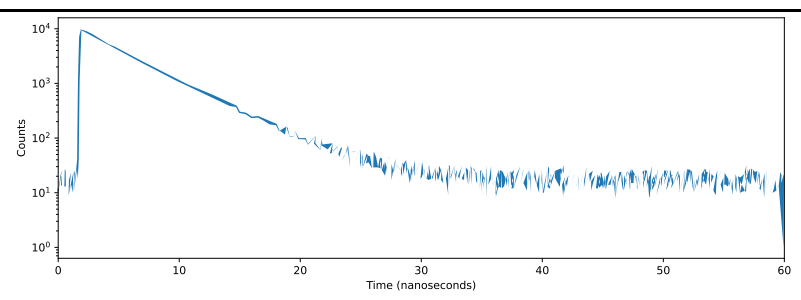
T-12 T1

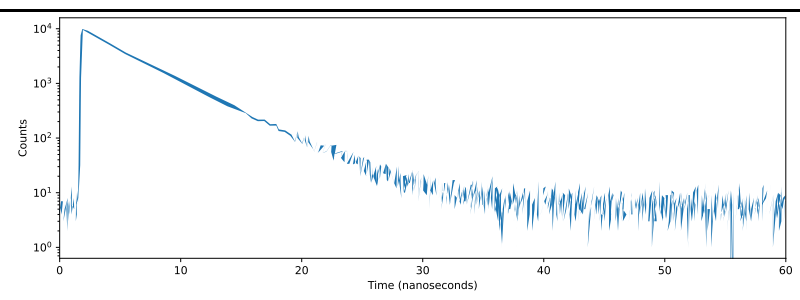
T-12 T2

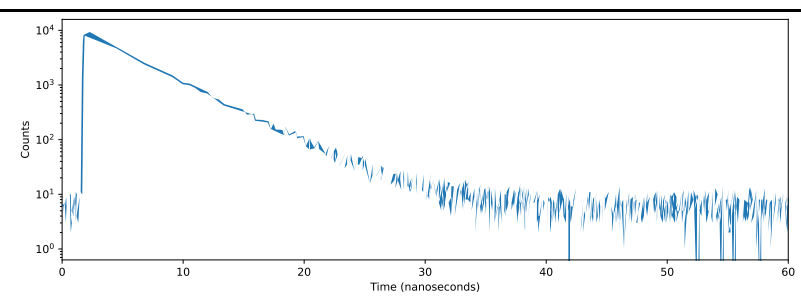
T-12 T3

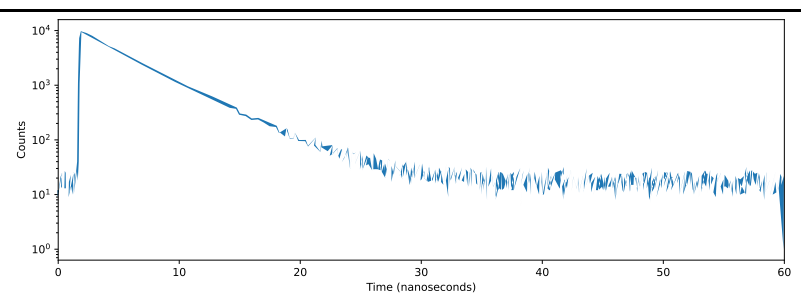
T-18 T1

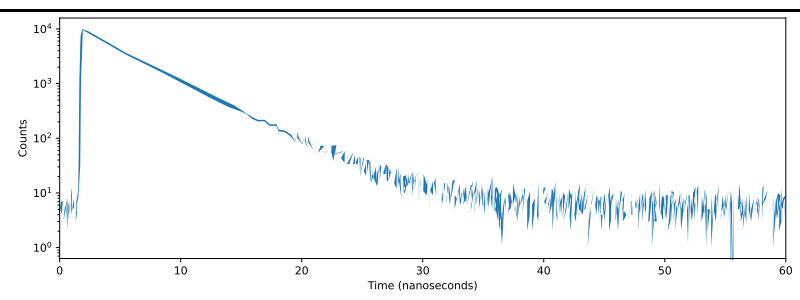
T-18 T2

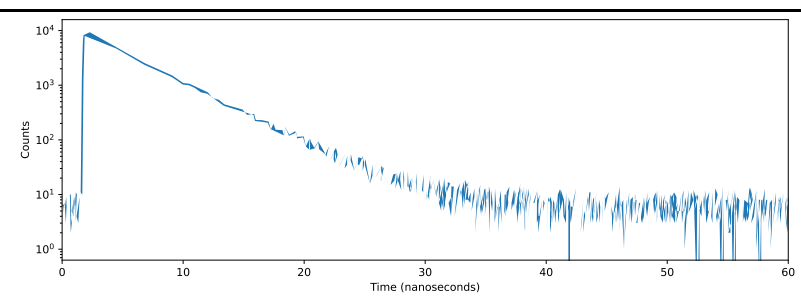
T-18 T3

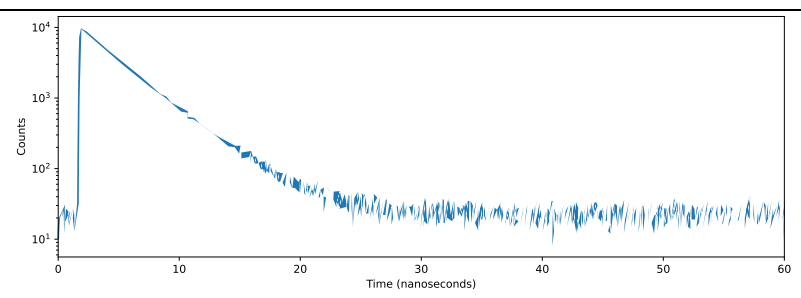
NMS1+4 T1

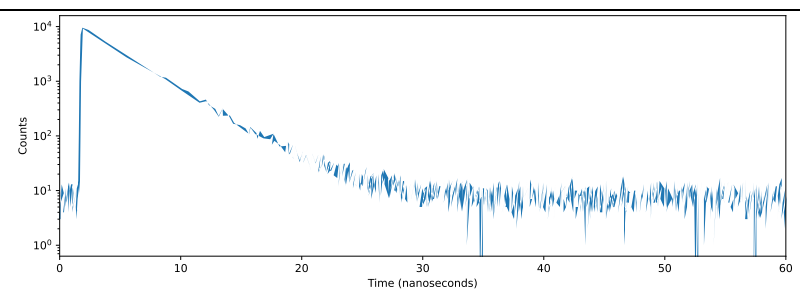
NMS1+4 T2

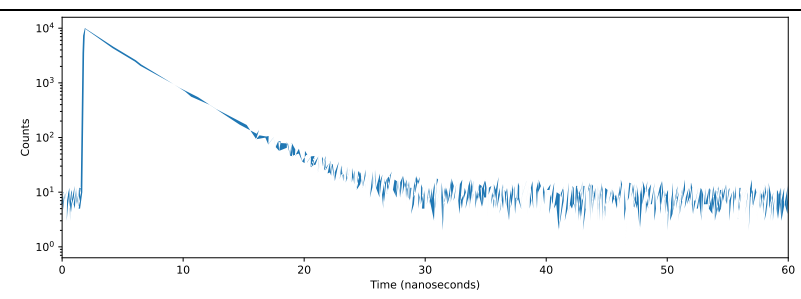
NMS1+4 T3

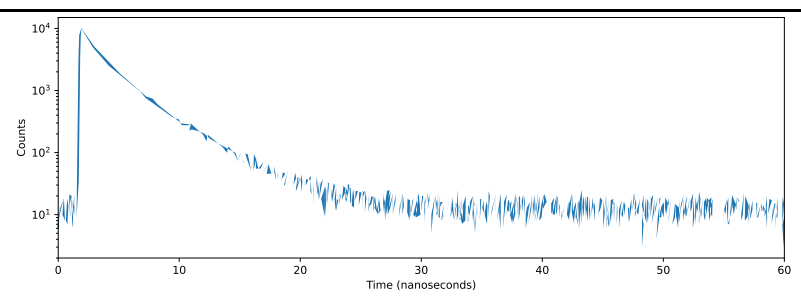
NMS2+4 T1

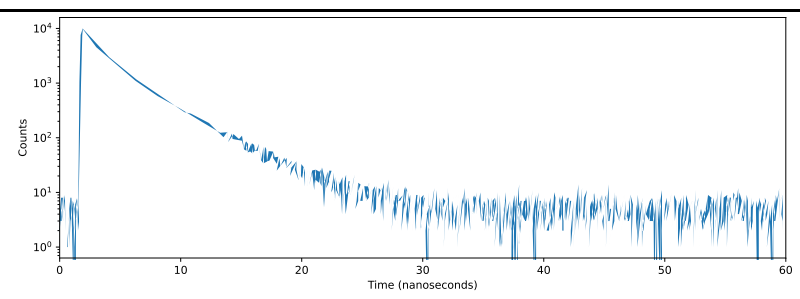
NMS2+4 T2

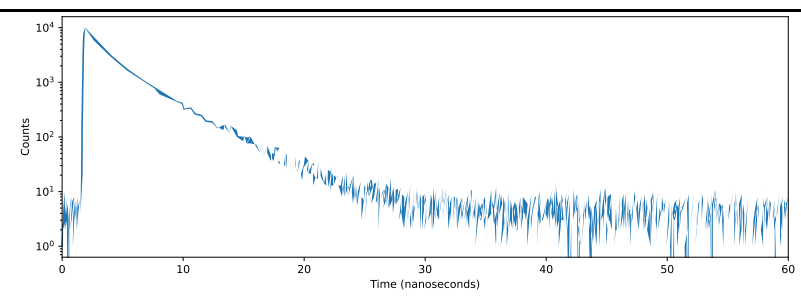
NMS2+4 T3

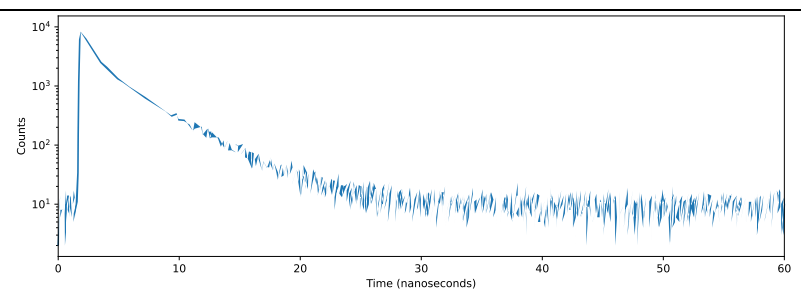
NMS3+4 T1

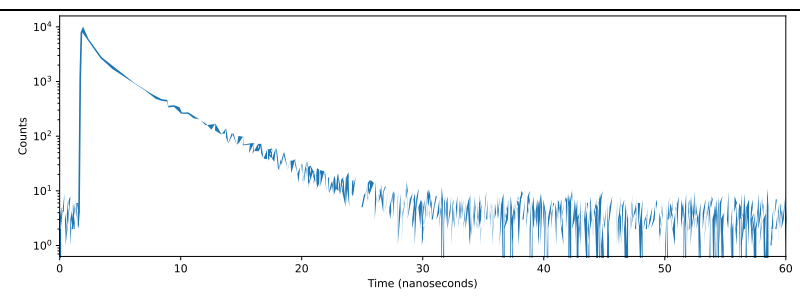
NMS3+4 T2

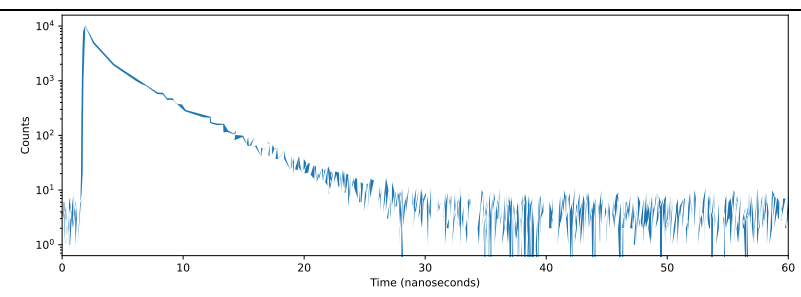
NMS3+4 T3

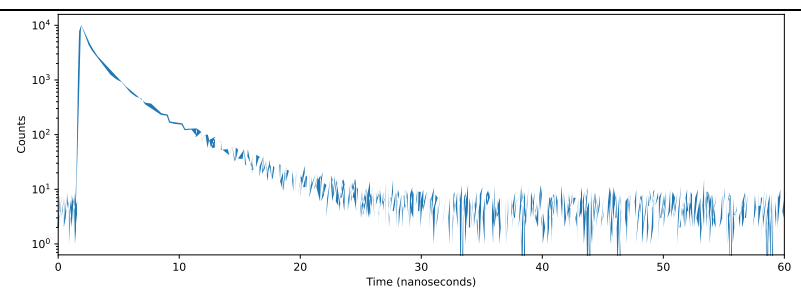
T-7 B+6 T1

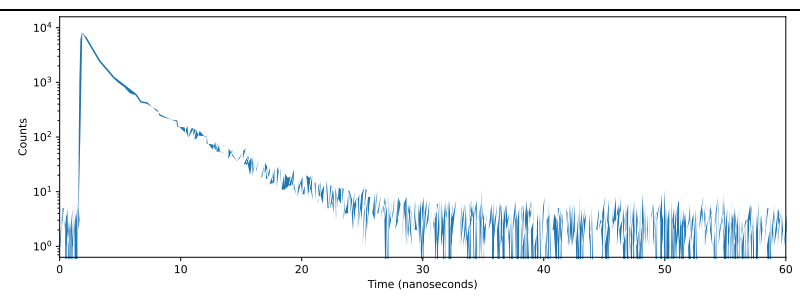
T-7 B+6 T2

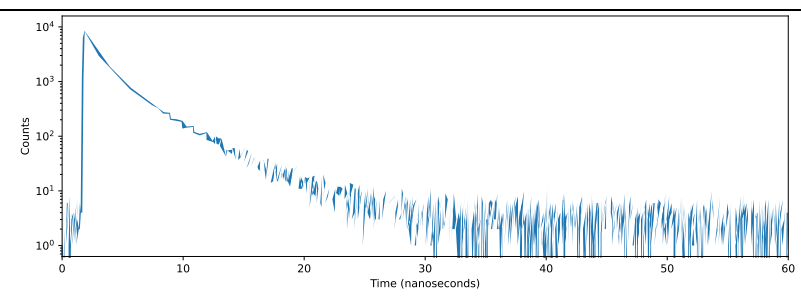
T-7 B+6 T3

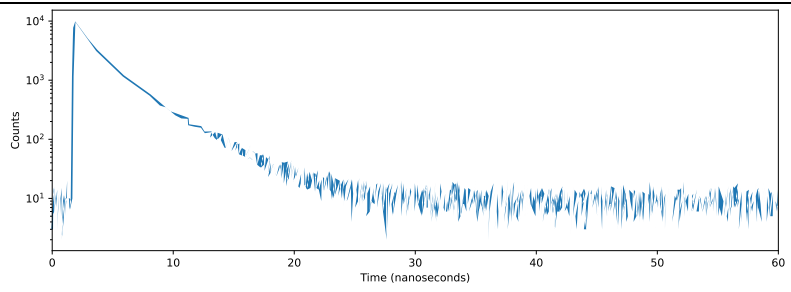
T-7 B+9 T1

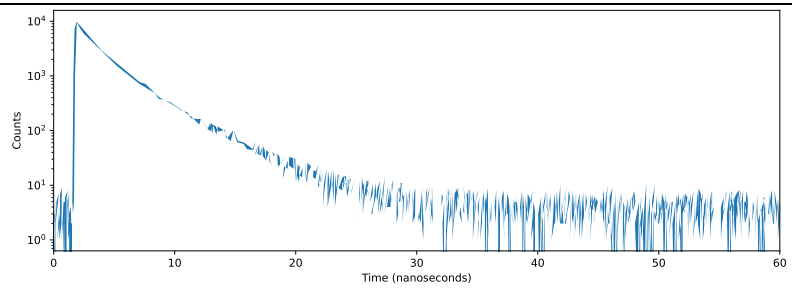
T-7 B+9 T2

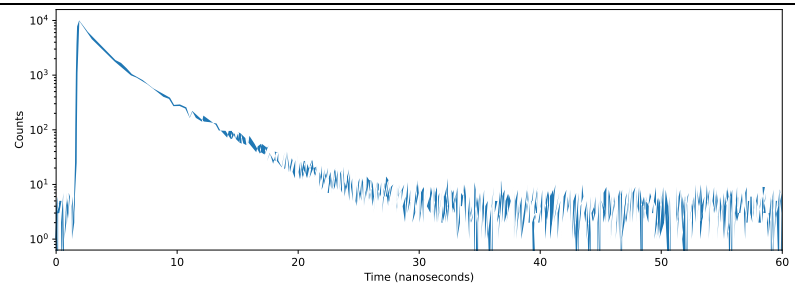
T-7 B+9 T3

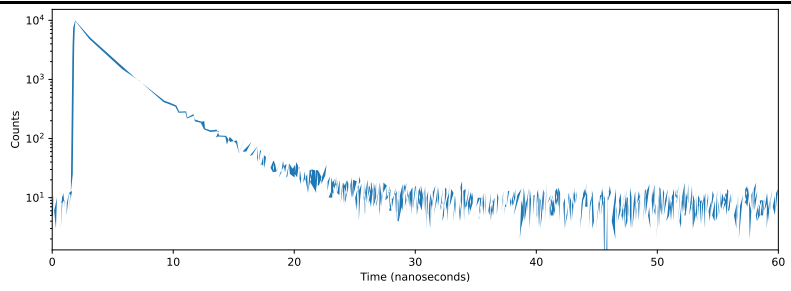
T-7 B+11 T1

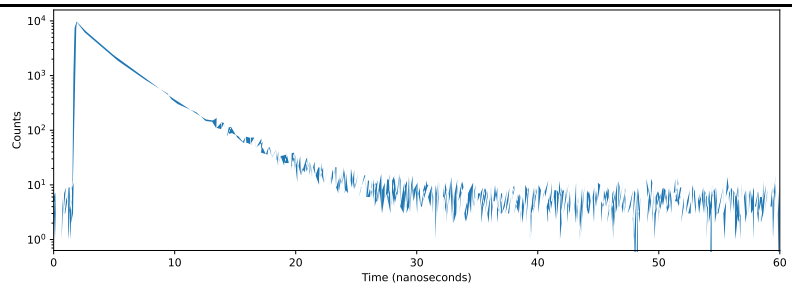
T-7 B+11 T2

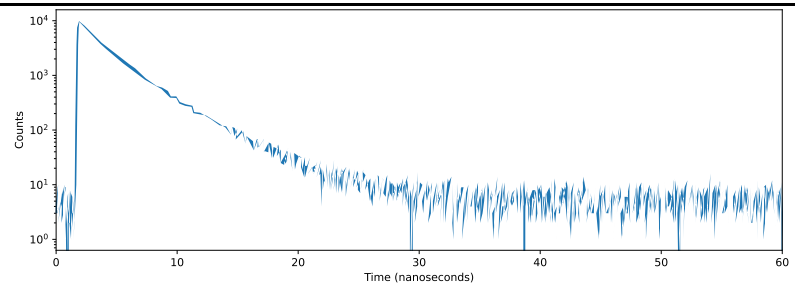
T-7 B+11 T3

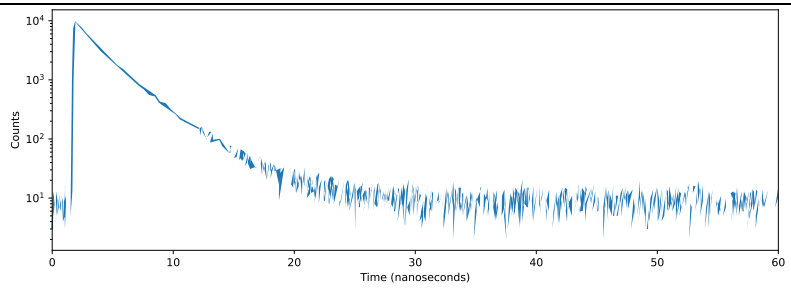
T-12 B+6 T1

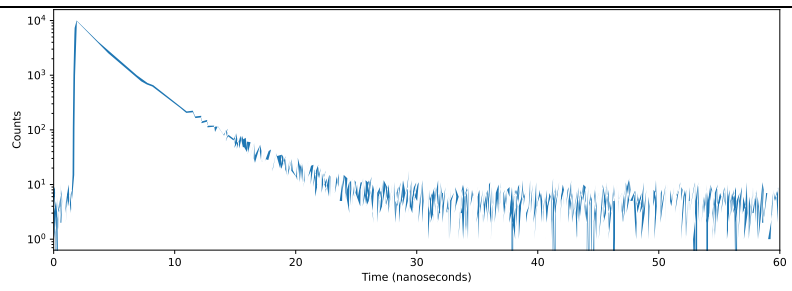
T-12 B+6 T2

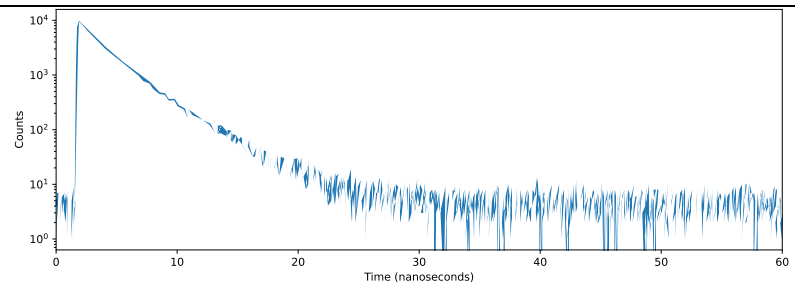
T-12 B+6 T3

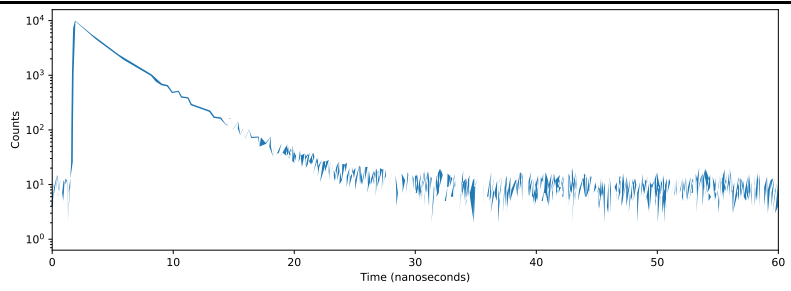
T-12 B+9 T1

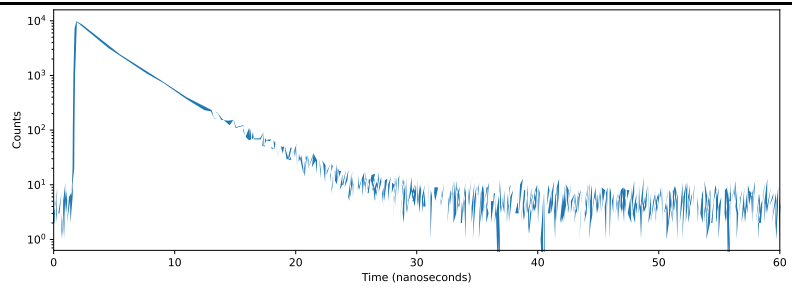
T-12 B+9 T2

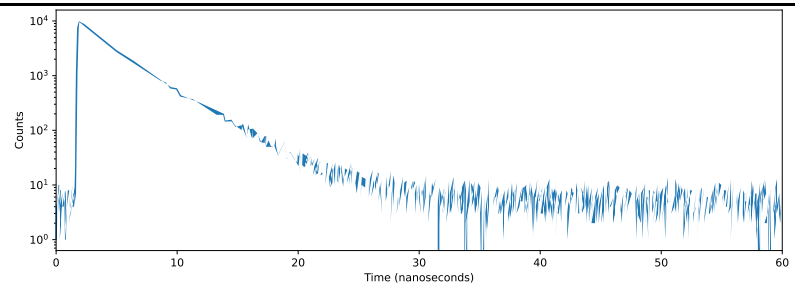
T-12 B+9 T3

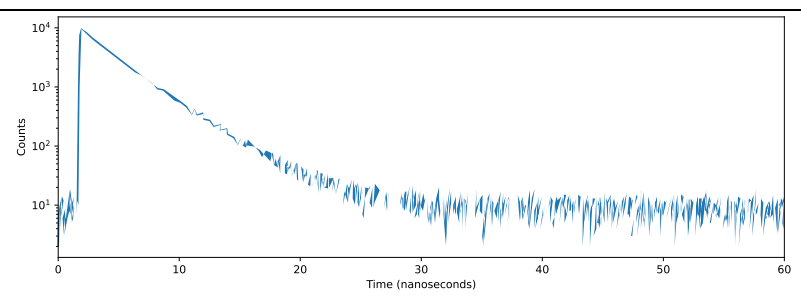
T-12 B+11 T1

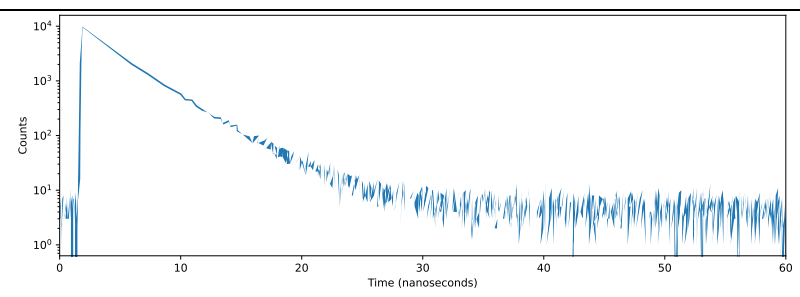
T-12 B+11 T2

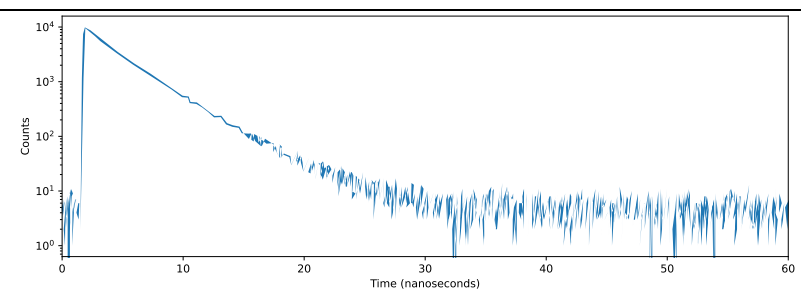
T-12 B+11 T3

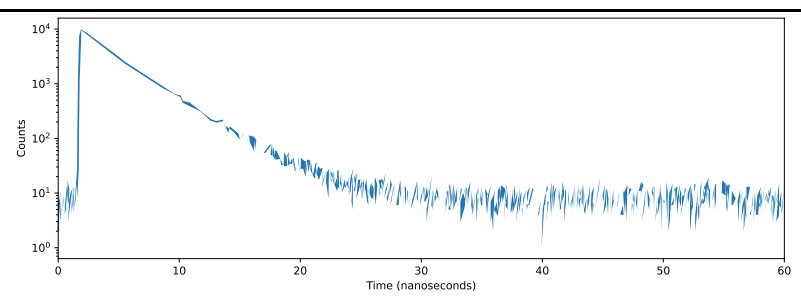
T-18 B+6 T1

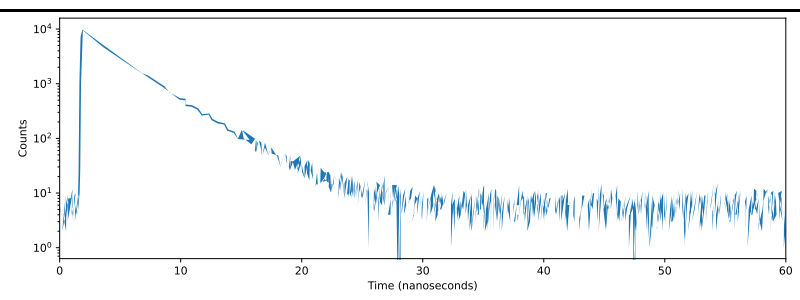
T-18 B+6 T2

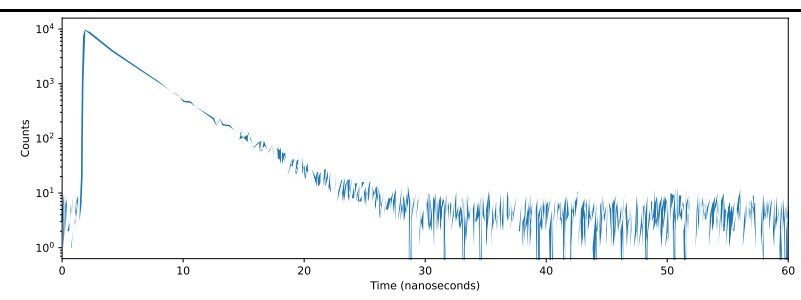
T-18 B+6 T3

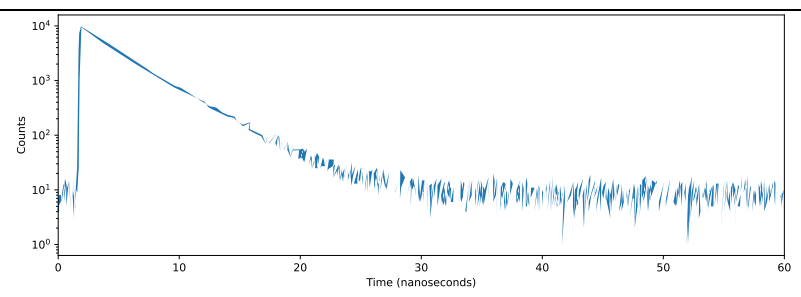
T-18 B+9 T1

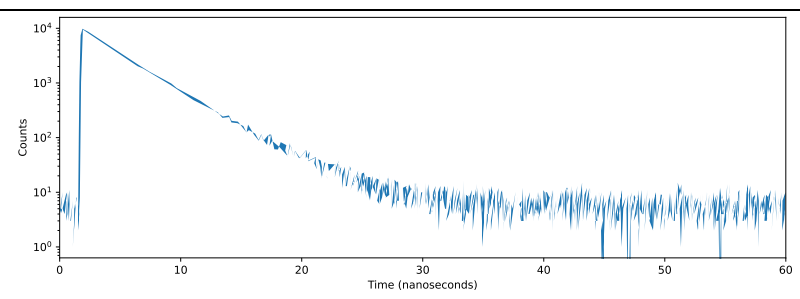
T-18 B+9 T2

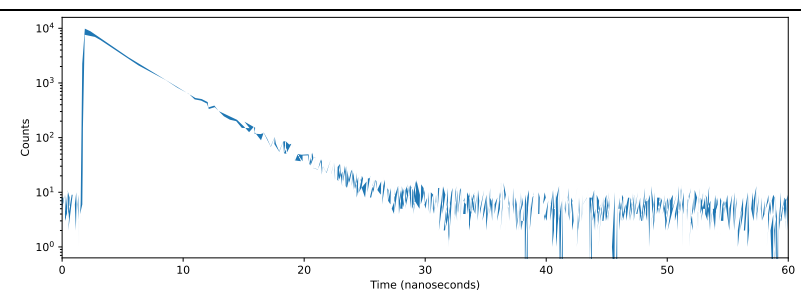
T-18 B+9 T3

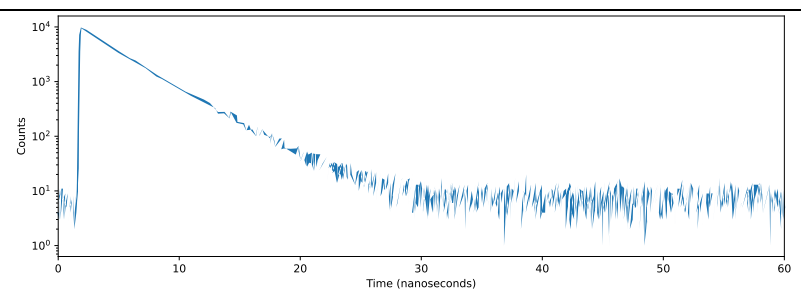
T-18 B+11 T1

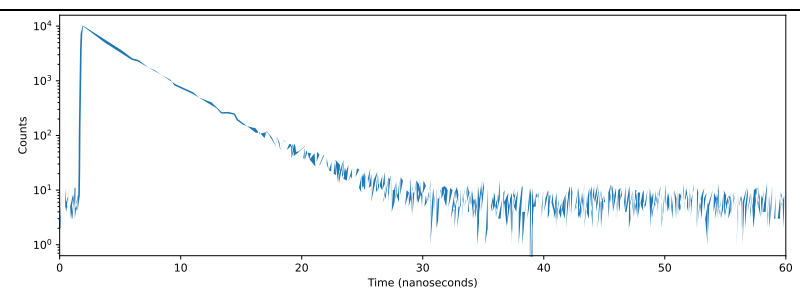
T-18 B+11 T2

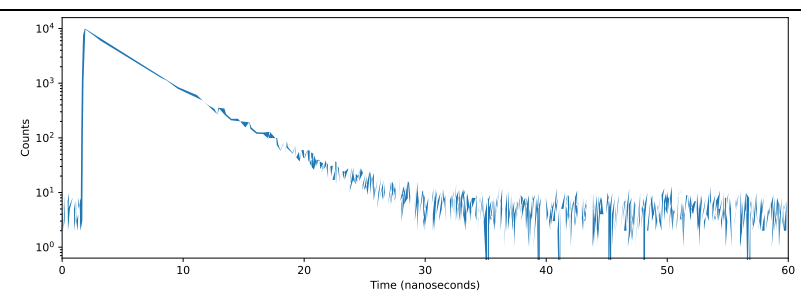
T-18 B+11 T3

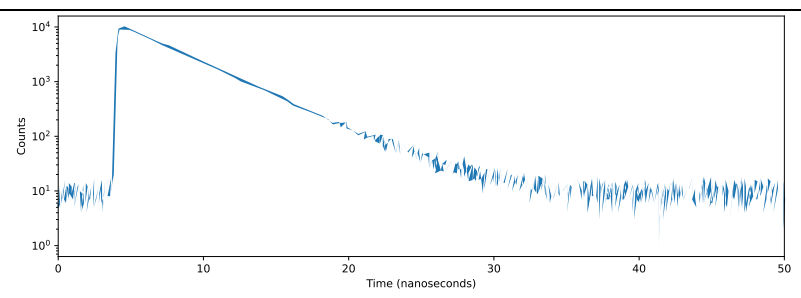
ATTO 647N T1

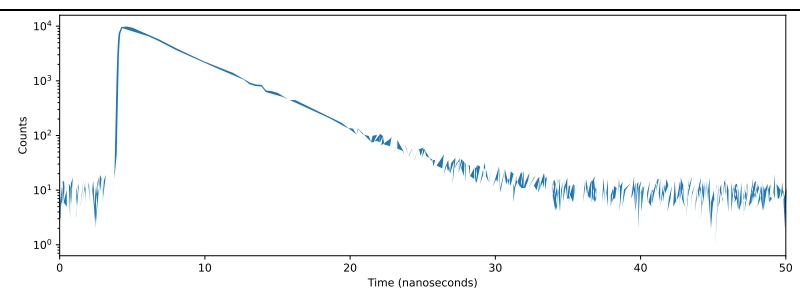
ATTO 647N T2

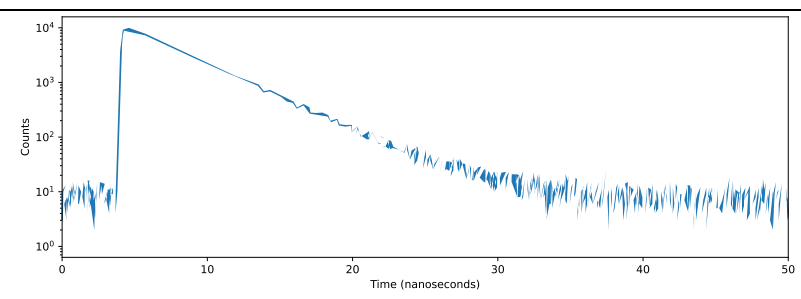
ATTO 647N T1

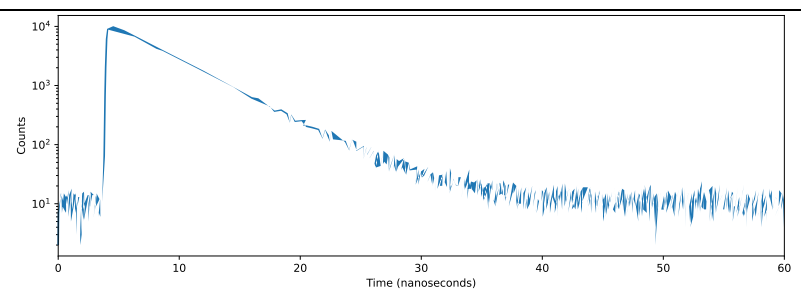
NMS4 T1

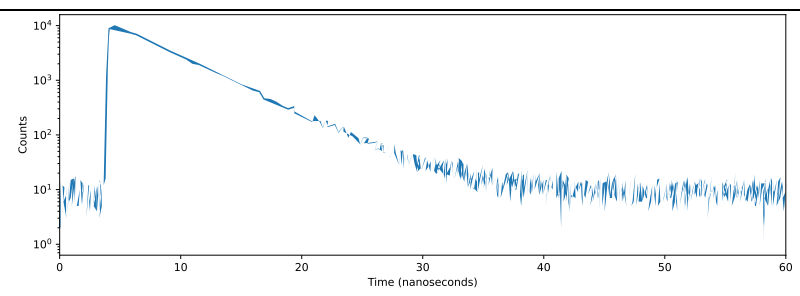
NMS4 T2

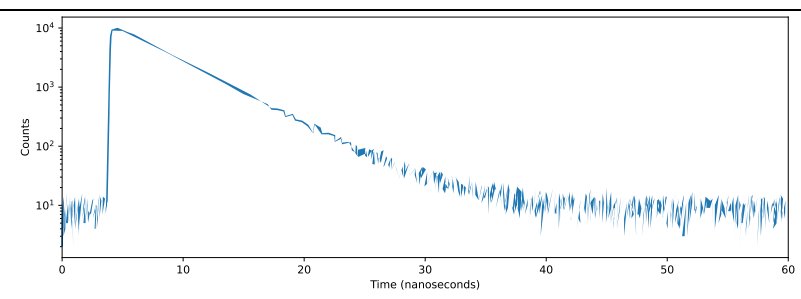
NMS4 T3

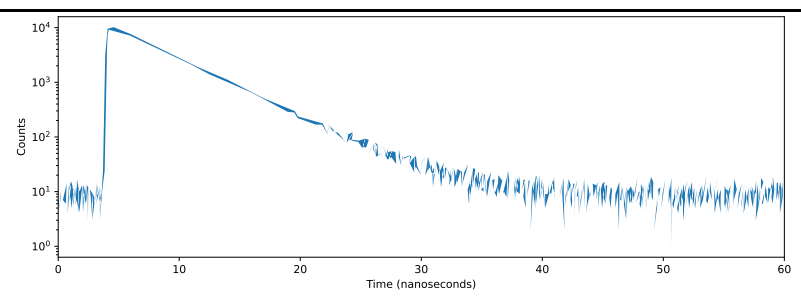
B+6 T1

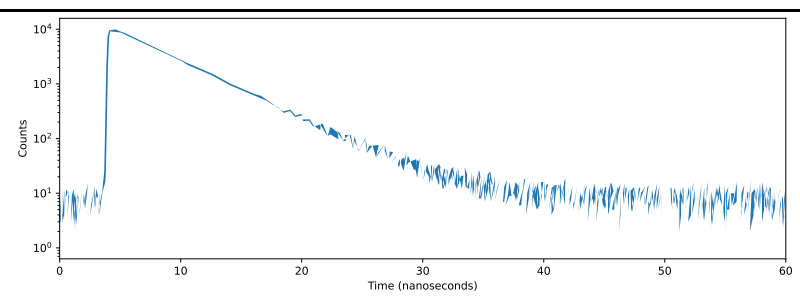
B+6 T2

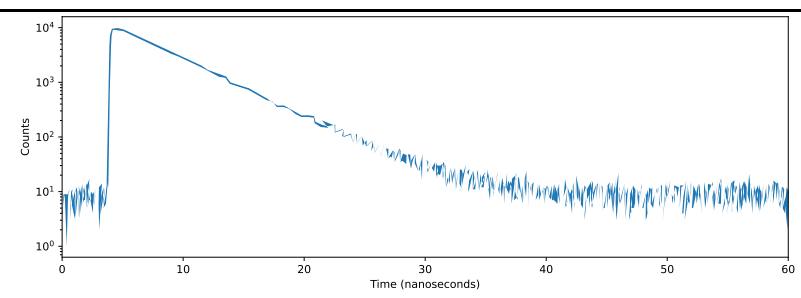
B+6 T3

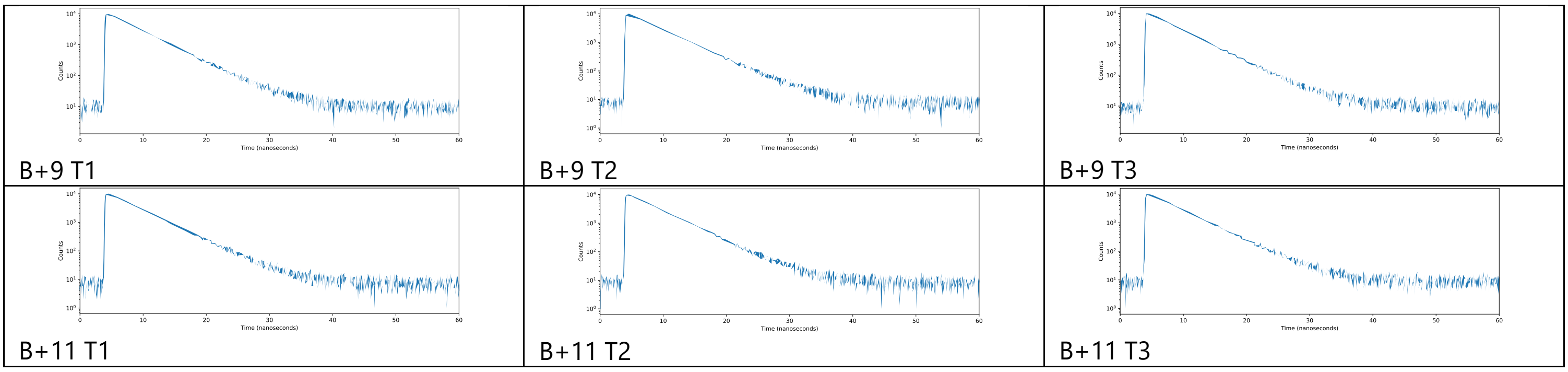

Supplementary Figure 34: Lifetime measured with 3 repeats for free dyes and each single labelled duplex DNA.

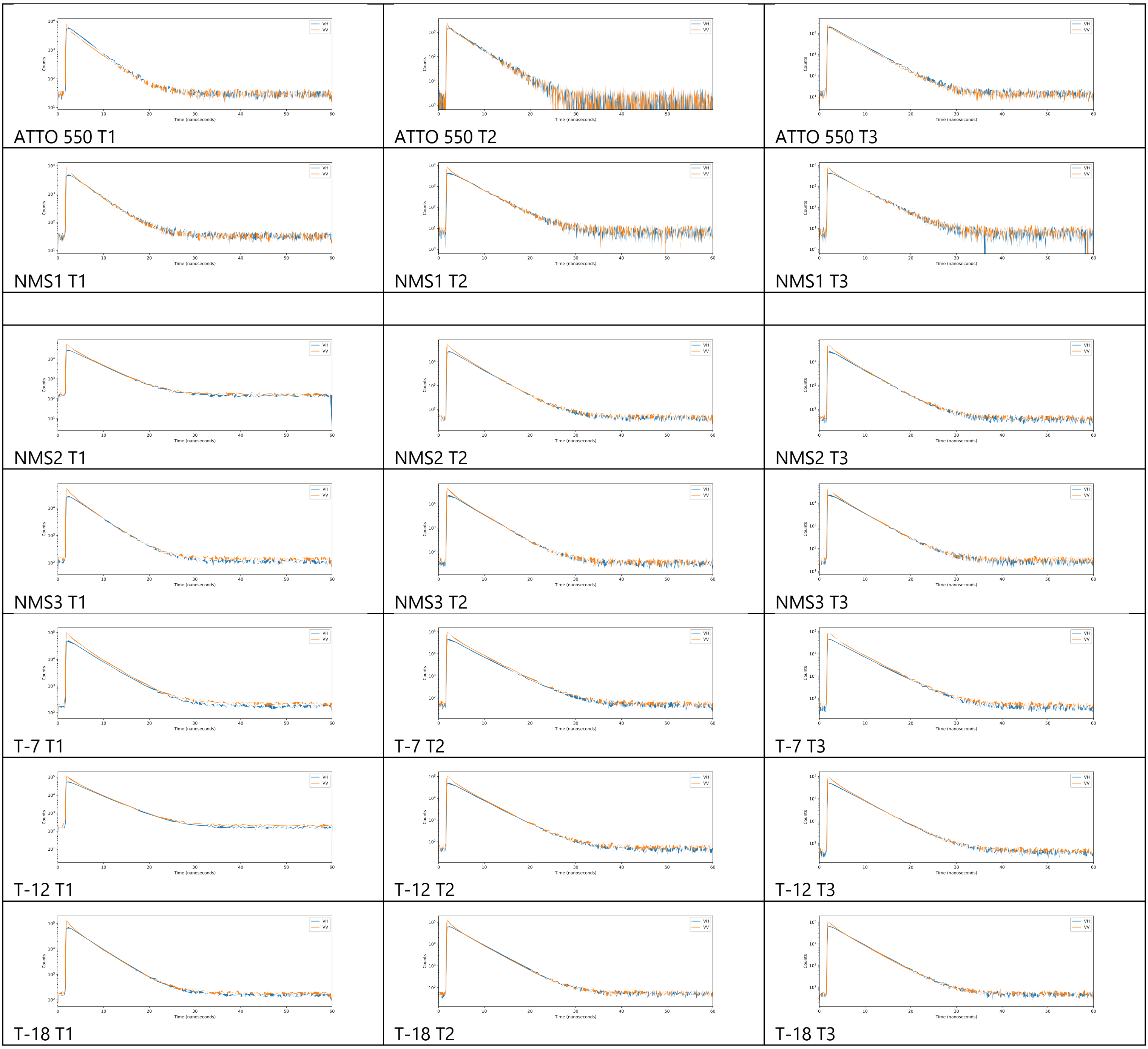

Supplementary Figure 35: VH and VV measurements for each of the free dyes and single labelled DNA duplex.

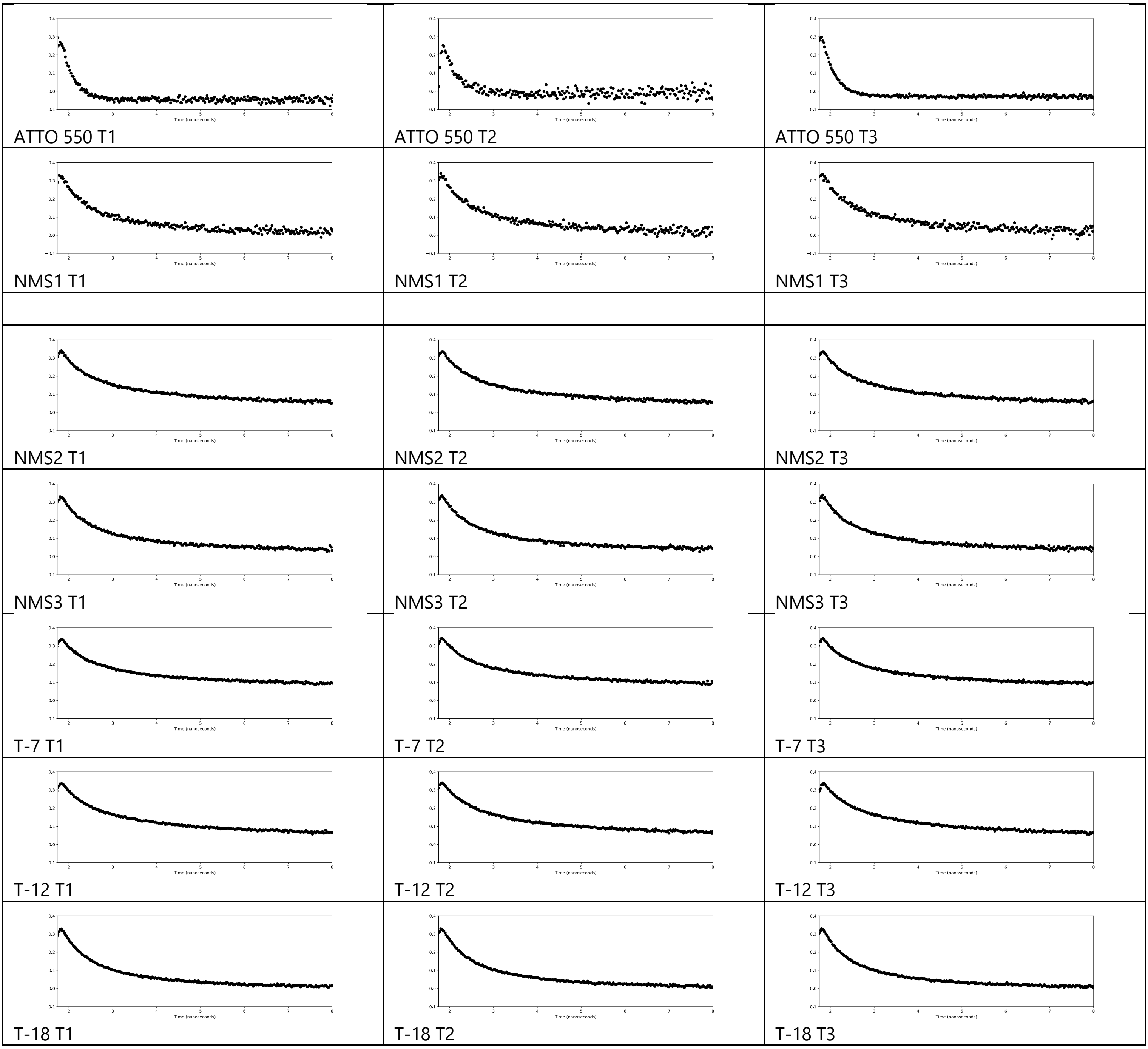

Supplementary Figure 36: Anisotropy decay for each of the free dyes and single labelled DNA duplex.

Ribo vs Control

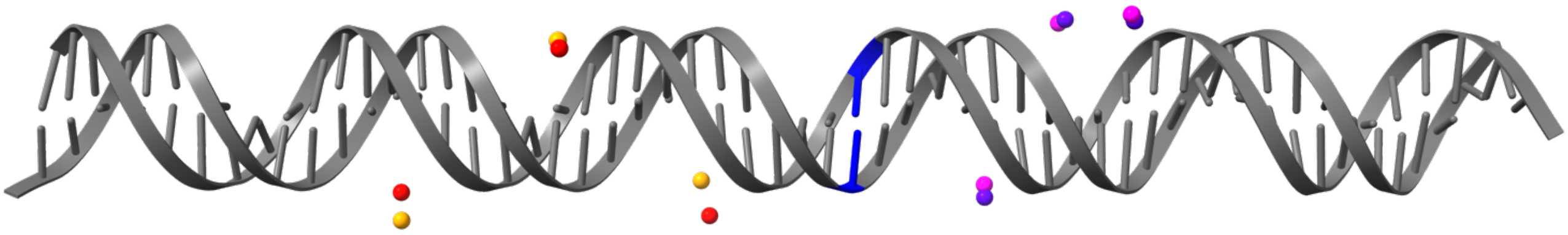

8-oxoG vs Control

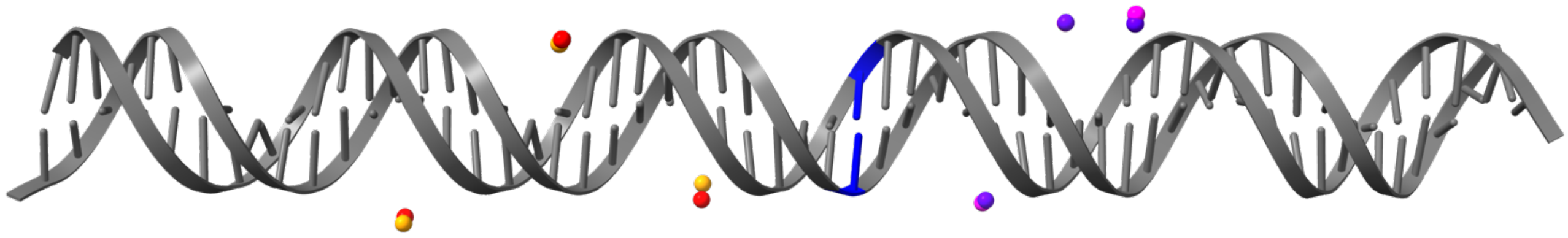

Abasic vs Control

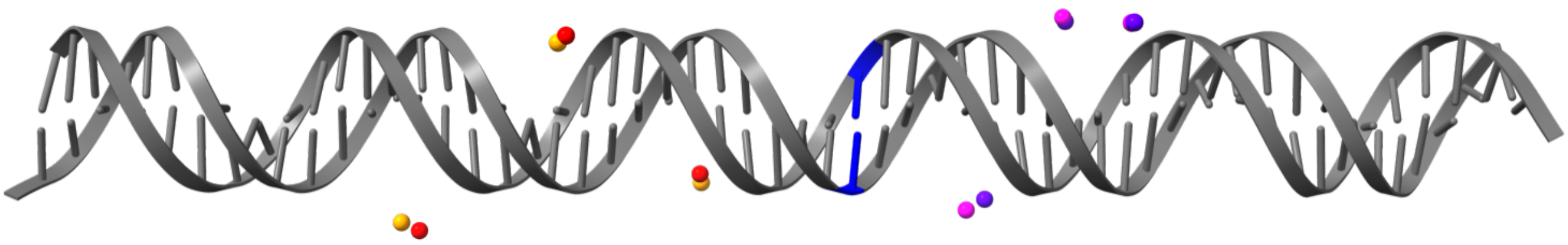

Nick vs Control

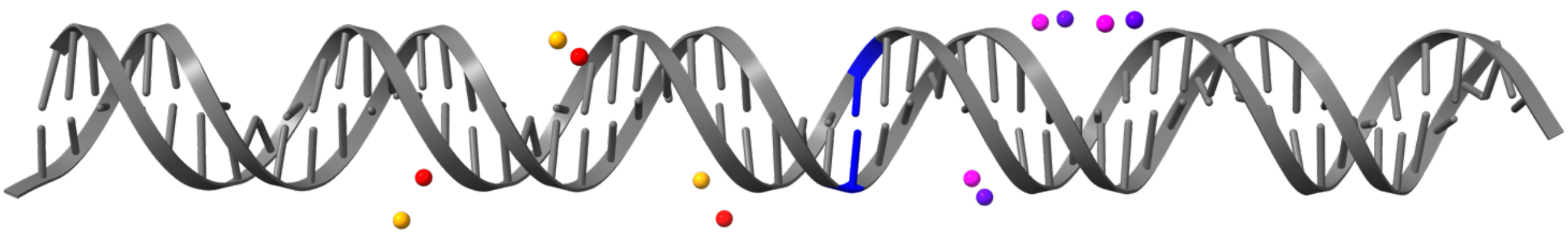

Gap vs Control

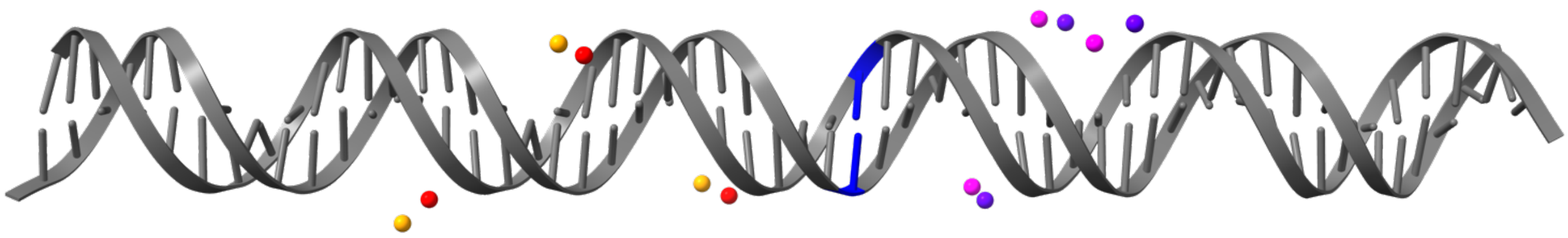

Supplementary Figure 37: Schematic showing the different type of damage position (blue) and how this has affected the mean position of the AV cloud with control position of donor ATTO 550 in pale orange and moved position in dark orange/red and for control position of acceptor ATTO 647N in dark purple and moved position in pink.

Construct	Bend (°) Left	Bend (°) Right	Total Angle (°) Left	Total Angle (°) Right	Twist (°) Left	Twist (°) Right	RMSD Left (Å)	RMSD Right (Å)	RMSD Total (Å)
Ribo	12.17	3.68	12.45	3.91	−2.64	−1.32	3.419	1.540	2.652
8-oxoG	9.90	3.10	10.18	3.22	−2.38	−0.86	3.071	1.007	2.285
Abasic	2.04	1.11	5.04	3.14	−4.61	−2.94	2.431	1.608	2.061
Nick	10.88	2.61	12.74	3.22	+6.62	−1.88	4.730	2.972	3.950
Gap	5.49	5.74	5.88	5.77	−2.12	−0.51	3.813	3.791	3.802

Supplementary Figure 38: Bending and twisting angles were determined from duplex models of chemically and structurally modified constructs relative to the control (unmodified) duplex shown in red. Positive twist values indicate over-twisting and negative values indicate under-twisting relative to the control. Left and right refer to the respective helical halves flanking the lesion site. RMSD values represent the deviation of each half from the control structure following alignment of the undamaged region.

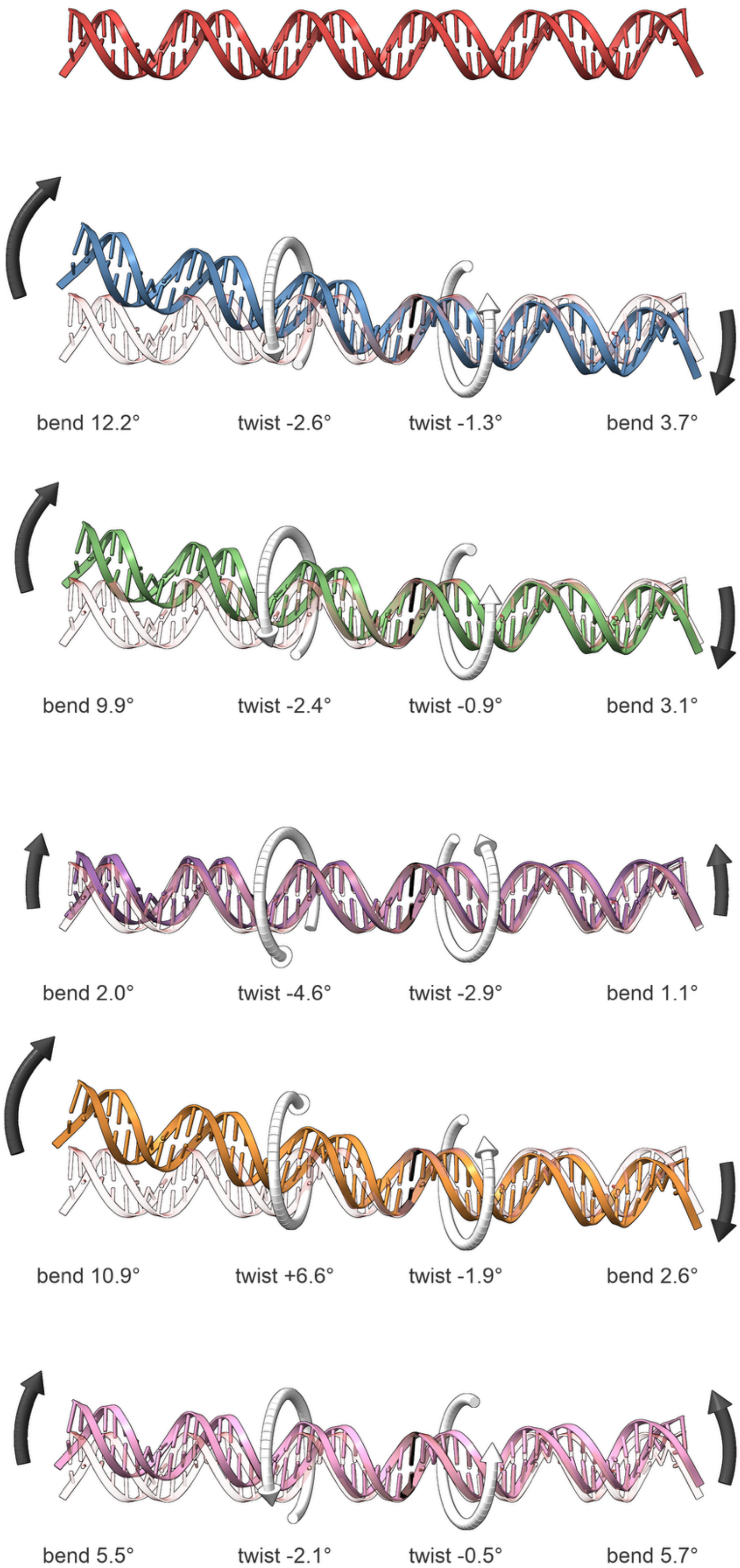

Supplementary Figure 39: Bending and twisting angles were determined from duplex models of chemically and structurally modified constructs relative to the control (unmodified) duplex shown in red. Positive twist values indicate over-twisting and negative values indicate under-twisting relative to the control. Left and right refer to the respective helical halves flanking the lesion site.
